# Emerging perspectives in proteostasis: bridging mechanisms and therapeutics for human diseases

**DOI:** 10.1038/s41392-026-02714-4

**Published:** 2026-06-09

**Authors:** Ankush Borlepawar, Marco Neu, Ziqi Ma, Anushka Deshpande, Hannah Bühringer, Parvana Hajieva, Norbert Frey, Ashraf Yusuf Rangrez

**Affiliations:** 1 Cellular Adaptation and Bioenergetics Group, Institute for Translational Medicine, Medical School Hamburg, Hamburg, Germany; 2 Department of Cardiology, Angiology and Pneumology, University Hospital Heidelberg, Heidelberg, Germany; 3 DZHK (German Centre for Cardiovascular Research), partner site Heidelberg/Mannheim, Heidelberg, Germany

**Keywords:** Physiology, Molecular biology

## Abstract

Cellular protein homeostasis, or proteostasis, underpins the integrity, adaptability, and survival of all cells by balancing protein synthesis, folding, trafficking, and degradation. This multilayered network is sustained by coordinated actions of molecular chaperones, the ubiquitin‒proteasome system, autophagy-lysosomal pathways, and organelle-specific quality control programs. When this equilibrium collapses, misfolded, aggregated, or damaged proteins accumulate, driving organelle dysfunction, maladaptive stress signaling, and disease progression. Disruption of proteostasis is now recognized as a unifying pathological hallmark linking neurodegenerative disorders, cancer, cardiovascular and metabolic diseases, and autoimmune conditions. This is particularly consequential in post-mitotic organs such as the heart and brain, which possess limited regenerative capacity and are exceptionally vulnerable to proteotoxic stress. Rapid advances now reveal proteostasis as a multicomponent, cross-compartmental, and dynamically adaptable system, rather than isolated pathways. We frame this complexity through the concept of proteostasis resilience, defined as the ability of cells and tissues to maintain proteome stability under stress, and use it to unify disease mechanisms with therapeutic opportunity. This review integrates mechanistic insights with translational advances, outlining how dysregulation of chaperones, autophagy-mitophagy, the ubiquitin‒proteasome system, and ER stress pathways drive human diseases, while highlighting emerging therapeutic platforms, from pharmacological chaperones and autophagy modulators to targeted protein degradation technologies, CRISPR screens, spatial biology, and AI-guided drug discovery. Together, this systems-level perspective positions proteostasis resilience as a foundational paradigm for understanding disease vulnerability and designing precision proteostasis-based therapies.

## Introduction

**Fig. 1:**
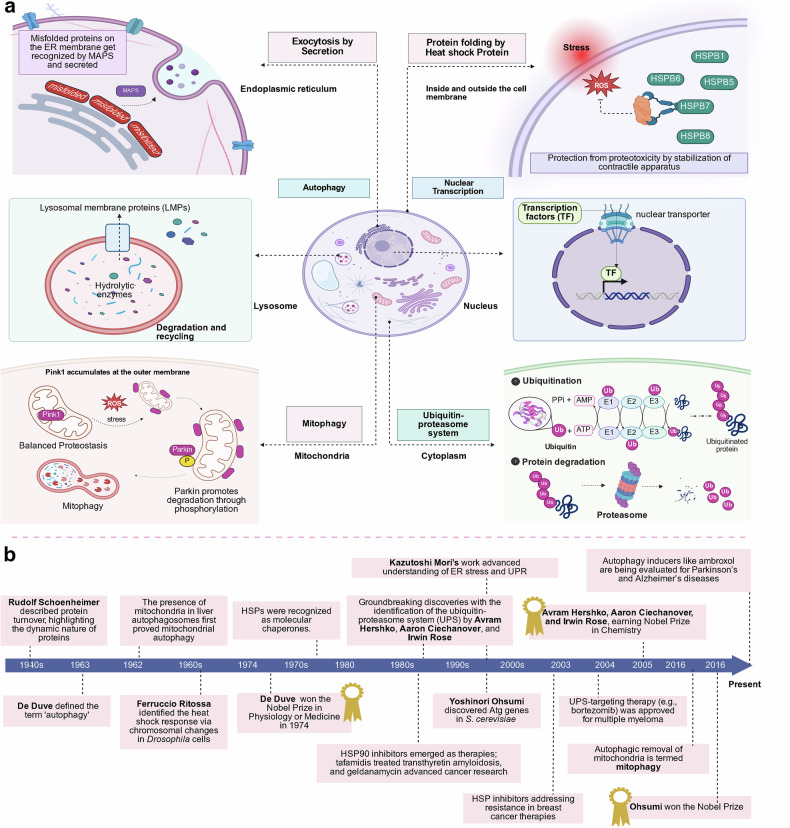
Architecture and historical evolution of cellular proteostasis networks. **a** Cellular proteostasis: a comprehensive overview. This figure illustrates the intricate network of protein quality control mechanisms within cardiomyocytes. It highlights key processes, including the role of autophagy and the ubiquitin proteasome system in removing damaged proteins, the precision of protein folding within the endoplasmic reticulum, and the maintenance of mitochondrial proteostasis. The image provides a snapshot of these essential components, although it represents only a subset of the complex signaling pathways involved. Key components include microtubule-associated proteins (MAPs), PTEN-induced kinase 1 (PINK1), heat shock proteins (HSPs), and reactive oxygen species (ROS). **b** Historical timelines and milestones in proteostasis research. This figure outlines the key historical milestones and breakthroughs in proteostasis research, tracing its evolution from the 1940s to the present. Rudolf Schoenheimer’s concept of protein turnover laid the foundation for understanding protein dynamics. In the 1950s and 1960s, Christian de Duve discovered lysosomes and autophagy, while Ferruccio Ritossa’s work led to the identification of heat shock proteins (HSPs). The 1980s marked a breakthrough with the discovery of the ubiquitin‒proteasome system (UPS) by Hershko, Ciechanover, and Rose, transforming protein degradation research. The 1990s brought advancements in endoplasmic reticulum (ER) stress, unfolded protein response (UPR), and Yoshinori Ohsumi’s identification of autophagy-related genes (Atg), later earning him the 2016 Nobel Prize. Since the 2000s, proteostasis research has driven clinical innovations, including proteasome inhibitors for cancer therapy, HSP inhibitors for drug resistance, and autophagy modulation for neurodegeneration. The emergence of mitophagy further highlighted proteostasis in mitochondrial health. Today, proteostasis dysfunction is recognized as a key factor in metabolic, neurodegenerative, cardiovascular, and autoimmune diseases, underscoring its significance in biomedical research and therapeutic development. This figure was created at Biorender.com

Proteostasis, or protein homeostasis, refers to the intricate balance of processes that regulate protein synthesis, folding, trafficking, and degradation to sustain a functional proteome throughout life. Proteostasis can be treated as a systems-level control problem: cells must continuously maintain a functional proteome despite stochastic translation errors, folding challenges, chemical damage, and fluctuating metabolic demands. Conceptually, the proteostasis network can be divided into two interlocking arms. The *proactive arm* governs proteome production and maturation, translation, co-translational quality control, chaperone-assisted folding, trafficking, and compartment-specific targeting, thereby limiting the generation of metastable or mislocalized proteins (Fig. 1a). The *reactive arm* identifies proteins that escape productive folding or become damaged over time and routes them into clearance or repair pathways, primarily the ubiquitin‒proteasome system and lysosomal autophagy (Fig. 1a). In practice, these arms operate simultaneously: biosynthesis is tuned to folding capacity, and degradation capacity feeds back to biosynthesis through stress signaling and metabolic cues. Failure of these systems results in the accumulation of misfolded or aggregated proteins, organelle dysfunction, cellular injury, and disease progression across a wide spectrum of conditions, including neurodegeneration, cancer, cardiovascular and metabolic diseases. A second defining feature of proteostasis is its multicompartmental architecture. Protein folding and quality control are not uniform across the cell; they are specialized within the cytosol, endoplasmic reticulum, mitochondria, and lysosome, each with distinct folding environments, surveillance machinery, and stress-response programs (Fig. 1a).^1^ This compartmentalization matters because proteotoxic stress rarely remains local. For example, a defect in mitochondrial protein import or respiratory function can amplify cytosolic stress via reactive oxygen species and altered NAD+/ATP balance; conversely, chronic ER stress can reshape cytosolic translation and proteasome load. Thus, “proteostasis failure” is often a network phenomenon: a primary bottleneck in one organelle can propagate into secondary collapse elsewhere.

Foundational insights into proteostasis were laid in the 1940s, when Rudolf Schoenheimer conceptually established the dynamic turnover of body proteins, demonstrating continuous synthesis and degradation (Fig. 1b).^2^ In the following decades, Christian de Duve’s discovery of lysosomes and his subsequent identification of autophagy defined intracellular degradation and recycling pathways as central elements of proteostasis.^3–5^ Parallel breakthroughs occurred in stress biology, beginning with Ferruccio Ritossa’s discovery of heat-induced chromosomal “puffs” in Drosophila, which led to the recognition of the heat shock response and the discovery of heat shock proteins (HSPs).^6–8^ These proteins have emerged as critical molecular chaperones that facilitate folding, prevent aggregation, and stabilize proteins under stress. The discovery of the ubiquitin‒proteasome system (UPS) in the 1980s by Hershko, Ciechanover, and Rose revolutionized our understanding of intracellular protein degradation and enabled the development of proteasome-targeted cancer therapies.^9,10^ Subsequent advances revealed the role of HSPs in oncogenesis and treatment resistance, leading to the development of HSP inhibitors.^11,12^ The 1990s introduced crucial discoveries in endoplasmic reticulum (ER) stress signaling and unfolded protein response (UPR) pathways, establishing ER proteostasis as a cornerstone of metabolic and neurodegenerative disease biology.^13^ Yoshinori Ohsumi’s identification of autophagy-related genes in yeast during the 1990s defined the molecular machinery of autophagy,^14,15^ while later work highlighted selective mitochondrial autophagy (mitophagy) as essential for mitochondrial quality control and cellular survival^16,17^ (Fig. 1a).

Today, proteostasis disruption is recognized as a shared pathological denominator across neurodegenerative disorders, cancer, cardiovascular diseases, metabolic syndromes, and autoimmune conditions. The inability to appropriately sense, buffer, or correct proteotoxic stress results in the accumulation of misfolded proteins, organelle dysfunction, aberrant signaling, and ultimately tissue failure. These pathways not only preserve proteome integrity but also offer powerful therapeutic opportunities, as demonstrated by the emergence of proteasome inhibitors, autophagy modulators, and molecular chaperone-targeting agents in clinical medicine (Tables 1 and 2). Despite rapid advances, the current understanding of proteostasis remains fragmented, with most studies historically examining individual pathways or organ systems in isolation. However, it is increasingly evident that proteostasis is not governed by discrete modules but by a highly interconnected network characterized by cross-compartment communication, signaling integration, and adaptive compensation. Moreover, the concept of ‘proteostasis resilience, the capacity of cells and tissues to preserve proteome integrity under sustained stress’, is emerging as a powerful unifying lens to understand disease vulnerability, progression, and therapeutic responsiveness.

**Table 1:** List of drugs targeting protein homeostasis

Drug name	Target	Disease	Pathway	Benefits	Disadvantages
Bortezomib	Proteasome (20S)	Multiple Myeloma, Lymphoma	Ubiquitin-Proteasome System	Effective in reducing proteasome activity, inducing apoptosis	Peripheral neuropathy, resistance development
Carfilzomib	Proteasome (20S/19S)	Multiple Myeloma	Ubiquitin-Proteasome System	Greater specificity, reduced off-target effects	Cardiotoxicity, infusion reactions
Hydroxychloroquine	Autophagy inhibition	Lupus, Rheumatoid Arthritis	Lysosomal-autophagy pathway	Reduces lysosomal degradation, anti-inflammatory effects	Retinopathy, cardiotoxicity
Ixazomib	Proteasome (oral agent)	Multiple Myeloma	Ubiquitin-Proteasome System	Oral bioavailability, improved compliance	Gastrointestinal toxicity
Rapamycin	mTOR inhibition	Organ Transplant, Cancer	Autophagy	Promotes autophagy, inhibits proliferation	Hyperlipidemia, impaired wound healing
Everolimus	mTOR inhibition	Renal Cell Carcinoma, TSC	Autophagy	Anti-proliferative, promotes autophagy	Hyperglycemia, immunosuppression
Thalidomide	CRBN modulation	Multiple Myeloma, Leprosy	Ubiquitin-Proteasome System	Protein degradation via CRBN, immune modulation	Teratogenicity, neuropathy
Venetoclax	BCL-2 inhibition	Chronic Lymphocytic Leukemia	Apoptosis	Selective apoptosis induction	Resistance development, neutropenia
Cetuximab	EGFR inhibition	Colorectal Cancer, Head & Neck Cancer	Protein Folding	Targets misfolded EGFR signaling in cancer	Resistance, infusion reactions
Atezolizumab	PD-L1 blockade	Lung Cancer, Breast Cancer	Immune Checkpoints/Proteostasis	Stimulates immune responses	Immune-related adverse events
Marizomib	Proteasome inhibition	Glioblastom, Multiple Myeloma	Ubiquitin-Proteasome System	Broad-spectrum proteasome inhibition, CNS penetrance	Neurotoxicity, limited efficacy
Tanespimycin	HSP90 inhibition	Multiple Cancers	Heat Shock Proteins	Disrupts oncogenic protein folding	Hepatotoxicity
BGP15	HSP 70 induction	Alzheimers, Parkinsons, Ischemia, Metabolic Disorders	Heat Shock Proteins	Neuro protective, cardioprotective effects	Not yet studied long term, increased cellular stress
Arimoclomol	HSP70 enhancement	ALS, Niemann-Pick Disease	Heat Shock Proteins	Enhances cellular stress responses	In clinical trials
Elamipretide	Mitochondrial stabilization	Mitochondrial Myopathies	Mitophagy	Improves ATP production, stabilizes mitochondria	Limited clinical efficacy data
Mitapivat	Pyruvate kinase activation	Pyruvate Kinase Deficiency	Mitochondrial Proteostasis	Enhances energy metabolism	Data unavailable
Cyclosporin A	Calcineurin inhibition	Autoimmune Diseases, Transplants	Autophagy, Protein Folding	Reduces proteotoxicity in stressed cells	Nephrotoxicity, hypertension
SD36 (PROTAC)	STAT3 degradation	Cancer	Targeted Protein Degradation	Selectively degrades oncogenic STAT3 protein	Experimental, newly discovered
ARV-110	Androgen receptor	Prostate Cancer	Targeted Protein Degradation	Overcomes resistance in AR signaling	Limited data
ARV-471	Estrogen receptor	Breast Cancer	Targeted Protein Degradation	ER degradation in resistant tumors	Limited data
LYTAC (Experimental)	Extracellular proteins	Cancer, Autoimmune	Lysosomal targeting	Targets extracellular proteins	Preclinical
AbTAC (Experimental)	Cell surface proteins	Oncology	Antibody-induced degradation	Specific targeting via antibodies	Preclinical
Geldanamycin	HSP90 inhibition	Cancer	Heat Shock Proteins	Reduces tumor growth by destabilizing protein folding	Toxicity concerns
Chloroquine	Lysosomal inhibition	Malaria, Autoimmune Diseases	Autophagy	Blocks autophagosome degradation	Retinopathy
Lenalidomide	CRBN	Multiple Myeloma	Ubiquitin-Proteasome System	CRBN modulation for targeted protein degradation	Resistance, off-target effects
Ganetespib	Heat Shock Protein 90 (Hsp90)	Lung, Breast Cancer	Heat Shock Protein Inhibition	Stabilizes oncogenic proteins, aiding in cancer cell death; promising in preclinical and clinical trials	Possible toxicity and inadequate efficacy in some patients
Luminespib	Heat Shock Protein 90 (Hsp90)	Brest Cancer, Non-small Cell Lung Cancer	Heat Shock Protein Inhibition	Blocking ATP hydrolysis, AUY922 inhibits the chaperone cycle	Ocular toxicity, Hepatotoxicity, Gastrointestinal side effects
Solanezumab	Amyloid-beta Aggregates	Alzheimer’s Disease	Protein aggregates	Targets soluble amyloid-beta monomers, delays cognitive decline in some patients	No significant cognitive benefit seen in clinical trials
Xenazine	Protein aggregates	Huntington’s Disease	Protein Aggregates (Huntingtin)	Reduces dopaminergic neurotoxicity and protein aggregation	Side effects like depression and akathisia
Tadalafil	Heat shock proteins	Pulmonary Hypertension	cGMP Pathway, Heat Shock Proteins	Increases cGMP levels, affecting protein folding and stress response in cardiovascular conditions	Limited by cardiovascular side effects
Fenofibrate	Peroxisome Proliferator-Activated Receptor Alpha (PPAR-α), Autophagy	Type 2 Diabetes	Autophagy	Enhances autophagy and protein degradation, aiding glycemic control	Gastrointestinal and liver toxicity in some patients
Tocilizumab	Heat Shock Proteins, IL-6 Pathway	Rheumatoid Arthritis, Systemic Juvenile Idiopathic Arthritis	Heat shock proteins	Reduces systemic inflammation; modulates protein homeostasis	No effect on protein aggregation in synovial fluid; delayed onset
Nitisinone	Ubiquitin-Proteasome System	Alkaptonuria	Ubiquitin-Proteasome System	Reduces accumulation of homogentisic acid; protects cartilage	High cost; limited to specific metabolic disorder
Liraglutide	Protein Aggregation, Autophagy	Obesity-induced HFpEF, Type 2 Diabetes	Protein Aggregation, Autophagy	Improves protein turnover and glucose control; impacts neurodegenerative proteins	Gastrointestinal issues, hypoglycemia
Dapagliflozin	Mitophagy, Protein Aggregation	Obesity-related HFpEF, Diabetes	Mitophagy, Protein Aggregation	Enhances mitochondrial function; reduces protein aggregation	Limited clinical data- can lead to dehydration, UTI, hypoglycemia
Empagliflozin	Protein Degradation	Diabetes, Cardiovascular Diseases	Protein Degradation, Mitochondrial Function	Improves heart failure outcomes; reduces myocardial infarction risk	Potential increase in heart failure hospitalizations; risk of dehydration
Ambroxol	Lysosomal pathway	Parkinson’s Disease, Neurodegeneration	Lysosomal Function, Protein Aggregates	Enhances lysosomal function; reduces alpha-synuclein aggregation	Mixed clinical results; potential side effects
Gantenerumab	Protein aggregates	Alzheimer’s Disease	Protein aggregates and plaque improvement	Improves plaques Alzheimer’s disease	Limited efficacy in advanced stages of Alzheimers disease.
Sorafenib	Autophagy	Renal Cell Carcinoma	Autophagy modulation	Induces autophagy to reduce misfolded proteins	Induces cellular stress
Lumacaftor	Protein folding	Cystic Fibrosis	Protein folding	Enhances folding of protein	Cellular damage due to aggregate formation s the mode of action is not potent enough
Bapineuzumab	Protein aggregates	Neurodegeneration	Protein aggregates	Clearing of amyloid-beta plaques in the brain	Brain swelling and microhemorrhages
Riluzole	Protein aggregate	Amyotrophic Lateral Sclerosis	Induction of protein aggregates	Inducing protein aggregate formation for protein clearance	Liver toxicity, nausea
Spermidine	Autophagy via eIF5A modification	Cadiovascular, Neurodenerative Diseases	Induced autophagy resulting in clearance of protein accumulation	Improved mitochondrial health and oxidative stress, reduction in amyloid-beta and tau accumulation	Spermidine can enhance cell proliferation, which may promote tumor growth in cancers
Geranylgeranylacetone (GGA)	HSP inducer	Cardiovascular, Neurodegenerative, Liver Diseases	Activation of heat shock transcription factor 1 (HSF1), leading to activation of HSP70 and HSP90	Cytoprotection, cardioprotective, anti inflammatory	HSP induction could inadvertently protect cancer cells, might disrupt cellular homeostasis
Urolithin A	Mitophagy inducer	Neurodegenrative, Skeletal Muscle, Cardiovascular Diseases	Acts via the AMPK-PGC-1α pathway and promotes mitochondrial biogenesis	Anti inflammatory, increases mitophagic flux	Dependance on gut microbiome, gastrointestinal discomfort

This table lists drugs that modulate protein homeostasis, a critical process for maintaining cellular proteome balance. The table includes drug names, their mechanisms of action, and their primary molecular targets. These drugs are being investigated for their potential therapeutic effects in various diseases, including cancer, neurodegenerative disorders, cardiovascular diseases, metabolic syndromes, and autoimmune conditions

**Table 2:** List of ongoing or completed clinical trials targeting protein homeostasis

Drug name	Target pathway	Disease condition	Clinical trial number	Trial phase	Information
Gantenerumab	Amyloid-beta Aggregates	Alzheimer’s Disease	NCT02051608	Phase III	Antibody targeting amyloid plaques have varied outcomes, but trials for Alzheimer’s and mild cognitive impairment have shown improvement.
PROTAC-based ARV-110	Ubiquitin-Proteasome System	Metastatic castration-resistant prostate Cancer (mCRPC)	NCT03888612	Phase I/II	Targets androgen receptor for degradation; promising results in resistant prostate cancer.
PROTAC-based ARV-471	Ubiquitin-Proteasome System	ER+ Breast Cancer	NCT04072952	Phase I/II	Demonstrated safety and efficacy in ER+ cancers resistant to standard endocrine therapy.
Atezolizumab	Autophagy/Lysosomal Pathway	Lung Cancer, Bladder Cancer	NCT02366143	Phase II/III	Inhibits PD-L1 and affects tumor-associated immune autophagy; enhances immune clearance of aggregates.
Rapamycin	Autophagy Induction	Age-related Neurodegeneration	NCT04629495	Phase II	mTOR inhibitor inducing autophagy; delays protein aggregate accumulation in diseases like Alzheimer’s.
Cetuximab	Lysosomal Degradation	Head and Neck Squamous Cell Carcinoma	NCT00088907	Phase III	EGFR inhibitor; enhances lysosome-mediated degradation of aggregated proteins.
LYTACs (Lysosome-Targeting Chimeras)	Lysosomal Pathways	Exploratory (Preclinical Cancer Models)	N/A	Preclinical	Explores lysosomal degradation of extracellular and membrane proteins; potential in oncology.
Bortezomib	Proteasome Inhibition	Multiple Myeloma, Mantle Cell Lymphoma	NCT00522392	Phase III	Inhibits 26S proteasome, leading to accumulation of misfolded proteins and cell death in tumor cells.
ATTECs (Autophagy-Tethering Compounds)	Autophagy	Neurodegenerative Disorders (Preclinical)	N/A	Preclinical	Induces degradation of disease-associated proteins like mutant Huntingtin (mHTT) via LC3 tethering.
VER-155008	Hsp70 Inhibition	Cancer	Preclinical	Preclinical	Hsp70 inhibitor showing potential in disrupting protein-folding networks crucial for cancer cell survival.
Ganetespib	Hsp90 Inhibition	Lung and Breast Cancer	NCT01348126	Phase II	Destabilizes Hsp90 client proteins critical for tumor growth and metastasis.
Sorafenib	Autophagy Modulation	Liver Cancer, Renal Cell Carcinoma	NCT00492752	Phase III	Multi-kinase inhibitor; modulates autophagy, potentially counteracting therapy resistance.
Chaperone-Modulating Drugs (e.g., Lumacaftor)	Protein Folding	Cystic Fibrosis	NCT01807923	Phase III	Promotes proper folding and trafficking of mutant CFTR protein (ΔF508 mutation).
CAR-T Therapies Targeting Protein Degradation	Ubiquitin-Proteasome and Aggregates	Various Cancers	NCT05779917	Various	Utilizes engineered T cells to target specific aggregated or misfolded proteins in cancer cells.
Bapineuzumab	Amyloid-beta Aggregates	Alzheimer’s Disease,	NCT00574132,	Phase III	Monoclonal antibody targeting amyloid plaques; trials failed to show cognitive benefit despite amyloid clearance.
IVIG (Intravenous Immunoglobulin)	Protein Aggregates (Various, including amyloid)	Alzheimer’s Disease, Autoimmune Diseases	NCT00382343, NCT01300728	Phase III	Targets aggregates by immune modulation; potential in neurodegeneration and autoimmune diseases.
Lecanemab	Amyloid-beta Aggregates	Alzheimer’s Disease	NCT03887455	Phase III	Monoclonal antibody targeting amyloid-beta oligomers; recently showed efficacy in clinical trials.
Gantenerumab	Amyloid-beta Aggregates	Alzheimer’s Disease	NCT02051608	Phase III	Antibody targeting amyloid plaques; results are mixed, but still progressing in trials for Alzheimer’s and mild cognitive impairment.
Ambroxol	Lysosomal Function, Protein Aggregates	Parkinson’s Disease, Neurodegeneration	NCT02941822	Phase II	Enhances lysosomal function; in trials for Parkinson’s, aiming to reduce alpha-synuclein aggregation.
Arimoclomol	Hsp70 Induction, Protein Aggregates	ALS, Parkinson’s Disease	NCT00561366	Phase II	Small molecule that induces Hsp70 to assist in protein refolding and prevent aggregation in neurodegenerative diseases.
Tauroursodeoxycholic Acid (TUDCA)	Protein Aggregates, Autophagy	Huntington’s Disease, ulcerative colitis	NCT00514774, NCT04114292	Phase II	Promotes autophagy and reduces protein aggregation; shown to improve cellular stress responses in neurodegeneration.
Cytosorb	Protein Aggregates (Inflammation)	Sepsis, Organ Dysfunction	NCT02919839	Phase II	Hemoadsorption system targeting pro-inflammatory cytokines and protein aggregates; used in critical care and organ dysfunction.
NPT088	Protein Aggregates (Neurodegeneration)	Alzheimer’s Disease, Parkinson’s Disease	NCT03008161	Phase I/II	Monoclonal antibody targeting tau aggregates; early-stage clinical trials in neurodegenerative conditions.
Colchicine	Ubiquitin-proteasome system, autophagy, and heat shock proteins	Amyotrophic Lateral Sclerosis (ALS)	NCT03693781	Phase II	The trial evaluates two doses of colchicine (0.01 mg/kg/day and 0.005 mg/kg/day) against a placebo. Colchicine enhances expression of HSPB8 and autophagy mediators, addressing TDP-43 protein aggregation—a hallmark of ALS.
Riluzole	Protein Aggregates, Glutamate Toxicity	ALS (Amyotrophic Lateral Sclerosis)	NCT00542412	Phase IV	FDA-approved drug for ALS; inhibits glutamate release and reduces neurotoxic protein aggregation.
Patisiran	Protein Aggregates (TTR amyloidosis)	Transthyretin Amyloidosis	NCT01960348	Phase III	RNA interference therapy targeting TTR gene to prevent amyloid aggregation; FDA-approved for TTR amyloidosis.
Eteplirsen	Protein Aggregates, Protein Folding	Duchenne Muscular Dystrophy	NCT01396239	Phase III	Antisense oligonucleotide promoting skipping of exon 51 in dystrophin gene to improve protein function.
Cladribine	Protein Aggregates (Immune Cells)	Multiple Sclerosis	NCT00725985	Phase III	Chemotherapy drug targeting immune cells; alters protein homeostasis in MS treatment.
BGP-15	Chaperone function, Aggregation of misfolded proteins.	Type 2 Diabetes	NCT01069965	Phase II	Investigated as an insulin sensitizer for improving glucose metabolism.
BGP-15	Enhances autophagic flux	Dilated Cardiomyopathy	NCT01981212	Phase II	Examined for heart protection and improving heart failure symptoms.
BGP-15	Modulating Autophagy	COVID-19	2020-005539-62	Phase III	Tested for potential efficacy in treating COVID-19 (Terminated).
BGP-15	Stabilizes lysosomal membranes	Sinus Tachycardia	2020-003005-61	Phase II	Studied for its effects on heart rhythm disturbances (Active, not recruiting).
BGP-15	Enhancing Mitophagy	Insulin Resistance & Obesity	BGP-15-CLIN-IR04	Phase II	Focused on its role in managing insulin resistance (Terminated).
Tafamidis	Protein Aggregation	Transthyretin Amyloidosis	NCT01994889	Phase III	Stabilizes transthyretin tetramers, reducing amyloid fibril formation and associated cardiomyopathy.
Vorasidenib	Ubiquitin-Proteasome System	Gliomas with IDH mutations	NCT04164901	Phase III	Affects mutant IDH1/2 proteins by altering proteostasis and energy metabolism pathways.
BIIB122	Autophagy	Parkinson’s Disease	NCT06602193	Phase II	Modulates LRRK2, improving lysosomal function and clearing aggregated α-synuclein.
Omaveloxolone	Mitophagy	Friedreich’s Ataxia	NCT02255435	Phase II/III	Induces Nrf2, which promotes mitochondrial repair and reduces oxidative stress.
Lenalidomide	E3 Ubiquitin Ligase	Multiple Myeloma	NCT00480363	Phase III	Targets cereblon E3 ligase, altering protein degradation and immune modulation.
Liraglutide	Heat Shock Proteins	Non-alcoholic Steatohepatitis (NASH)	NCT01237119	Phase II	Linked to improved hepatic stress responses through modulation of Hsp70 and Hsp90 pathways.
Dapagliflozin	Autophagy	Heart Failure with Preserved EF	NCT03036124	Phase III	Enhances cardiomyocyte autophagy, reducing stress-induced protein aggregation.
Cenicriviroc	Lysosomal System	Fibrosis in NASH	NCT04321031	Phase IIb	Targets lysosomal degradation of fibrotic markers by inhibiting CCR2/CCR5.
ARV-110	Proteasomal Degradation	Metastatic Castration-Resistant Prostate Cancer	NCT03888612	Phase II	PROTAC that hijacks E3 ligases to degrade androgen receptor proteins.
Verubecestat	Amyloid Processing	Alzheimer’s Disease	NCT01739348	Phase III	Investigates BACE1 inhibition to reduce amyloid plaques. Trial terminated due to lack of efficacy.
Carfilzomib	Proteasome Inhibition	Multiple Myeloma	NCT01302392	Phase III	Irreversibly inhibits the 20S proteasome, disrupting protein degradation in cancer cells.
BIIB080	Tau Protein Aggregation	Alzheimer’s Disease	NCT05399888	Phase I	Antisense oligonucleotide therapy targeting tau mRNA to reduce tau protein accumulation.
PTC299	Ubiquitin Proteasome	Acute Myeloid Leukemia (AML)	NCT03761069	Phase II	Inhibits DHODH to affect nucleotide metabolism and proteasomal degradation in AML cells.
Arimoclomol	Heat Shock Proteins	Niemann-Pick Disease Type C	NCT02612129	Phase II/III	Enhances cellular stress response by modulating heat shock proteins, facilitating the breakdown of accumulated lysosomal substrates.
Verubecestat	Beta-secretase Inhibition	Alzheimer’s Disease	NCT01739348	Phase II/III	Focused on reducing beta-amyloid aggregates, though trials have reported limited efficacy and adverse cognitive outcomes.
Nilotinib	Lysosomal Degradation	Parkinson’s Disease	NCT02954978	Phase II	Evaluated for repurposing to clear protein aggregates by inducing autophagy.
Spermindine	Autophagy	Cardiovascular and Neurodegenerative Disease	NCT04405388	Phase IIb	Investigates spermidine’s impact on memory and cognitive performance and its role as a biomarker.

This table summarizes clinical trials investigating drugs that target protein homeostasis in different disease contexts, including cardiovascular, metabolic, cancer, neurodegenerative, and autoimmune diseases. The table includes details on the drug name, target mechanism, disease indication, clinical trial phase, and trial status, providing an overview of ongoing efforts to harness protein homeostasis as a therapeutic strategy

## Proteostasis as an integrated multilayer network: a conceptual framework

Proteostasis is sustained by a hierarchically organized and tightly interconnected network of processes that collectively determine whether proteins remain functional, are repaired or are eliminated. Conceptually, this network can be viewed through three interlinked dimensions:

- Functional dimension: proactive vs. reactive proteostasis.
- Spatial dimension: compartment-specific regulation.
- Systems dimension: resilience, crosstalk, and compensation.

Together, these dimensions define how cells sense proteotoxic stress, allocate corrective resources, and maintain proteome stability under physiological and pathological conditions (Fig. 1a).

### Proactive vs. reactive proteostasis: a functional continuum

Rather than acting as isolated modules, proteostasis pathways operate along a functional continuum that spans proactive maintenance and reactive stress responses. Under physiological conditions, *proactive proteostasis mechanisms* establish and preserve baseline proteome quality. These include ribosome-associated quality control during protein synthesis, molecular chaperone systems such as heat shock proteins that ensure proper folding and conformational stability and intracellular trafficking pathways that guide nascent proteins to their correct destinations. These proactive systems minimize misfolding and maintain proteome efficiency during normal physiology, but they are energetically demanding and become insufficient when cells encounter substantial proteotoxic stress.

When the burden of misfolded or damaged proteins exceeds preventive capacity, *reactive proteostasis machinery* is engaged to restore equilibrium. The UPS selectively degrades misfolded or damaged soluble proteins, whereas macroautophagy and selective autophagy pathways eliminate protein aggregates and dysfunctional organelles via lysosomal degradation. Within the endoplasmic reticulum, the ER-associated degradation (ERAD) pathway and the UPR collectively detect folding stress, reduce protein load, and enhance repair capacity. In parallel, mitochondrial proteases, chaperones, and mitophagy maintain mitochondrial proteome integrity, which is critical for cellular energetics and survival. These mechanisms do not act sequentially or independently; instead, they form a dynamic and adaptive response network in which impairment of one branch often triggers compensatory activation of another. This coordinated flexibility underlies the cell’s ability to preserve proteome stability.

### The spatial dimension: compartmental proteostasis

Proteostasis is also fundamentally *spatially organized*. Each major cellular compartment possesses distinct protein quality control strategies tailored to its function and biochemical environment. Cytosolic proteostasis relies heavily on chaperones, sequestration systems, and UPS-mediated degradation. The ER integrates folding quality control with stress-sensing machinery to regulate cellular proteome load, while mitochondria require dedicated surveillance systems to maintain bioenergetic competence. Lysosomes coordinate final-stage degradation via autophagy, clearing aggregates and damaged organelles to prevent persistent proteotoxic stress. Communication between compartments ensures coordination; for example, ER stress influences autophagy activation, mitochondrial dysfunction recruits mitophagy, and proteasomal overload promotes autophagic compensation. These cross-compartment feedback loops are central to maintaining global proteome balance.

### Proteostasis resilience: a systems-level perspective

Together, these functional, spatial and adaptive properties define what can be conceptualized as proteostasis resilience, the capacity of cells, tissues and organisms to sustain proteome integrity despite acute or chronic stress. Resilience is determined by the robustness of individual pathways, the efficiency of stress sensing and signaling coordination, and the ability of parallel systems to compensate when one mechanism becomes overwhelmed. It is highly context-dependent: organs such as the heart and brain, with high metabolic demand and limited regenerative capacity, are particularly vulnerable to resilience failure, whereas cancer cells exploit enhanced proteostasis resilience to support uncontrolled proliferation and therapy resistance. Loss of this resilience, whether due to genetic mutations, aging, metabolic overload, chronic inflammation, or environmental insults, marks a tipping point toward disease vulnerability and progression.

In the sections that follow, we build on this conceptual framework to examine how major proteostasis machineries contribute to cellular and organ-level resilience and how their failure drives pathology across cardiovascular, cancer, metabolic, neurodegenerative and autoimmune disorders and highlight therapeutic strategies aimed at reinforcing proteostasis resilience. We further discuss emerging advances in omics, spatial biology, genome editing and artificial intelligence that enable deeper mapping and predictive modeling of proteostasis networks, paving the way toward personalized, mechanism-based interventions. Through this integrative approach, we aim to bridge mechanistic understanding with clinical translation and to position proteostasis resilience as a conceptual foundation for future research and therapeutic innovation.

## Proteostasis in cardiovascular health and disease

Cardiovascular tissues, particularly the heart, operate under constant mechanical, metabolic and oxidative stress and therefore depend critically on robust proteostasis to sustain contractile function, bioenergetic efficiency and cellular survival. Cardiomyocytes are terminally differentiated and have limited regenerative capacity; thus, the accumulation of misfolded proteins, dysfunctional organelles, or irreversible aggregates has disproportionately severe consequences. Disruption of proteostasis is now recognized as a core pathological mechanism across cardiomyopathies, ischemic heart disease, arrhythmias, and heart failure, linking molecular protein quality control failure to tissue remodeling and clinical disease progression.

### The role of heat shock proteins in the heart

#### Basal and stress-induced expression of HSPs in cardiomyocytes

**Fig. 2:**
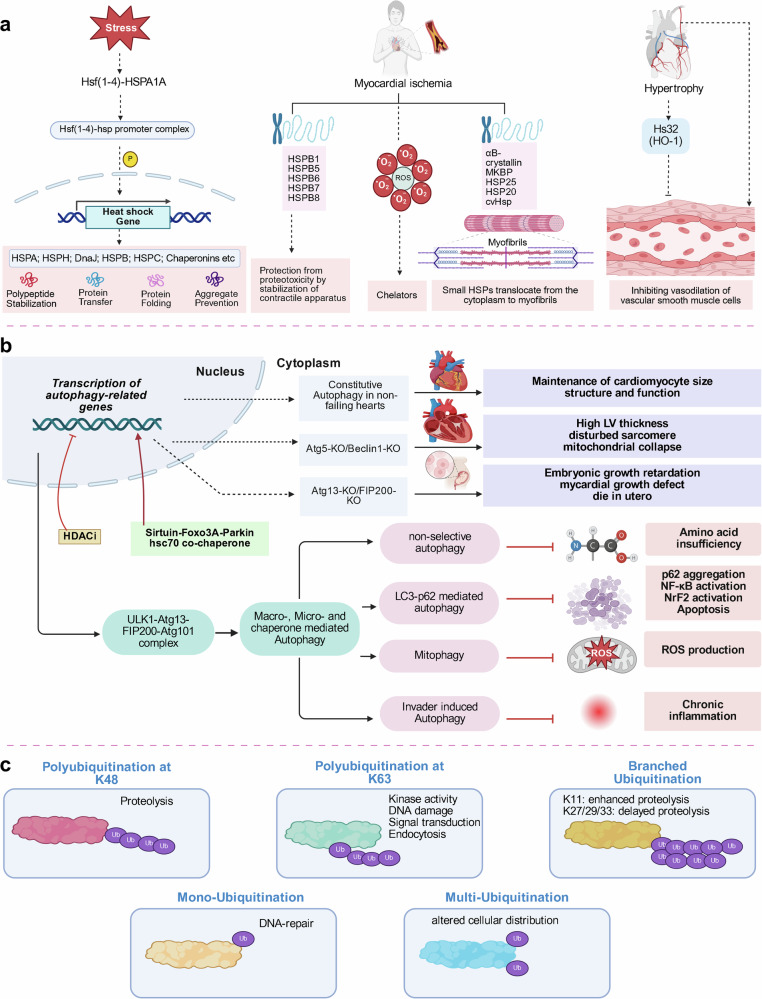
Proteostasis control mechanisms in the heart: roles of heat shock proteins, autophagy, and ubiquitination. **a** Role of heat shock proteins (HSPs) in the heart. This figure depicts the involvement of heat shock proteins (HSPs) in cardiac function and stress responses. It shows the interactions between HSPs, heat shock factors (Hsfs), and other associated proteins, such as MKBP (myotonic dystrophy protein kinase binding protein) and cvHSPs (cardiovascular heat shock proteins). The diagram underscores the critical role of HSPs in protecting cardiomyocytes under stress conditions. **b** A comprehensive overview of cellular self-eating machinery. This figure provides a detailed exploration of the autophagy process, including its regulatory pathways and mechanisms crucial for maintaining cellular homeostasis, with a focus on cardiac diseases. It features autophagy-related genes and proteins, such as HDACi (Histone Deacetylase Inhibitors), Atg (Autophagy Genes), ULK1 (Unc-51 Like Kinase 1), FIP200 (Focal Adhesion Kinase Family Interacting Protein of 200 kD), LC3 (Microtubule-Associated Proteins 1A/1B Light Chain 3B), NFκB (Nuclear Factor κ-light-chain-enhancer of Activated B-cells), and NRF2 (Nuclear Factor Erythroid-2 Related Factor 2). **c** Schematic overview of ubiquitination in the cellular regulation of protein degradation. This schematic highlights the role of ubiquitination in protein degradation and other cellular processes. While ubiquitination is commonly associated with the proteasome complex, it also plays a crucial role in various signaling pathways, including DNA repair and transcription. The figure illustrates the broad impact of ubiquitin (Ub) beyond its traditional role in proteasomal degradation. This figure was created at Biorender.com

Cardiomyocytes exhibit intrinsically high expression levels of HSPs, reflecting the heart’s continuous exposure to mechanical load, metabolic demand, and oxidative stress. Among the HSP families, small heat shock proteins (sHSPs), also referred to as HSPB proteins, are particularly abundant in cardiac tissue (Fig. 2a). These proteins play essential roles in maintaining cytoskeletal integrity and promoting cardiomyocyte survival under both basal and stress conditions. During myocardial ischemia, arising from pathological events such as unstable atherosclerotic plaque rupture, coronary occlusion, or congestive heart failure, the expression of several sHSPs is markedly upregulated. These include HSPB1 (HSP27), HSPB5 (αB-crystallin), HSPB6 (HSP20), HSPB7 (cardiovascular HSP), and HSPB8 (HSP22). Induction of these chaperones represents an adaptive stress response that protects cardiomyocytes by stabilizing the contractile apparatus and preserving sarcomeric organization during ischemic injury.^18^ The coordinated upregulation of sHSPs underscores their central role in sustaining myocardial structural integrity under acute and chronic stress.

#### HSP-mediated protection against oxidative stress and ischemic injury

In addition to their structural roles, HSPs are integral components of the cardiac defense system against oxidative stress. Myocardial ischemia and reperfusion are accompanied by excessive production of reactive oxygen species (ROS), which contribute to ventricular dysfunction, arrhythmogenesis, and cardiomyocyte death. HSPs mitigate these deleterious effects by functioning as part of a broader free radical scavenger network, thereby reducing intracellular ROS accumulation and limiting oxidative damage (Fig. 1a). HSPs act synergistically with classical antioxidant enzymes, including superoxide dismutase and glutathione peroxidase, to enhance cardioprotection. This cooperative interaction amplifies the heart’s capacity to neutralize oxidative insults and preserve cellular homeostasis. Notably, HSP70 has been shown to interact with catalase-associated pathways, further augmenting antioxidant defenses and improving cardiac function following ischemic injury.^19^ Through these mechanisms, HSPs not only prevent protein misfolding and aggregation but also actively modulate redox balance, reinforcing their multifaceted role in cardiomyocyte survival.

#### αB-Crystallin as a structural and disease-relevant cardiac chaperone

Among cardiac HSPs, HSPB5 (αB-crystallin) has emerged as a particularly critical and disease-relevant chaperone. This ~20 kDa protein exhibits highly specific subcellular localization within cardiomyocytes, concentrating at the Z-band where it associates with desmin intermediate filaments. Under stress conditions, such as heat shock or ischemia, αB-crystallin binds to actin filaments, preventing their aggregation and thereby preserving cytoskeletal integrity and contractile function (Fig. 2a).^20^ The pathological significance of αB-crystallin is underscored by genetic evidence. A missense mutation (R120G) in αB-crystallin leads to abnormal aggregation of both desmin and αB-crystallin, resulting in severe cardiomyopathy known as desminopathy. This condition is characterized by disrupted cytoskeletal architecture and progressive cardiac dysfunction.^21^ Beyond its role in inherited disease, αB-crystallin also contributes to myocardial mechanics in acquired heart failure. In failing human myocardium, αB-crystallin has been shown to reduce elevated cardiomyocyte stiffness, potentially by limiting titin aggregation and improving sarcomeric compliance.^22^

Collectively, these findings establish HSPs, particularly members of the sHSP family, as central determinants of cardiac proteostasis, structural stability, and stress resilience. By stabilizing cytoskeletal elements, preventing protein aggregation, mitigating oxidative stress, and preserving the mechanical properties of cardiomyocytes, HSPs provide robust protection against ischemic and oxidative injury. The stress-induced upregulation of HSPs during myocardial ischemia highlights their adaptive significance, while disease-associated mutations underscore their pathological relevance. A deeper understanding of HSP regulation and function in the heart offers promising opportunities for the development of targeted therapeutic strategies in cardiovascular diseases.

### The role of autophagy and mitophagy in the heart

Given the unique physiology and limited regenerative capacity of cardiomyocytes, autophagy and its mitochondria-selective subtype, mitophagy, collectively emerge as two of the most vital mechanisms for maintaining cardiac proteostasis and long-term myocardial health.

#### Basal autophagy as a determinant of cardiomyocyte integrity and longevity

In the absence of effective autophagy, cellular waste and misfolded proteins accumulate, generating proteotoxic stress, structural disorganization, and organelle dysfunction. Cardiomyocytes, which lack meaningful regenerative potential, are particularly vulnerable to the cumulative effects of aging and chronic stress. Studies using cardiac-specific Atg5 deletion models have been pivotal in establishing this concept (Fig. 2b). Although these mice initially exhibit normal cardiac function, they progressively develop left ventricular dilation, reduced fractional shortening, disorganized sarcomeres, and damaged mitochondria with aging, highlighting the essential role of sustained autophagy in maintaining structural integrity and contractile function over time.^23^ The importance of autophagy extends to cardiac development, as evidenced by embryonic lethality and myocardial developmental defects in Atg13-knockout mice, underscoring the indispensable roles of Atg13 and FIP200 in both autophagy and heart formation (Fig. 2b).^24^

#### Stress-induced autophagy and its dual role in cardiac remodeling

Autophagy is dynamically activated in response to hemodynamic stress. In transverse aortic constriction models, autophagic activity rises rapidly and remains elevated during hypertrophic remodeling. Modulating autophagic regulators profoundly influences these outcomes. Reduction of Beclin1 impairs adaptive remodeling, whereas excessive Beclin1 promotes autophagy beyond beneficial levels and exacerbates adverse remodeling.^25^ Thus, while autophagy is clearly protective under physiological and early stress conditions, its chronic hyperactivation may become maladaptive. Overall, constitutive autophagy maintains cardiomyocyte size, architecture, and basal performance in the healthy heart, whereas in failing myocardium, enhanced autophagy functions predominantly as a compensatory survival mechanism.^26^ This dualistic behavior reflects the complexity and context-dependence of autophagy in cardiac physiology.

#### Molecular and epigenetic modulators of cardiac autophagic flux

Several molecular regulators refine this autophagic network. FYCO1 is an emerging mediator that interacts with LC3, Rab7, and phosphatidylinositol-3-phosphate to regulate autophagosome trafficking and maturation.^27^ Its overexpression increases autophagic flux, promotes the formation of autophagosomes even under nutrient-rich conditions, and improves contractile adaptation under stress, partly by modulating Myh7 expression.^28^ Epigenetic regulators also influence autophagy. Histone deacetylases (HDACs) integrate stress-responsive signaling with autophagic activation; HDAC inhibition modulates autophagy, limits phenylephrine-induced hypertrophy, and preserves near-normal cardiac function in pre-hypertrophic models (Fig. 2b).^29^ Collectively, these insights reinforce that autophagy is neither merely a degradation pathway nor a binary process but a carefully tuned homeostatic program that determines whether the myocardium adapts or deteriorates under stress.

#### Mitophagy as a central component of cardiac mitochondrial quality control

Parallel to macroautophagy, mitophagy plays a particularly critical role in safeguarding mitochondrial quality control (MQC), especially given the extreme energetic demands of the heart. When mitophagy is dysregulated, damaged mitochondria accumulate, resulting in mitochondrial DNA damage, ROS amplification, energetic collapse, and cardiomyocyte death. In cardiac stress states such as myocardial infarction, mitophagy is frequently upregulated as a compensatory survival response to remove dysfunctional mitochondria and protect myocardial tissue.^30^ Impaired mitophagy contributes to cardiac dysfunction and fibrosis in obesity, primary cardiomyopathies, and HIV-associated cardiomyopathy (HIVAC).^31,32^ In HIVAC, disruptions in autophagy machinery lead to accumulation of abnormal mitochondria independent of viral entry pathways, whereas Highly Active Antiretroviral Therapy (HAART) agents such as azidothymidine and abacavir further exacerbate autophagy defects. Conversely, enhancing autophagy alleviates mitochondrial dysfunction and restores cardiomyocyte performance.

#### PINK1-Parkin-dependent mitophagy in cardiac stress and cardioprotection

Among mitophagy pathways, the PINK1-Parkin axis is one of the best characterized (Fig. 1a). Altered expression of PINK1 and Parkin, including reduced Parkin levels, correlates strongly with mitochondrial dysfunction and contractile impairment in heart failure.^33,34^ During ischemia‒reperfusion injury, ROS generation and mitochondrial fragmentation intensify; activation of PINK1-Parkin mitophagy removes damaged mitochondria, reduces oxidative stress, and limits apoptosis. Several therapeutic strategies exploit this pathway. Mitochondrion-targeted antioxidants such as MitoQ enhance PINK1-Parkin activity and reduce cardiomyocyte death.^35^ Gene therapy restoring PINK1 or Parkin expression yields cardioprotective effects.^36^ Interestingly, commonly used cardioprotective agents, including statins and ACE inhibitors, appear to indirectly enhance mitophagy signaling, highlighting their broader mechanistic relevance.^37^ Rapamycin and other mTOR inhibitors similarly promote combined autophagy-mitophagy responses, improving mitochondrial integrity and cardiac outcomes in multiple disease models.^38^

#### Receptor-mediated and lipid-driven mitophagy pathways in the heart

Beyond PINK1-Parkin, FUNDC1 has emerged as a key mitophagy regulator, particularly under hypoxia. Its activation during myocardial ischemia limits injury, whereas its deletion exacerbates ischemic damage.^39,40^ FUNDC1 also links mitophagy to mitochondrial fission through DRP1 and is modulated by upstream regulators such as ULK1 and PGAM5, offering multiple therapeutic entry points.^39,40^ Additional mitophagy receptors, including BNIP3 and NIX, contribute to stress-responsive mitochondrial clearance, particularly in hypoxic conditions.^41^ A complementary mechanism involves cardiolipin-mediated mitophagy, in which cardiolipin externalizes to the outer mitochondrial membrane and directly interacts with LC3 to trigger mitochondrial turnover.^42^ Stabilizing cardiolipin or enhancing its translocation improves mitochondrial clearance and reduces oxidative stress in heart failure models,^43^ while enzymes involved in cardiolipin metabolism, such as tafazzin, represent additional therapeutic opportunities.^44^

Together, autophagy and mitophagy form an integrated multilayer quality-control network essential for sustaining cardiac proteostasis, mitochondrial integrity, and mechanical performance. Their coordinated activation determines whether the heart maintains resilience under physiological demand and pathological stress or undergoes progressive failure. Understanding how to precisely modulate these pathways remains fundamental to advancing targeted therapies in cardiovascular disease.

### The role of the ubiquitin proteasome system (UPS) in the heart

#### The UPS as a central and highly selective proteostasis system in cardiomyocytes

Alongside autophagy and mitophagy, the ubiquitin‒proteasome system (UPS) represents a central pillar of cardiac proteostasis, responsible for the highly selective degradation of damaged, misfolded, or regulatory proteins and thereby safeguarding cellular homeostasis and protein turnover (Fig. 1a). Whereas autophagy can degrade large macromolecular complexes and even entire organelles, the UPS is characterized by exquisite substrate specificity, targeting only ubiquitinated proteins while preserving intact cellular structures. This selectivity is indispensable for normal cellular physiology, governing key processes such as cell cycle control, transcriptional and signal transduction regulation, receptor downregulation, endocytosis, immune responses, development, and apoptosis.

UPS-mediated degradation proceeds through a tightly ordered enzymatic cascade. Ubiquitin, a conserved 76-amino acid protein, is first activated in an ATP-dependent manner by E1 ubiquitin-activating enzymes, then transferred to E2 conjugating enzymes, and finally attached to target proteins by E3 ubiquitin ligases, which provide the critical element of substrate specificity (Fig. 2c). Ubiquitin is typically conjugated to lysine residues on substrate proteins, most classically via K48 linkages, forming polyubiquitin chains that are recognized by the 26S proteasome. Proteasomal processing results in complete proteolysis of the substrate, while deubiquitinating enzymes (DUBs) recycle ubiquitin molecules for subsequent rounds of degradation, ensuring the efficiency and sustainability of the pathway.

Although K48-linked ubiquitination is the canonical signal for proteasomal degradation, the UPS exhibits remarkable versatility through alternative ubiquitin linkages and chain architectures (Fig. 2c). K11-linked branched chains, for example, facilitate rapid turnover of key cell cycle regulators, whereas linkages such as K27, K29, and K33 can attenuate degradation, illustrating how chain topology fine-tunes proteolysis. Beyond proteasomal targeting, non-canonical ubiquitin modifications, including K63-linked polyubiquitination, regulate signaling, endocytosis, kinase activity, and DNA damage responses, while mono- and multi-ubiquitination influence chromatin dynamics, DNA repair, and subcellular localization (Fig. 2c). This combinatorial coding capacity underscores the adaptability of the UPS and its capacity to orchestrate diverse cellular outcomes beyond protein destruction.

In the heart, an organ composed largely of post-mitotic cardiomyocytes with limited regenerative capacity, UPS integrity is particularly critical. Failure of UPS surveillance promotes the accumulation of misfolded proteins, proteotoxic aggregates, fibrosis, and ultimately contractile dysfunction and heart failure. Thus, dissecting the contributions of E1, E2, and E3 components in cardiomyocytes is fundamental to understanding how UPS perturbations drive cardiovascular diseases.

#### E1 and E2 enzymes: upstream regulators of cardiac ubiquitination capacity

##### E1 activators: gatekeepers of the UPS pathway

The UPS pathway begins with the activation of ubiquitin by E1 activators, also known as ubiquitin-like modifier activating enzymes (UBA1-UBA7). These enzymes catalyze the ATP-dependent formation of a thioester bond between ubiquitin and the E1 active site, marking the first step in the ubiquitination cascade. Despite their critical role, there are relatively few E1-activators compared to the vast array of E3 ligases. Deficiencies in E1 activators can have fatal consequences for cardiac proteostasis, as they disrupt the entire ubiquitination process.^45^ Interestingly, while UBE1 expression remains unchanged in human heart failure, experimental studies have shown that overexpression of UBE1 or UBE6 in neonatal rat ventricular cardiomyocytes (NRVCMs) increases the ubiquitination of Nav1.5, a major transmembrane protein essential for cardiomyocyte depolarization. This suggests that modulating E1 activators could influence ion channel function and electrical activity in the heart, potentially offering a therapeutic avenue for arrhythmias.

##### E2-conjugating enzymes: intermediaries in ubiquitination

E2-conjugating enzymes act as intermediaries in the UPS pathway, transferring ubiquitin from E1-activators to substrate proteins, often in collaboration with E3 ligases. Alterations in E2 enzymes have been linked to various heart diseases. For instance, qPCR analysis of cardiomyocytes from patients with atrial fibrillation revealed an upregulation of E2D2, suggesting a role for this enzyme in the pathogenesis of arrhythmias.^46^ Similarly, the mRNA levels of E2G2 and UBC2 are elevated in human heart failure, implicating these enzymes in the progression of cardiac dysfunction.^47^ These findings highlight the potential of E2 enzymes as biomarkers and therapeutic targets in cardiovascular diseases.

#### E3 ubiquitin ligases as determinants of cardiac remodeling and stress adaptation

Among UPS components, E3 ubiquitin ligases play a particularly decisive role owing to their ability to recognize specific substrates. Their diversity reflects the complexity of the proteome: mammals encode over 600 E3 ligases, far exceeding the number of E1 and E2 enzymes. E3 ligases are broadly categorized according to their catalytic domains. HECT and RBR ligases form covalent thioester intermediates with ubiquitin prior to substrate transfer, whereas RING-type ligases, the most abundant class, mediate the direct transfer of ubiquitin from E2 to the substrate.^48^ Together with the diversity of ubiquitin linkage types, this architectural variability gives the UPS its extraordinary precision, enabling tight regulation of proteostasis and preventing the accumulation of toxic proteins under physiological and pathological stress.

E3 ligases are the most diverse and functionally specialized components of the UPS and are responsible for substrate recognition and the final transfer of ubiquitin to target proteins. They regulate cardiomyocyte growth, stress adaptation, signal transduction, ion channel turnover, autophagy-proteasome crosstalk, congenital heart disease mechanisms, and cardiomyopathies. Most cardiac E3 ligases belong to the RING-type family, although both single- and multi-subunit variants exist, each executing distinct regulatory roles. For example, the cardioprotective E3 ligase HectD3 suppresses Calcineurin-NFAT signaling and inflammatory stress by targeting SUMO2 and Stat1, thereby mitigating hypertrophic remodeling.^49^ Conversely, individual TRIM family ligases exert opposing influences: TRIM32 promotes hypertrophy via dysbindin regulation, whereas TRIM24 restrains hypertrophic signaling, illustrating the nuanced, context-dependent contribution of E3 ligases to cardiac physiology and pathology.^50^

### The role of endoplasmic reticulum-associated degradation (ERAD) in the heart

The ERAD pathway is a critical quality control mechanism that ensures the proper folding and degradation of misfolded or aberrant proteins within the ER^51^ (Fig. 1a). This pathway plays a vital role in maintaining cellular homeostasis, particularly in the heart, where protein misfolding and aggregation can have severe consequences for cardiac function. Dysregulation of ERAD can lead to the accumulation of misfolded proteins, chronic ER stress, and ultimately, cardiac dysfunction.

The role of membrane proteolysis, particularly through pathways such as ERAD and intramembrane proteolysis, is crucial for cardiovascular health. ERAD is involved in modulating the degradation of proteins that accumulate during myocardial stress responses, such as those seen in ischemia or heart failure.^52^ For example, during ischemic injury, the heart experiences an overload of misfolded proteins due to oxidative stress and energy depletion. ERAD helps mitigate this burden by degrading these proteins, thereby preventing their aggregation and the subsequent activation of detrimental pathways, such as chronic ER stress, autophagy dysfunction, and apoptotic signaling. Dysregulation of ERAD can lead to an accumulation of misfolded proteins in cardiomyocytes, contributing to the pathogenesis of heart disease. Chronic ER stress, resulting from impaired ERAD, triggers the UPR, which, if unresolved, can lead to cardiomyocyte apoptosis and heart failure. Additionally, ERAD dysfunction can disrupt lipid metabolism and cholesterol synthesis by impairing the degradation of SREBPs, further exacerbating cardiovascular disease.^53^

### Therapeutic targeting of cardiac proteostasis

The recognition that proteostasis failure contributes directly to cardiovascular disease has accelerated the development of therapeutic strategies aimed at restoring protein quality control rather than targeting isolated downstream manifestations.

#### Targeting molecular chaperones and heat shock proteins

Molecular chaperones, particularly HSPs, represent attractive therapeutic targets due to their central role in stabilizing misfolded proteins and suppressing aggregation under cardiac stress. HSP90 inhibitors such as ganetespib (STA-9090) and AUY922 have been extensively evaluated in oncology,^54,55^ where they disrupt the stabilization of oncogenic client proteins. Although their clinical development has focused primarily on cancer, emerging studies are beginning to explore whether controlled modulation of HSP90 activity may also influence maladaptive proteostasis in cardiovascular disease.

In contrast, therapeutic strategies targeting other HSPs, including HSP70, HSP27, and αB-crystallin, remain at an earlier stage but have demonstrated encouraging results in preclinical cardiovascular models. Overexpression of HSP70 enhances the resistance of cardiomyocytes to ischemia-like stress and improves functional recovery following reperfusion. Similarly, HSP27 overexpression reduces infarct size and preserves cardiac function in animal models of myocardial infarction.^56–58^ Collectively, these findings suggest that selective enhancement of cardioprotective chaperone functions, rather than broad inhibition, may offer a more viable and safer strategy for HSP-based interventions in heart disease.

#### Therapeutic modulation of autophagy in cardiovascular disease

Autophagy has emerged as one of the most actively investigated proteostasis pathways for therapeutic intervention in cardiovascular disease. Among currently available agents, hydroxychloroquine (HCQ) and chloroquine (CQ) are the most widely studied autophagy inhibitors in clinical use. Originally developed as antimalarial drugs, these compounds inhibit autophagy by blocking autophagosome-lysosome fusion, thereby preventing the degradation of autophagic cargo. HCQ and CQ are now being evaluated in combination regimens for multiple cancers, including pancreatic, breast, and lung malignancies.^59^

However, their application in cardiovascular disease has raised important safety concerns. In patients with pre-existing heart failure, autophagy inhibition may exacerbate proteotoxic stress rather than confer benefit, prompting cautious evaluation in cardiac clinical trials.^60^ These observations highlight the necessity of disease-stage-specific and quantitatively controlled autophagy modulation.

To overcome the limitations of first-generation inhibitors, next-generation autophagy inhibitors such as Lys05 and DQ661 have been developed. These compounds demonstrate greater potency and improved specificity in preclinical models. Lys05 exhibits enhanced autophagy inhibition and antitumor activity, while DQ661 targets both autophagy and lysosomal function, offering a dual mechanism of action.^61^ Although currently pursued primarily in oncology, these agents may ultimately enable more refined autophagy modulation in cardiovascular settings.

In parallel, several established drugs are being repurposed for their autophagy-modulating properties. Metformin, an AMP-activated protein kinase (AMPK) activator widely used in diabetes, is currently in phase I clinical trials for heart failure with preserved ejection fraction (HFpEF) (Tables 1 and 2). Metformin enhances autophagy, reduces cardiac hypertrophy, and shows promise for improving functional outcomes in heart failure. Rapamycin, an mTOR inhibitor, similarly promotes autophagy, attenuates pathological remodeling and is now in phase II clinical trials for HFpEF (Tables 1 and 2).

Sodium-glucose cotransporter-2 (SGLT2) inhibitors, including empagliflozin, canagliflozin, and dapagliflozin, represent another important class of repurposed agents with robust cardiovascular benefits. Originally developed for glycemic control, these drugs are increasingly recognized for their capacity to induce autophagy, which may contribute to their cardioprotective effects. Notably, the EMPEROR-Preserved trial demonstrated that empagliflozin significantly reduced cardiovascular mortality and hospitalization in HFpEF patients without increasing adverse events.^62^

#### UPS-targeted therapies and induced protein degradation strategies

Recent advances in targeted protein degradation have transformed the ubiquitin‒proteasome system into a highly versatile therapeutic platform. Central to this approach are degrons, short amino acid motifs, typically 6–10 residues in length, that mark proteins for recognition by E3 ubiquitin ligases. Large-scale CRISPR-based screens have identified thousands of degron motifs, particularly at protein C-termini, regulated by Cullin-RING E3 ligase complexes such as CRL2 and CRL4.^63^ Proteomic analyses further suggest selective depletion of C-terminal degrons in eukaryotic proteins, supporting the existence of “C-end rules” that shape proteome composition.^63^ These insights have important implications for therapeutic manipulation of cardiac proteostasis.

Building upon degron biology, molecular glues have been developed to induce selective protein degradation. Compounds such as thalidomide and its analogs lenalidomide and pomalidomide promote interactions between E3 ligases (e.g., cereblon) and specific target proteins, triggering their ubiquitination and proteasomal degradation. Molecular glues are already approved for multiple myeloma and myelodysplastic syndromes and are under investigation for additional malignancies.^64^ Their success has opened new opportunities for targeting disease-associated proteins previously considered undruggable, with emerging relevance for cardiovascular disorders.

Another major innovation is the development of proteolysis-targeting chimeras (PROTACs). These bifunctional molecules link a ligand for the target protein to a ligand recruiting an E3 ubiquitin ligase, such as VHL, MDM2, CRBN, or IAPs.^65,66^ By forcing proximity between the target and ligase, PROTACs induce polyubiquitination and proteasomal degradation. Although initially developed for oncology, PROTAC-based strategies are now being explored in cardiovascular disease, where selective degradation of pathogenic proteins could mitigate hypertrophy, fibrosis and heart failure progression.

Deubiquitinating enzymes (DUBs) represent an additional regulatory layer with therapeutic potential. Ubiquitin-specific peptidase 14 (USP14) removes ubiquitin chains from proteasome-bound substrates, thereby modulating protein stability. Inhibitors of USP14 are being developed to enhance the degradation of oncogenic proteins.^67^ While current efforts focus on cancer, similar strategies may be adaptable to cardiovascular disease, where enhanced clearance of misfolded or damaged proteins could improve outcomes in heart failure and ischemic injury.

#### Therapeutic targeting of ER-associated degradation and ER stress

ER-associated degradation and unfolded protein response signaling represent critical targets for alleviating proteotoxic stress in cardiomyocytes. One promising strategy involves modulation of activating transcription factor 6 (ATF6), a key mediator of adaptive UPR signaling. Small-molecule activators of ATF6 are being developed to enhance ER folding capacity, stimulate ERAD, and reduce ER stress, with potential benefits in heart failure and ischemic cardiomyopathy.^68^

Enhancement of specific ERAD components offers another therapeutic avenue. E3 ubiquitin ligases such as Hrd1 play a central role in targeting misfolded ER proteins for degradation. Pharmacological upregulation of Hrd1 expression or activity may accelerate the clearance of misfolded proteins, thereby reducing ER stress and preventing cardiomyocyte apoptosis, an approach particularly relevant in diabetic cardiomyopathy.^69^ Proteasome activators provide an additional strategy to support ERAD function. By increasing the overall degradation capacity, these agents facilitate more efficient clearance of ubiquitinated proteins and reduce the proteotoxic burden. Preclinical studies have demonstrated beneficial effects of proteasome activation in models of heart failure and ischemia‒reperfusion injury,^69^ further supporting ER proteostasis as a therapeutically actionable axis.

Together, these therapeutic advances underscore the emergence of cardiac proteostasis as a druggable, systems-level target. Rather than manipulating individual pathways in isolation, effective intervention will likely require coordinated, context-dependent modulation of chaperones, autophagy, UPS activity, and ER stress responses. As precision medicine approaches advance, targeting proteostasis networks holds substantial promise for preserving myocardial function, slowing disease progression and reshaping the future of cardiovascular therapeutics.

## Metabolic diseases and protein homeostasis

**Fig. 3:**
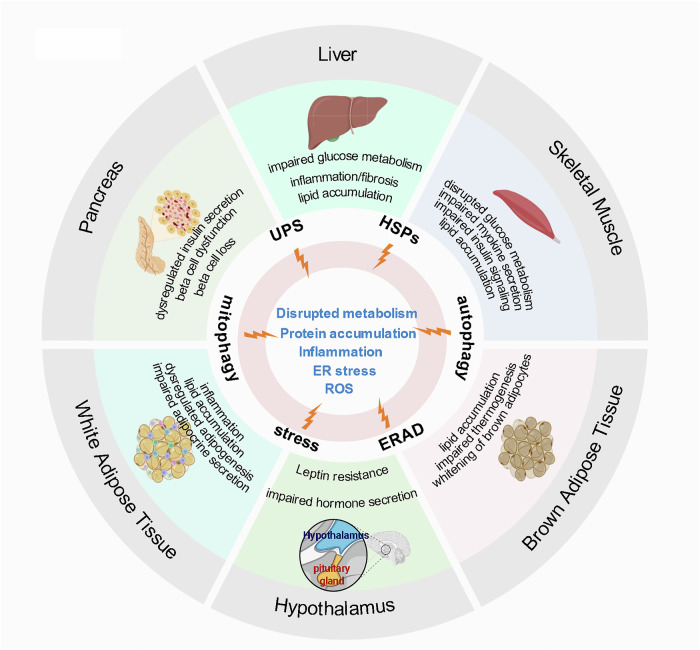
Systemic breakdown of proteostasis networks in metabolic diseases. This schematic representation illustrates the breakdown of the proteostasis system in metabolic diseases, as discussed in this review. It highlights how metabolic stress disrupts proteostasis across various tissues, including the liver, skeletal muscle, pancreas, adipose tissue, and hypothalamus. Metabolic stressors, such as chronic inflammation, endoplasmic reticulum (ER) stress, and excessive reactive oxygen species (ROS) production, compromise cellular homeostasis, impair protein folding and degradation, and disrupt metabolic pathways. This results in the accumulation of misfolded proteins and dysfunctional organelles, further exacerbating cellular stress. The dysregulation of key proteostasis mechanisms, including endoplasmic reticulum-associated degradation (ERAD), heat shock proteins (HSPs), and the ubiquitin-proteasomal system (UPS), leads to tissue-specific alterations that drive the progression of metabolic diseases such as obesity, type 2 diabetes mellitus (T2DM), and metabolic dysfunction-associated steatotic liver disease (MASLD). This figure underscores the central role of proteostasis failure in the pathophysiology of metabolic disorders and its potential as a therapeutic target. This figure was created at Biorender.com

Metabolic syndrome (MetS) and its associated disorders, such as type 2 diabetes mellitus (T2DM), obesity, non-alcoholic fatty liver disease (NAFLD), and CVDs, represent a significant global health burden. These interconnected conditions arise from systemic metabolic dysfunction, characterized by insulin resistance, dyslipidemia, chronic low-grade inflammation, and ectopic fat deposition. At the cellular level, proteostasis is emerging as a critical factor in the onset and progression of MetS. The failure of proteostasis mechanisms under metabolic stress contributes to the accumulation of misfolded proteins, defective organelles, and disrupted signaling pathways, thereby amplifying metabolic dysfunction and tissue damage (Fig. 3).

Metabolic stressors such as nutrient excess, oxidative stress, and inflammation challenge the proteostasis network by overwhelming the protein-folding capacity of cells and impairing protein degradation systems. Key proteostasis pathways, including the UPS, ALP, HSPs, and UPR, are critical for regulating cellular homeostasis under these stress conditions (Fig. 1a). For instance, the UPS mediates the targeted degradation of regulatory proteins involved in insulin signaling and lipid metabolism, while autophagy clears damaged organelles such as mitochondria, which are central to energy metabolism. Dysregulation of these pathways contributes to the pathogenesis of MetS by impairing glucose and lipid homeostasis, exacerbating insulin resistance, and promoting inflammatory responses (Fig. 3).

### The role of HSPs in metabolic diseases

#### HSPs as central modulators of metabolic proteostasis

Metabolic diseases, including obesity, T2DM, and NAFLD, now classified as MASLD, are characterized by chronic cellular stress that disrupts protein homeostasis.^70^ This sustained imbalance manifests as ER stress, oxidative damage, mitochondrial dysfunction, and chronic low-grade inflammation, all of which accelerate disease progression. Central to the cellular defense machinery against such stress are heat shock proteins. By facilitating proper protein folding, stabilizing protein conformations, and limiting proteotoxic stress, HSPs play essential protective roles in metabolically active tissues. However, when dysregulated, these same pathways can exacerbate inflammation and metabolic dysfunction. Accordingly, altered HSP expression has been implicated in the pathogenesis of multiple metabolic disorders, positioning HSPs as both biomarkers of disease severity and promising, albeit complex, therapeutic targets (Fig. 3).

#### HSP70/HSP72 in insulin resistance and metabolic homeostasis

Among the HSP family, HSP70, particularly its inducible isoform HSP72 (also referred to as intracellular HSP72 or iHSP72), has been most extensively studied in metabolic disease contexts. Reduced iHSP72 levels in skeletal muscle and other insulin-responsive tissues are strongly associated with insulin resistance, a defining feature of T2DM. Experimental evidence indicates that iHSP72 mitigates metabolic dysfunction by suppressing inflammatory signaling, enhancing mitochondrial function, and stabilizing proteostasis. Mechanistically, iHSP72 inhibits the activation of stress kinases such as c-Jun N-terminal kinase (JNK), which plays a central role in promoting insulin resistance and metabolic inflammation. Consistent with these observations, animal models with iHSP72 overexpression exhibit improved insulin sensitivity and reduced systemic inflammation, reinforcing its therapeutic relevance.^71^

In the context of MASLD, iHSP72 exerts hepatoprotective effects by attenuating oxidative stress, reducing lipid accumulation, and limiting inflammatory responses. Increased iHSP72 expression in hepatocytes prevents steatosis and liver injury, underscoring its role in preserving hepatic metabolic homeostasis.^71,72^ In contrast, extracellular HSP72 (eHSP72), which is released into circulation under conditions of cellular stress, is often associated with adverse metabolic outcomes. Elevated circulating eHSP72 levels correlate with chronic inflammation, insulin resistance, and pancreatic β-cell dysfunction in T2DM patients. Moreover, high eHSP72 levels have been linked to diabetic complications such as ketoacidosis, highlighting the importance of maintaining a finely tuned balance between intracellular and extracellular HSP72 for therapeutic intervention.^73^

#### Contributions of other HSPs to metabolic disease pathogenesis

Beyond HSP72, several other HSP family members contribute to metabolic disease development in tissue-specific and context-dependent manners.

##### HSP27

This small heat shock protein plays a dual role in metabolic diseases. In hepatic cells, phosphorylated HSP27 promotes autophagy and lipid clearance, suggesting its therapeutic potential in MASLD and non-alcoholic steatohepatitis (NASH), now referred to as metabolic dysfunction-associated steatohepatitis (MASH).^74^ Conversely, extracellular HSP27 has been associated with diabetic complications, including nephropathy and platelet aggregation, emphasizing its complex role in disease progression.^75^

##### HSP60

As a mitochondrial chaperone, HSP60 facilitates proper folding of mitochondrial proteins and helps maintain MQC. Dysregulation of HSP60 is a hallmark of metabolic disorders, with elevated circulating HSP60 levels linked to insulin resistance and cardiovascular complications in T2DM patients.^76^ Interestingly, reduced hepatic HSP60 levels correlate with increased steatosis and inflammation in MASLD, suggesting its tissue-specific roles in metabolic dysfunction.^77^ Overexpression of HSP60 in animal models alleviates fat accumulation and improves mitochondrial function, underscoring its therapeutic potential.^77^

##### HSP90

HSP90, particularly the β isoform, has been implicated in lipid metabolism and cholesterol homeostasis. Increased HSP90 expression has been observed in MASLD, where it contributes to lipid imbalance and metabolic dysfunction.^78^ Pharmacological inhibition of HSP90 improves insulin sensitivity and reduces inflammation in experimental models, suggesting that selective targeting of HSP90 may be beneficial in certain metabolic contexts.^78^ However, because HSP90 also stabilizes numerous client proteins essential for cellular survival, its dual roles underscore the challenges associated with therapeutic modulation and the need for precise, context-dependent strategies.

### The role of autophagy and mitophagy in metabolic diseases

Autophagy plays a pivotal role in metabolic health by regulating nutrient sensing, energy homeostasis, and stress responses. Dysregulated autophagy is increasingly recognized as a key contributor to the pathogenesis of metabolic diseases, including obesity, T2DM, and MASLD (Fig. 3).

#### Adipose tissue: autophagy at the intersection of lipid metabolism and inflammation

Adipose tissue represents a primary site where autophagy contributes to metabolic health. In obesity, autophagy markers such as LC3-II and autophagosome formation are elevated, accompanied by reduced mTORC1 activity, particularly in visceral depots.^79^ This upregulation correlates with obesity severity, adipocyte hypertrophy, and adverse glucometabolic status, reflecting a tightly regulated interplay between autophagy, tissue remodeling, and metabolic stress.^80^ Early obesity appears to engage autophagy as a compensatory defense against inflammation and lipid burden^81^; however, chronic exposure skews this response toward dysfunction. Maternal obesity similarly disrupts autophagy/mitophagy pathways, contributing to metabolic disturbances that adversely impact fetal development and postnatal metabolic health.^82^

Genetic models reinforce the essential role of autophagy in adipose biology. Deletion of Becn2, Atg7, Tfeb, or Bif predisposes individuals to obesity and insulin resistance, highlighting autophagy’s role in systemic metabolic regulation.^83–85^ Mechanistically, autophagy stabilizes PPARγ2, a master transcriptional regulator of adipogenesis; inhibition accelerates its proteasomal degradation and suppresses adipocyte differentiation.^86^ Moreover, Atg5- and Atg7-dependent autophagy supports lipid droplet biogenesis and hormone-driven lipolysis, as their inhibition reduces lipid accumulation and adipogenic marker expression.^87^ Together, these observations underscore autophagy as a determinant of adipocyte function, lipid storage dynamics, and inflammatory tone.

#### Hypothalamic autophagy: orchestrating whole-body energy balance

Beyond peripheral tissues, autophagy in the hypothalamus governs systemic energy homeostasis by integrating nutrient signals and adiposity cues. Impaired hypothalamic autophagy activates IKKβ signaling, promoting inflammation and disrupting metabolic regulation.^88^ In POMC neurons, autophagy is required for intact leptin signaling and appetite suppression; its disruption aggravates leptin resistance, hyperphagia, and weight gain.^89^ Importantly, restoration of hypothalamic autophagy via nutritional interventions such as docosahexaenoic acid-acylated astaxanthin monoester (AST-DHA) supplementation or genetic activation of Atg5 ameliorates obesity and improves glucose metabolism in experimental models,^90^ demonstrating that hypothalamic autophagy is not merely correlative but mechanistically regulatory.

#### Autophagy, lipophagy, and hepatic homeostasis in MASLD

In the liver, autophagy is indispensable for maintaining cellular quality control by eliminating lipid droplets, damaged organelles, and toxic aggregates. Its impairment is a hallmark of MASLD and a key driver of disease progression toward MASH, fibrosis, and hepatocellular carcinoma.^91^ Although autophagosomes are often increased in MASLD, reductions in BECN1 and accumulation of p62/SQSTM1 indicate defective lysosomal degradation and impaired autophagic flux.^1^, ^92^ This dysfunction affects lipophagy, insulin signaling, inflammatory responses, and cellular stress pathways.

Lipophagy, a selective form of autophagy targeting lipid droplets, plays a central role in regulating hepatic triglyceride turnover. In early MASLD, lipophagy is induced to mitigate lipid overload; however, persistent metabolic stress progressively impairs this process, promoting triglyceride accumulation, inflammation, and hepatocellular injury.^93–96^ Dysfunctional lysosomes and genetic variants such as IRGM further exacerbate disease progression.^97^ mTORC1 functions as a major suppressor of lipophagy, and its inhibition restores autophagic flux and improves hepatic lipid handling.^98^ TFEB-mediated lysosomal biogenesis is similarly essential for efficient lipophagy, while natural compounds such as naringin enhance autophagic flux and reduce steatosis in experimental MASLD.^99^ These findings place autophagy-lipophagy failure at the mechanistic core of MASLD pathogenesis.

#### Mitophagy in obesity, insulin resistance, and MASLD

Complementing macroautophagy, mitophagy preserves mitochondrial quality control and thereby protects against cellular stress, inflammation, and metabolic deterioration. In obesity and insulin resistance, mitophagy markers, including LC3BII, Parkin, FUNDC1, and BNIP3, are generally reduced in both human and animal studies, reflecting compromised mitochondrial turnover. Although initially activated to buffer metabolic stress, the PINK1-Parkin pathway becomes suppressed under chronic overload, such as prolonged palmitate exposure, resulting in dysfunctional mitochondria, heightened oxidative stress, and activation of the NLRP3 inflammasome.^100–102^

A similar biphasic pattern occurs in MASLD: mitophagy is enhanced during early disease to counteract lipid excess and maintain mitochondrial homeostasis, but prolonged metabolic stress suppresses this adaptive response.^103,104^ Consequences include excessive ROS production, progressive lipid accumulation, mitochondrial swelling, cristae disruption, impaired β-oxidation, fibrosis, inflammasome activation, and transition from steatosis to MASH.^103,105,106^ Thus, mitophagy impairment functions as a critical molecular turning point converting compensatory metabolic adaptation into persistent injury and organ failure.

### The role of the UPS in metabolic diseases

Dysfunction of the UPS has emerged as a key factor in the development of metabolic disorders, including obesity, T2DM, and MASLD. These conditions are characterized by cellular stress, chronic inflammation, and disrupted lipid and glucose metabolism, all of which are influenced by UPS activity (Fig. 3).

#### UPS dysfunction in metabolic diseases

Metabolic diseases are often accompanied by heightened oxidative stress, driven by ROS produced by malfunctioning mitochondria and persistent inflammation. When oxidative stress overwhelms cellular defenses, it can impair the 26S proteasome, leading to the accumulation of ubiquitinated proteins and disrupting protein homeostasis.^107^ Similarly, ER stress, a hallmark of metabolic diseases, places additional demand on the UPS through the activation of the UPR. While the UPR initially enhances proteasome activity to manage misfolded proteins, chronic ER stress can overwhelm the UPS, compromise proteasome function and exacerbate cellular dysfunction. Proteasome activity is notably reduced in several tissues in obesity and T2DM. This has been demonstrated in animal studies and confirmed in humans, where impaired proteasome function in adipocytes is strongly linked to insulin resistance.^108^ Interestingly, plasma levels of ubiquitin and 26S proteasomes are lower in obese individuals, showing an inverse correlation with body mass index (BMI).^109^ Preclinical studies further indicate that alterations in the UPS contribute to the progression and perpetuation of obesity and its complications.^110^ However, the role of the UPS is tissue specific, e.g., in muscle cells from insulin-resistant individuals, proteasome activity is increased, contributing to muscle protein degradation and atrophy.^111^ This highlights the complex and context-dependent role of the UPS in metabolic diseases.

#### E3 ligases in metabolic diseases

Insulin receptors and insulin receptor substrates (IRS-1 and IRS-2) are crucial for insulin signaling, and E3 ligases such as MG53, c-Cbl, and Nedd4 can directly degrade these substrates, disrupting signaling and promoting insulin resistance.^112^ Animal studies have shown that suppressing the activity of MG53 and SOCS1 improves insulin sensitivity and protects against metabolic dysfunction.^113,114^ In MASLD and MASH, E3 ligases play dual roles, with both protective and pathological implications. Some proteins, such as TRIM31 and Gp78, mitigate liver damage by promoting lipid turnover and reducing inflammation.^115^ Others, such as Siah1 and TRIM8, exacerbate the condition by driving lipid accumulation and inflammation.^116,117^ Additionally, Smurf1 stabilizes sterol regulatory element-binding protein-1c (SREBP-1c), a key transcription factor in lipid metabolism, by preventing its ubiquitination and degradation, while BTRC promotes the proteasomal degradation of adipose triglyceride lipase.^118,119^

Chronic inflammation, a hallmark of metabolic disorders, is also influenced by E3 ligases. For instance, deleting Pellino3 exacerbates insulin resistance by increasing pro-inflammatory macrophages, while inhibiting ITCH protects against steatohepatitis by promoting anti-inflammatory macrophages.^120,121^ In atherosclerosis, a metabolic disease driven by chronic inflammation and faulty lipid metabolism, E3 ligases such as FBXW2 and FBXO3 have been identified as key modulators of vascular inflammation.^122,123^ Other E3 ligases, including Peli1, play vital roles in atherosclerotic plaque stability and foam cell formation, suggesting that targeting these ligases could impact disease progression.^124^

#### DUBs in metabolic disorders

DUBs, which reverse ubiquitination and regulate protein stability, localization, and activity, play critical roles in metabolic pathways and demonstrate both detrimental and protective effects in metabolic diseases. For example, USP19 promotes adipogenesis and exacerbates high-fat-diet-induced obesity and glucose intolerance in mice.^125^ USP21, upregulated in the skeletal muscles of obese individuals, impairs mitochondrial activation and carbohydrate metabolism. Ablation of USP21 in mouse skeletal muscle ameliorates obesity and insulin resistance.^126^ USP20 stabilizes HMG-CoA reductase (HMGCR), the rate-limiting enzyme in cholesterol biosynthesis, and its inhibition reduces lipid levels and improves insulin sensitivity in preclinical models.^127^ USP14 and USP2 deubiquitinate fatty acid synthase (FASN), a key enzyme in lipogenesis, aggravating hepatic steatosis and insulin resistance.^128,129^ Conversely, USP4, USP10, and USP18 mitigate metabolic dysfunction by reducing hepatic steatosis, hyperglycemia, and inflammation, suggesting therapeutic potential against MASLD and T2DM.^130–132^ USP7 improves insulin sensitivity and suppresses gluconeogenesis, offering another example of a DUB with beneficial effects on glucose homeostasis.^133^

### The role of ERAD and UPR in metabolic diseases

ER stress is a hallmark of metabolic diseases such as T2DM, insulin resistance, and MASLD. Under physiological conditions, ERAD and the UPR cooperate to maintain proteostasis by ensuring proper folding, processing, and clearance of secretory and membrane proteins. When ERAD capacity is overwhelmed or dysfunctional, unresolved ER stress ensues, driving metabolic dysregulation and disease progression (Fig. 3).

#### ERAD as a central metabolic checkpoint: the Sel1L-HRD1 axis

Among ERAD components, the Sel1L-HRD1 complex is highly conserved across species and has emerged as a central regulator of systemic metabolism, influencing diverse processes ranging from appetite control to lipid handling and insulin secretion.^134^ The Sel1L-HRD1 ERAD complex functions as a metabolic checkpoint that integrates nutrient availability, hormonal signaling, and cellular stress responses across multiple tissues. By governing the degradation of key metabolic regulators, this complex ensures adaptive metabolic flexibility under fluctuating physiological demands. Disruption of Sel1L-HRD1 activity leads to tissue-specific ER stress and systemic metabolic imbalance, underscoring its pivotal role in maintaining whole-body homeostasis.^134^

#### Neuroendocrine control of energy balance: ERAD in appetite and nutrient absorption

At the level of the central nervous system, ERAD plays a decisive role in the neuroendocrine regulation of energy balance. In the hypothalamus, the Sel1L-HRD1 complex is indispensable for proper leptin signaling and the folding of POMC, a precursor of neuropeptides that suppress appetite and regulate energy expenditure. Disruption of ERAD in this context impairs leptin responsiveness, resulting in hyperphagia, excessive weight gain, and heightened susceptibility to diet-induced obesity in mice.^135,136^ ERAD additionally contributes to metabolic regulation in the gastrointestinal tract. In the small intestine, ERAD controls the stability of enzymes involved in lipid absorption. Inhibition of ERAD leads to the accumulation of these enzymes, enhancing dietary fat uptake and promoting obesity.^137^ Together, these findings position ERAD as a critical regulator of energy intake by coordinating central appetite control with peripheral nutrient absorption.

#### ERAD in adipose tissue: thermogenesis, lipid handling, and systemic crosstalk

Beyond energy intake, ERAD exerts profound effects on energy expenditure and lipid distribution through its actions in adipose tissue. In brown adipose tissue, the Sel1L-HRD1 complex is essential for maintaining mitochondrial integrity and thermogenic capacity. BAT-specific disruption of ERAD results in abnormal mitochondrial morphology, impaired heat production, and increased cold sensitivity, highlighting the importance of ERAD in adaptive thermogenesis.^138,139^ Interestingly, adipocyte-specific Sel1L deficiency produces a distinct systemic phenotype. Although these mice are resistant to diet-induced obesity, they exhibit elevated postprandial triglyceride levels, reflecting dysregulated lipid handling rather than reduced caloric intake. Mechanistically, Sel1L stabilizes lipoprotein lipase (LPL) in adipocytes, thereby controlling its secretion and regulating lipid clearance from circulation.^134^ These observations underscore the complex and context-dependent effects of ERAD in adipose tissue, linking local proteostasis to systemic lipid homeostasis.

#### Hepatic ERAD as a circadian and lipogenic regulator

The liver represents a major metabolic hub where ERAD intersects with circadian and transcriptional control of metabolism. Hepatic Sel1L-HRD1 activity exhibits circadian rhythmicity mediated by the transcription factor CREBH and the nuclear receptor PPARα. Through regulated turnover of CREBH, ERAD orchestrates the rhythmic expression of genes involved in fatty acid oxidation, triglyceride lipolysis, and gluconeogenesis. Disruption of this regulatory axis perturbs energy balance and compromises metabolic homeostasis.^140^ ERAD also modulates hepatic endocrine signaling by controlling the transcription of FGF21, a hormone that enhances energy expenditure and insulin sensitivity. HRD1-mediated degradation of CREBH regulates FGF21 expression, linking ERAD directly to systemic metabolic adaptation.^141^ Notably, mice with hepatocyte-specific HRD1 deficiency display increased energy expenditure and resistance to high-fat diet-induced obesity and insulin resistance.^142^ In addition, ERAD directly restrains hepatic lipogenesis by targeting ATP-citrate lyase (Acly), a key enzyme that channels citrate toward fatty acid synthesis. HRD1-mediated degradation of Acly reduces hepatic lipid accumulation, thereby mitigating MASLD and reinforcing ERAD’s protective role against metabolic liver disease.^143^

#### ERAD in lipoprotein and cholesterol homeostasis

ERAD further shapes metabolic health by regulating cholesterol and lipoprotein metabolism. A central function of ERAD is the degradation of 3-hydroxy-3-methylglutaryl-CoA reductase (HMGCR), the rate-limiting enzyme in cholesterol biosynthesis. Dysregulation of this pathway leads to excessive cholesterol accumulation and contributes to hypercholesterolemia, a common feature of insulin resistance and metabolic syndrome.^144^ ERAD also governs the quality control of apolipoprotein B (ApoB), a critical structural component of very-low-density lipoproteins (VLDL). When ApoB fails to fold correctly, it is targeted for ERAD-mediated degradation, preventing aberrant VLDL assembly and secretion.^145^ Perturbations in this quality-control mechanism exacerbate hyperlipidemia and hepatic steatosis, further illustrating how ERAD links intracellular proteostasis to circulating lipid levels and cardiometabolic risk.

#### ERAD safeguards pancreatic β-cell identity and insulin secretion

Pancreatic β-cells are uniquely vulnerable to ER stress due to their high insulin biosynthetic demand. ERAD plays a protective role by clearing misfolded insulin and maintaining ER function, thereby supporting glucose-stimulated insulin secretion (GSIS). The Sel1L-HRD1 complex is essential for preserving β-cell identity and secretory competence.^146^ Mice with β-cell-specific disruption of Sel1L-HRD1 develop early-onset diabetes and glucose intolerance, reflecting impaired insulin secretion and β-cell dysfunction. Importantly, Sel1L expression is reduced in human islets from T2DM patients, suggesting that ERAD impairment contributes directly to β-cell failure and disease progression.^146^ These findings position ERAD as a critical safeguard of endocrine pancreatic function and a potential therapeutic target for diabetes.

### Therapeutic targeting of proteostasis in metabolic diseases

#### Targeting HSPs in metabolic diseases

Given their central roles in metabolic homeostasis, heat shock proteins (HSPs) have emerged as attractive therapeutic targets in metabolic disease. Multiple strategies have been explored to modulate HSP activity, including pharmacological agents, lifestyle interventions, and emerging gene therapy approaches.

##### Pharmacological modulators

Among pharmacological modulators, the small-molecule HSP72 activator BGP-15 has shown notable promise in both preclinical studies and early-phase clinical trials. By enhancing inducible HSP72 (iHSP72) expression, BGP-15 improves insulin sensitivity, increases glucose uptake, and reduces systemic inflammation.^147^ In addition, BGP-15 exerts protective effects against diabetic complications, including retinopathy and cardiomyopathy, supporting its potential utility in T2DM and related metabolic disorders. Natural compounds targeting HSP90, such as lipoic acid and isoquercitrin, have also demonstrated efficacy in reducing hepatic fat accumulation and inflammation, highlighting their potential for the treatment of MASLD and MASH.^148,149^

##### Lifestyle interventions

Lifestyle interventions represent a complementary, non-invasive means of modulating HSP expression. Physical activity robustly induces iHSP72 expression, improving insulin sensitivity, reducing inflammation, and enhancing mitochondrial function in individuals with MetS.^150^ Similarly, heat therapy, which stimulates HSP72 expression via controlled thermal stress, has been associated with reductions in fasting glucose, insulin resistance, and HbA1c levels in patients with T2DM.^151^ These findings underscore the therapeutic relevance of lifestyle-based strategies as adjuncts to pharmacological interventions.

##### Gene therapy and emerging approaches

Emerging gene therapy approaches further expand the therapeutic landscape. Viral-mediated delivery of HSP72 or HSP60 has demonstrated efficacy in preclinical models, improving metabolic parameters and preventing disease progression.^152,153^ Although still at an early stage of development, such approaches may ultimately benefit patients with severe or refractory metabolic disease.^71^

#### Therapeutic modulation of the autophagy-mitophagy axis in metabolic diseases

##### Pharmacological interventions

Several pharmacological agents already in clinical use exert metabolic benefits, at least in part, through activation of autophagy and mitophagy. SGLT2 inhibitors, including empagliflozin, reduce blood glucose and body mass while enhancing hepatic autophagy through the AMPK-TET2 and AMPK/mTOR signaling pathways, thereby attenuating hepatic steatosis.^154^ Similarly, glucagon-like peptide-1 (GLP-1) receptor agonists such as liraglutide and exenatide improve lipid metabolism and slow MASLD progression by activating TFEB-regulated autophagy-lysosomal pathways in conjunction with AMPK/mTOR signaling.^155^ Beyond their effects on bulk autophagy, these agents also improve mitochondrial quality control, contributing to improved cellular bioenergetics and metabolic resilience.

##### Natural compounds

Natural compounds further highlight the therapeutic relevance of autophagy-mitophagy modulation. Resveratrol induces TFEB expression and enhances autophagy, improving insulin sensitivity and reducing hepatic steatosis in animal models, although clinical benefits in humans have thus far been modest.^156^ Spermidine, a polyamine that enhances autophagic flux, improves insulin resistance and gut health in preclinical models, although its translational potential remains limited by the lack of robust human data.^157^ At the level of mitophagy, compounds such as docosahexaenoic acid (DHA) reduce lipid accumulation in adipocytes and improve mitochondrial function, supporting their potential utility in obesity management.^158^ Sesamol similarly promotes adipocyte browning and enhances lipid metabolism through mitophagy regulation.

In pancreatic β-cells, mitophagy-inducing compounds such as silibinin and cyanidin-3-O-glucoside (C3G) improve glucose metabolism and alleviate hyperglycemia.^159^ C3G has additionally demonstrated efficacy in reducing hepatic steatosis in MASLD mouse models.^105^ Other natural compounds, including quercetin and Akebia saponin D, ameliorate lipid accumulation and hepatic steatosis in preclinical MASLD models by targeting mitophagy,^103,160,161^ reinforcing the concept that mitochondrial quality control is a critical determinant of metabolic tissue health.

##### Targeting lipophagy in MASLD

Multiple compounds, including MSDC-0602K, PF-05221304, IVA337, saroglitazar, and curcumin, modulate mTORC1 signaling to restore lipophagic flux and are progressing through phase II clinical trials (Tables 1 and 2). Transcription factor modulators such as elafibranor, a PPAR ligand, have advanced to phase III trials and demonstrated efficacy in improving lipid handling and reducing hepatic steatosis.^162^ These studies underscore the therapeutic potential of restoring coordinated autophagy-mitophagy activity in metabolic liver disease.

##### Drug repurposing

Repurposing established metabolic drugs further supports this integrated strategy. Metformin enhances mitophagy and mitochondrial function, contributing to improved glucose and lipid metabolism.^103^ Liraglutide similarly ameliorates MASH by enhancing mitophagy in animal models,^163^ highlighting the dual autophagy-mitophagy actions of clinically approved agents.

##### Non-pharmacological interventions

Non-pharmacological interventions remain among the most potent inducers of autophagy and mitophagy. Fasting, calorie restriction, and exercise robustly activate these pathways and confer broad metabolic benefits. Clinical studies have demonstrated exercise-induced increases in autophagy markers in human skeletal muscle,^164^ while a randomized controlled trial in MASLD patients showed that intermittent fasting combined with aerobic exercise significantly reduced hepatic steatosis, body weight, and fat mass while improving insulin sensitivity.^165,166^ Exercise regimens incorporating mixed modalities, such as swimming and interval training, further enhance mitophagy, reducing oxidative stress, apoptosis, and inflammatory signaling in preclinical models.^167^

##### Lifestyle interventions and mitophagy

Exercise interventions, particularly those combining multiple types, such as swimming and interval training, have demonstrated significant potential in enhancing mitophagy in animal models.^167^ By regulating mitophagy, exercise can inhibit oxidative stress, apoptosis, and inflammatory responses, offering a non-pharmacological approach to improving metabolic health.

##### Emerging technologies

Emerging technologies offer unprecedented precision in targeting the autophagy-mitophagy axis. Autophagy-targeting chimeras (AUTACs) selectively label damaged mitochondria for degradation through mechanisms distinct from the PINK1-Parkin pathway (Fig. 1a), enabling controlled induction of mitophagy.^103,168^ Such approaches represent a promising frontier for therapeutic intervention in complex metabolic diseases characterized by mitochondrial dysfunction.

#### UPS-targeted therapies in metabolic diseases

##### Targeting E3 ligases in metabolic diseases

Targeting E3 ubiquitin ligases has demonstrated particular promise. MID-00935 disrupts the interaction between MG53 and IRS1, thereby improving insulin sensitivity and glucose homeostasis in insulin-resistant mouse models.^169^ Beyond insulin signaling, E3 ligases such as TRIM31 and Gp78 regulate lipid metabolism and inflammation, and their modulation reduces hepatic steatosis, offering targeted strategies for MASLD.^115^

##### Targeting DUBs

DUBs represent another important target class. USP20 inhibitors, including GSK2643953A, reduce obesity, improve glucose tolerance, and alleviate hepatic lipid accumulation in high-fat-diet mouse models.^127^ USP1 inhibitors, such as ML323 and SJB-043, protect against β-cell apoptosis and hyperglycemia under diabetic conditions.^170,171^

##### PROTACs

Although PROTAC technology has been primarily developed in oncology, early studies suggest potential applications in metabolic disease. PROTAC degraders 245 and 246 selectively target HMGCR, a key enzyme in cholesterol biosynthesis, achieving efficient degradation in preclinical models.^172–174^ This represents a major advance as the first successful PROTAC-mediated degradation of an ER-localized, polytopic transmembrane protein, opening new possibilities for metabolic intervention.

#### Therapeutic targeting of ERAD and ER stress in metabolic diseases

##### 4-Phenylbutyric Acid (4-PBA)

Chemical chaperones such as 4-PBA have demonstrated beneficial effects in mouse models of obesity and diabetes, normalizing hyperglycemia and restoring systemic insulin sensitivity.^175^ 4-PBA also alleviates hepatic steatosis, MASLD, and MASH in preclinical studies.^175,176^

##### Tauroursodeoxycholic Acid (TUDCA)

TUDCA, a bile acid derivative, similarly improves glucose homeostasis, enhances insulin sensitivity, and reduces hepatic steatosis in obese and diabetic mouse models.^175^ These effects are attributed to its ability to reduce ER stress, improve protein folding capacity, and restore cellular function in metabolic tissues.

In conclusion, the intricate interplay between metabolic diseases and protein homeostasis underscores the pivotal role of proteostasis networks, including the UPS, autophagy, and the UPR, in the pathogenesis of conditions such as obesity, T2DM, and MASLD (Fig. 3). The preclinical success of chemical chaperones such as 4-PBA and TUDCA illustrates the potential of targeting ER stress and the ERAD system in metabolic diseases. While these compounds are not direct ERAD modulators, their success highlights the importance of targeting ER stress in metabolic diseases. Future research should focus on developing direct ERAD-targeting therapies and optimizing existing approaches to translate these findings into clinical applications.

## Cancer and protein homeostasis

Proteostasis is a fundamental cellular process that ensures proper synthesis, folding, trafficking, and degradation of proteins, maintaining cellular function and viability. In cancer, the delicate balance of proteostasis is often disrupted, leading to the accumulation of misfolded or damaged proteins, aberrant signaling pathways, and genomic instability, all of which contribute to tumorigenesis and cancer progression.

### The role of HSPs in cancer

HSPs are a family of molecular chaperones that play a central role in cancer biology. Their overexpression in cancer supports critical processes such as tumor growth, survival, metastasis, and resistance to therapy. By stabilizing oncogenic proteins, buffering cellular stress, and facilitating adaptation to hostile microenvironments, characterized by hypoxia, oxidative stress, and nutrient deprivation, HSPs enable cancer cells to thrive under conditions that would otherwise be lethal. This multifaceted role of HSPs makes them key players in tumorigenesis, progression, and therapeutic resistance across a wide range of cancers.

#### HSP27 and HSP40 in tumorigenesis, EMT, and metastatic signaling

##### HSP27 is a key regulator of tumorigenesis and metastasis

HSP27-mediated regulation of the Hippo pathway has been observed in various cancers, including prostate, breast, and lung tumors.^177^ HSP27 also facilitates STAT3 phosphorylation, either through IL-6 signaling or independently, leading to STAT3 nuclear translocation and its binding to the TWIST promoter. This process enhances epithelial-to-mesenchymal transition (EMT), a critical step in cancer metastasis.^178^ The HSP27-driven activation of the IL-6/STAT3/TWIST signaling axis has been demonstrated in multiple bladder cancer cell lines, where it promotes EMT and invasiveness.^178^ Overexpression of HSP27 is strongly associated with tumorigenesis, metastasis, and poor prognosis in various cancers, including head and neck squamous cell carcinoma, pediatric acute myeloid leukemia, breast cancer, and colorectal cancer.

##### HSP40 is a family of chaperones with diverse roles in cancer

The HSP40 family, also known as DNAJ proteins, is frequently overexpressed in cancers such as colorectal, gastric, and lung cancers, particularly in cases with lymph node and distant organ metastasis.^179,180^ Among these, DNAJ member A1 (DnaJA1) plays a critical role in colorectal cancer by promoting cell cycle progression. DnaJA1 is transcribed by E2F transcription factor 1 and prevents the ubiquitin-mediated degradation of CDC45, a key regulator of DNA replication.^181^ Elevated DNAJC12 levels are associated with higher morbidity and mortality, underscoring its potential as a therapeutic target.^179^

#### HSP60 and HSP70 in mitochondrial control, stress adaptation, and immune modulation

##### HSP60 plays dual role in tumor promotion and suppression

HSP60 exhibits context-dependent roles in cancer, acting as either a tumor promoter or suppressor depending on the malignancy type. In ovarian cancer, elevated HSP60 expression supports tumor progression by stabilizing mitochondrial homeostasis and activating the mTOR signaling pathway. Conversely, in glioblastoma, inhibition of HSP60 increases mitochondrial ROS generation, triggering AMPK activation and suppressing mTORC1-mediated phosphorylation of S6K and 4EBP1. This deactivates the protein translation machinery, reducing cancer growth. HSP60 also modulates the folding and function of TRiC client proteins, including STAT3, cyclins B and E, p53, and Von Hippel‒Lindau (VHL) protein.^182^ Dysregulated STAT3 activity, driven by HSP60, promotes oncogenic transcriptional programs that enhance cell survival, proliferation, and angiogenesis.^182^

##### HSP70 is a central player in cancer survival and metastasis

HSP70 proteins are pivotal in maintaining oncogenic signaling and cellular survival. HSP72, for instance, stabilizes kinetochore-associated microtubules in amplified centrosomes, a phenotype unique to cancer cells. Without this stabilization, mitotic catastrophe and apoptosis are triggered, highlighting the role of HSP72 in cancer-specific mitotic signaling.^183^ HSP70B promotes breast cancer metastasis by upregulating mesenchymal markers such as N-cadherin, MMP2, SNAIL, and vimentin, facilitating EMT.^184^ HSP70 also suppresses apoptosis and oncogene-induced senescence, further contributing to cancer progression.^185^ Ironically, HSP70 can act as a damage-associated molecular pattern (DAMP), eliciting anti-tumor immune responses. However, prolonged exposure to extracellular HSP70 can result in immune tolerance, complicating its role in cancer immunity.^186^ HSP70 also negatively regulates the NLRP3 inflammasome, a key protein complex involved in inflammation.^187^ Extracellular HSP70 forms activation complexes with co-chaperones such as HSP90α, Hop, and HSP40, enhancing breast cancer cell migration and invasion by increasing MMP2 activity.^188^

#### HSP90 as a master regulator of oncogenic signaling networks

HSP90 is frequently overexpressed in cancers such as cholangiocarcinoma, lung cancer, gastric cancer, breast cancer, and glioblastoma, and its elevated levels are often associated with poor prognosis.^189^ HSP90 also promotes the activation of oncogenic protein kinases such as JAK2/STAT3, PI3K/AKT, and MAPK, facilitating cancer progression.^190^ Additionally, HSP90 activates HIF-1α and NF-kB, driving critical oncogenic processes such as EMT, invasion, motility, and metastasis.^191^ HSP90 interacts with and stabilizes HMGCR, the rate-limiting enzyme in the mevalonate pathway, which is crucial for cancer progression. HSP90 also promotes tumor angiogenesis by enhancing the transcription and expression of vascular endothelial growth factor receptors (VEGFRs), which are essential for endothelial cell-dependent angiogenesis.^192^ In prostate cancer, HSP90 stabilizes the androgen receptor, enhancing its affinity for androgen and supporting tumor formation.^188,193^

#### HSPs in chemotherapy and radiotherapy resistance

##### HSPs in chemotherapy resistance

HSPs play a critical role in mediating resistance to chemotherapy and radiotherapy. Elevated levels of HSP27 and HSP70 in breast cancer cell lines are associated with increased resistance to doxorubicin, independent of P-glycoprotein (P-gp), a key mediator of multidrug resistance (MDR).^194^ In colorectal cancer, HSP27 contributes to resistance against 5-fluorouracil (5-FU), gemcitabine, paclitaxel, and temozolomide. Silencing HSP27 with siRNA mitigates this resistance, highlighting its role in chemotherapeutic resistance mechanisms.^188,195^

HSP40 hypo-expression has been observed in ovarian cancer lesions resistant to paclitaxel, topotecan, and cisplatin.^196^ HSP60 is highly expressed in oxaliplatin- and cisplatin-resistant ovarian and bladder cancer cells, and inhibiting HSP60 enhances the drug sensitivity of 5-FU-resistant colorectal cancer cells.^197^ HSP70 is associated with cisplatin resistance in ovarian, lung, and osteosarcoma cancers, where it disrupts the mitochondrial apoptotic cascade and maintains cell cycle progression.^198,199^ GRP78 (HSP70) contributes to 5-FU resistance in colorectal and ovarian cancer cells by modulating the PI3K/AKT/mTOR and c-Src/LSF/TS signaling pathways.

HSP90 expression is often induced by chemotherapeutic agents such as doxorubicin, cisplatin, and methotrexate.^200^ HSP90 also regulates the expression of drug resistance genes, including LRP, GST-π, p53, Bcl-2, survivin, ERCC1, XRCC1, and BRCA1/2.^201^ In breast cancer, elevated HSP90 levels stabilize estrogen receptor (ErbB)-dependent PI3K/AKT and ERK signaling pathways, counteracting the effects of hormonal therapies such as fulvestrant.^202^

##### HSPs in radiotherapy resistance

HSPs are also implicated in resistance to radiotherapy. Elevated HSP70 expression in lung, breast, tongue, and gingival cancers contributes to radiotherapy resistance by facilitating proper protein folding and protecting against radiation-induced damage.^203^ HSP90-mediated signaling helps cancer cells resist radiation by stabilizing oncogenic proteins and promoting DNA repair, cell cycle regulation, and survival signaling.^204^ This stabilization of proteins such as mutated p53 enhances the ability of cancer cells to repair radiation-induced DNA damage, reducing cell death following radiotherapy.

### The role of autophagy and mitophagy in cancer

Autophagy and mitophagy are deeply intertwined with cancer biology, acting as context-dependent regulators that can either suppress tumor initiation or promote tumor survival and progression depending on tumor stage, metabolic state, and microenvironmental stressors. This duality reflects the complexity of cellular quality control pathways in cancer and underscores their significance as both pathogenic drivers and therapeutic targets.

#### Autophagy and mitophagy as early barriers to tumor initiation and genomic instability

In early tumorigenesis, autophagy functions predominantly as a tumor-suppressive mechanism by removing damaged organelles, misfolded proteins, and oncogenic substrates such as p62, thereby preventing genomic instability and malignant transformation.^205^ Loss of key autophagy genes, such as BECN1 and ATG5, predisposes mice to diverse cancers, including lung adenocarcinoma, B-cell lymphoma, and hepatocellular carcinoma,^206,207^ highlighting the essential role of autophagy in maintaining cellular integrity. Failure of autophagy in this context promotes the accumulation of oxidative stress, aberrant signaling, mitochondrial damage, and chronic inflammation, collectively establishing a pro-tumorigenic cellular environment.

Mitophagy contributes to this tumor-suppressive function by maintaining mitochondrial quality control (MQC). Dysfunctional mitochondria generate excessive ROS, mtDNA instability, and oncogenic signaling. Mutations in mitochondrial DNA (mtDNA), commonly detected across cancers such as renal oncocytoma, gastric adenocarcinoma, renal cell carcinoma, bladder cancer, ovarian cancer, thyroid cancer, and pancreatic cancer,^208–215^ can disrupt OXPHOS, enhance ROS production, and promote tumorigenesis. Some mutations accelerate intestinal tumor formation,^210^ while MT-CYTB and MT-CO1 variants enhance tumor growth through ROS-induced NF-κB2 activation.^211–213^ Interestingly, selective pressure against truncating mutations in complex V suggests that mitochondrial function remains necessary for survival.^216^ Thus, both autophagy and mitophagy restrain early tumorigenic events by eliminating damaged mitochondria, suppressing oxidative stress, and stabilizing metabolic equilibrium.

#### Autophagy- and mitophagy-driven metabolic adaptation in established tumors

Once tumors are established, the role of autophagy frequently shifts toward tumor promotion. Autophagy provides metabolic substrates through recycling pathways, helping cancer cells survive under nutrient deprivation, hypoxia, immune stress, and therapeutic challenge.^217,218^ It supports high biosynthetic and energetic demands of rapidly proliferating cells and may facilitate dormancy of residual tumor cells following chemotherapy or radiation, contributing to recurrence and metastasis.^219^ Hypoxia, a defining feature of solid tumors, potently induces autophagy through both HIF-dependent and HIF-independent mechanisms.^220^ HIF-1 interacts with BCL2, BNIP3, BNIP3L/NIX, Beclin-1, ATG proteins, and PIK3C3 to stimulate autophagy,^221,222^ while AMPK and PTEN suppress mTORC1, activating ULK1 and enhancing autophagosome-lysosome fusion.^223^ Under hypoxic pressure, autophagy orchestrates selective degradation of cellular components, including mitochondria (mitophagy), ER (ER-phagy), lipid droplets (lipophagy), ribosomes (ribophagy), nuclei (nucleophagy), and peroxisomes (pexophagy),^224–227^ thereby sustaining tumor metabolism and viability.

Mitophagy is a crucial contributor to this adaptive response. The PINK1-Parkin pathway, frequently altered in cancers such as lung, breast, and glioma,^228^ governs mitochondrial turnover, iron homeostasis, and metabolic flexibility. Parkin deficiency promotes spontaneous hepatocellular carcinoma by enhancing hepatocyte proliferation and apoptosis resistance.^229^ The pathway controls the mitochondrial iron importers SLC25A37 and SLC25A28^230^; pathway failure drives iron accumulation, HIF1α stabilization, inflammasome activation, and tumor progression.^231^ Loss of PINK1-Parkin exacerbates KRAS-driven pancreatic tumorigenesis,^230^ whereas PINK1 acts as a tumor suppressor in glioblastoma by reducing ROS and HIF activation.^232^ Conversely, in lung and esophageal cancers, PINK1-driven mitophagy promotes proliferation and chemoresistance. These observations illustrate cancer type- and context-dependent consequences of mitophagy modulation.

Mitochondrial dynamics further intersect with mitophagy regulation. Enhanced mitochondrial fission, frequently observed in tumors, supports glycolysis, invasiveness, and resistance to apoptosis,^233^ while impaired fusion accentuates mitochondrial and metabolic instability. Mitochondrial chaperonins such as TRAP-1 also shape mitochondrial proteostasis and tumor metabolism. TRAP-1 supports tumor growth by promoting glycolysis, inhibiting succinate dehydrogenase, and activating cytochrome-c oxidase,^234–236^ although reduced TRAP-1 expression at later stages has been linked to enhanced motility and invasion, suggesting context-dependent roles.^237,238^

#### Autophagy, mitophagy, and therapy resistance

Autophagy and mitophagy critically influence therapeutic resistance. In chronic myeloid leukemia, imatinib resistance is associated with autophagy activation in leukemic stem cells; combined autophagy inhibition enhances cell death and eradicates resistant populations.^239^ Elevated expression of ATG genes, including ATG4B, ATG4D, ATG5, and BECN1, underscores its mechanistic relevance,^240^ and ATG4B knockdown sensitizes cells to therapy. Similarly, in glioblastoma stem-like cells, autophagy inversely correlates with the response to temozolomide, and its inhibition improves treatment efficacy.^241^ Parallel resistance mechanisms are increasingly attributed to mitophagy-mediated mitochondrial preservation and metabolic resilience, reinforcing the therapeutic relevance of targeting these pathways.

### The role of the UPS in cancer

Dysregulation of the UPS has been implicated in the development, progression, and treatment resistance of cancer. Carcinogenesis is marked by genetic and epigenetic alterations that disrupt normal cellular functions, many of which are controlled by UPS-mediated protein degradation. This dysregulation contributes to the loss of tumor suppressor functions, the stabilization of oncogenes, and the modulation of key signaling pathways that promote cancer cell proliferation.^242^ Imbalances in UPS components, such as the overactivation of E3 ligases or inhibition of DUBs, further exacerbate these processes, driving tumorigenic characteristics.

#### UPS-mediated control of oncogenic signaling pathways in solid tumors

The UPS plays a significant role in the development of skin cancer, particularly through its regulation of apoptotic pathways and the TGF-β signaling cascade.^243^ TGF-β signaling has a dual role in cancer, acting as a tumor suppressor in early stages and promoting invasion and metastasis in advanced malignancies.^244^ The majority of TGF-β pathway components, including TβRI, TβRII, and SMAD proteins, are regulated by proteasomal degradation.^245^ E3 ligases from the NEDD4 family, such as NEDD4-2, SMURF1, SMURF2, and WWP1, are key regulators of this pathway.^246^ For example, SMAD2 ubiquitination by Itch/AIP4 promotes its phosphorylation by TβRI, facilitating signal transduction.^247^ Mutations in SMAD2/4 or their receptors, which are prone to ubiquitination, disrupt TGF-β signaling and contribute to tumorigenesis.^248^ These mutations are more prone to ubiquitination, leading to an imbalanced TGF-β pathway and thus to tumorigenesis. In melanoma, receptor-interacting protein 1 (RIP1) is often upregulated and promotes cancer cell proliferation by activating NF-κB.^249^ Ubiquitination of RIP1 reduces NF-κB activity and inhibits melanoma growth, underscoring the role of the UPS in regulating oncogenic signaling pathways.^250^ Environmental factors, such as chemicals or radiation, can also disrupt the UPS balance. Low-LET ionizing radiation, for instance, inhibits proteasomal activity and induces free-radical damage to UPS-associated molecules, increasing the risk of tumorigenesis.^251,252^

Elevated proteasomal activity is frequently observed in colorectal cancer (CRC), where deregulation of specific 20S proteasome subunits has been linked to carcinogenesis.^253^ A pivotal event in CRC development is the accumulation of oncogenic β-catenin due to disruptions in its regulatory pathways.^254^ β-catenin degradation via the UPS occurs through either the APC-dependent p53/Siah1 pathway or the APC-independent retinoid X receptor α (RXRα) pathway.^255^ Siah1, a critical link between p53 and Wnt/β-catenin signaling, regulates β-catenin turnover independently of GSK3β, and its dysfunction contributes to CRC progression.^255^

#### UPS regulation of cell cycle progression and metabolic reprogramming

The UPS tightly controls cell cycle regulators, such as cyclins and cyclin-dependent kinase (CDK) inhibitors, whose dysregulation is common in cancer. Cyclin E, for example, is degraded by the SCF ubiquitin ligase complex, which uses the F-box protein Fbw7 to recognize and ubiquitinate cyclin E.^256^ Mutations in Fbw7, observed in breast and ovarian cancers, lead to cyclin E accumulation, premature S-phase entry, chromosomal instability, and tumor formation.^257,258^ Similarly, p27Kip-1, a CDK inhibitor, is regulated by ubiquitin-mediated degradation. Reduced p27Kip-1 levels, often due to overexpression of the F-box protein Skp2, are associated with tumor progression and poor prognosis in various cancers.^259^

Cancer cells exhibit a metabolic shift from OXPHOS to glycolysis, known as the Warburg effect. This reprogramming is driven by alterations in glycolytic enzymes regulated by the UPS. Hexokinase 2 (HK2), which catalyzes the first step of glycolysis, is stabilized by the deubiquitinase CSN5 in HCC, promoting metastasis.^260^ Conversely, TRAF6-mediated ubiquitination of HK2 facilitates its degradation via autophagy, reducing glycolytic activity.^261^ Pyruvate kinase M2 (PKM2), another key glycolytic enzyme, is stabilized by the deubiquitinase USP20, making USP20 a potential therapeutic target.^262^

#### UPS-driven mechanisms of metastasis and therapy resistance

The UPS also plays a critical role in cancer metastasis and treatment resistance. The ubiquitin-conjugating enzyme E2N (UBE2N) is overexpressed in various cancers and promotes breast cancer metastasis to the lung by activating TGFβ-mediated signaling pathways.^263^ In castration-resistant prostate cancer, the UPS supports androgen receptor (AR) function by facilitating its nuclear localization and stabilizing mutated AR proteins, enabling tumor cells to survive and proliferate under low androgen levels. Additionally, the UPS contributes to treatment resistance by degrading apoptosis-promoting factors and enhancing pro-survival signaling pathways, such as NF-κB and PI3K/AKT.^264^

### The role of ERAD and UPR in cancer

#### The UPR as a stress-adaptive survival network in tumor cells

The UPR is frequently upregulated in cancer, reflecting its central role in enabling tumor cells to cope with sustained proteotoxic stress. Rapid proliferation, high protein synthesis rates, hypoxia, nutrient deprivation, acidosis, and oxidative stress collectively impose a severe folding burden on the ER. By enhancing ER protein-folding capacity, reducing the accumulation of misfolded proteins, and activating adaptive signaling programs, the UPR allows cancer cells to survive and proliferate in hostile microenvironments that would otherwise compromise cellular viability. This adaptive capacity also contributes to resistance against a wide range of anticancer therapies, many of which further exacerbate ER stress. Importantly, the UPR does not function as a unidirectional survival pathway. When ER stress is excessive or prolonged beyond the adaptive threshold, UPR signaling can shift from cytoprotective to pro-apoptotic outcomes, frequently through crosstalk with mitochondrial death pathways. This critical balance between survival and cell death is orchestrated by the coordinated activity of the three canonical UPR branches, IRE1, PERK, and ATF6, which together determine cell fate decisions under stress. Despite extensive investigation, the precise molecular determinants governing the transition from adaptive UPR signaling to apoptosis remain incompletely understood. This knowledge gap represents a major challenge for therapeutic strategies aimed at selectively targeting UPR signaling in cancer cells without inducing unacceptable toxicity in normal tissues.^265,266^

#### PERK-eIF2α-ATF4 signaling in angiogenesis, EMT, and metastatic progression

Persistent activation of the cytoprotective arm of the UPR can actively drive tumorigenesis and malignant progression. Among the UPR branches, the PERK-eIF2α-ATF4 signaling axis has emerged as a particularly influential regulator of cancer cell adaptation to stress. PERK activation enables tumor cells to transiently attenuate global protein synthesis while selectively enhancing the translation of stress-responsive genes that promote survival, invasion, and metabolic rewiring. One of the most prominent tumor-promoting consequences of PERK activation is its ability to stimulate angiogenesis. The PERK-ATF4 pathway induces the expression of multiple vascular endothelial growth factors, including VEGFA, VEGFB, VEGFC, VEGFD, VEGFF, and placental growth factor, which collectively support endothelial cell survival, neovascularization, and sustained tumor growth.^267^ In parallel, PERK-ATF4 signaling is tightly linked to EMT, a transcriptional reprogramming process that enables epithelial cancer cells to acquire migratory, invasive, and stem-like properties. Through EMT induction, PERK signaling directly contributes to metastatic dissemination and colonization of distant organs. Thus, sustained PERK pathway activation integrates proteostasis control with angiogenic and invasive programs, positioning this UPR branch as a central driver of tumor progression rather than a mere stress-response mechanism.

#### UPR-driven tumor dormancy and therapeutic resistance

Beyond its role in active tumor growth, the UPR plays a pivotal role in cancer cell dormancy, long-term survival, and resistance to anticancer therapies. Under therapeutic pressure, cancer cells frequently exploit UPR signaling to enter a quiescent or dormant state characterized by reduced proliferation and heightened stress tolerance. This adaptive dormancy allows tumor cells to evade cytotoxic treatments and persist as minimal residual disease until conditions favor reactivation and relapse. Within this context, PERK signaling again emerges as a key determinant of therapy resistance. Sustained activation of the PERK-eIF2α-ATF4 pathway has been strongly associated with EMT induction, survival under chemotherapeutic stress, and metastatic competence.^268^ Genetic studies further highlight the complexity of PERK signaling in cancer: deletion of PERK reduces Neu-dependent mammary tumor development and lung metastasis, underscoring its role in tumor progression. However, prolonged or complete PERK inactivation can paradoxically increase genomic instability, thereby predisposing cells to tumorigenesis under certain conditions.^269^ These findings illustrate that PERK signaling exerts dose-, duration-, and context-dependent effects, complicating efforts to therapeutically manipulate this pathway.

#### IRE1/XBP1 signaling in tumor growth, angiogenesis, and chemoresistance

In parallel to PERK, the IRE1/XBP1 branch of the UPR plays a critical role in sustaining tumor growth and mediating resistance to therapy. Upon activation, IRE1 catalyzes the unconventional splicing of XBP1 mRNA, generating the transcriptionally active XBP1 spliced isoform (XBP1s). XBP1s drives the expression of genes involved in ER protein folding, ER-associated degradation (ERAD), lipid biosynthesis, and stress adaptation, thereby enhancing tumor cell fitness under proteotoxic conditions. In estrogen receptor-positive breast cancer, XBP1s directly regulates the transcription of nuclear receptor coactivator 3 (NCOA3), linking UPR signaling to hormone receptor activity and therapy resistance.^270^ In pancreatic adenocarcinoma, XBP1s promotes angiogenesis through VEGF-independent mechanisms, further underscoring its capacity to sustain tumor vascularization and growth.^271^ Moreover, overexpression of XBP1s pushes plasma cells toward a premalignant state, while genetic or functional XBP1 deficiency suppresses tumor growth and angiogenesis.^272^ Collectively, these observations establish the IRE1/XBP1 pathway as a central regulator of tumor progression, metabolic adaptation, and chemoresistance.

Taken together, these elaborate mechanisms highlight the UPR not as a uniform survival pathway but as a dynamic, branch-specific signaling network whose outputs depend on tumor type, microenvironment, and stress duration. The intertwined roles of PERK and IRE1 in angiogenesis, EMT, dormancy, and therapy resistance emphasize why indiscriminate UPR inhibition is unlikely to succeed clinically and why pathway-selective, context-aware strategies are needed.

### Therapeutic targeting of proteostasis in cancer

**Fig. 4:**
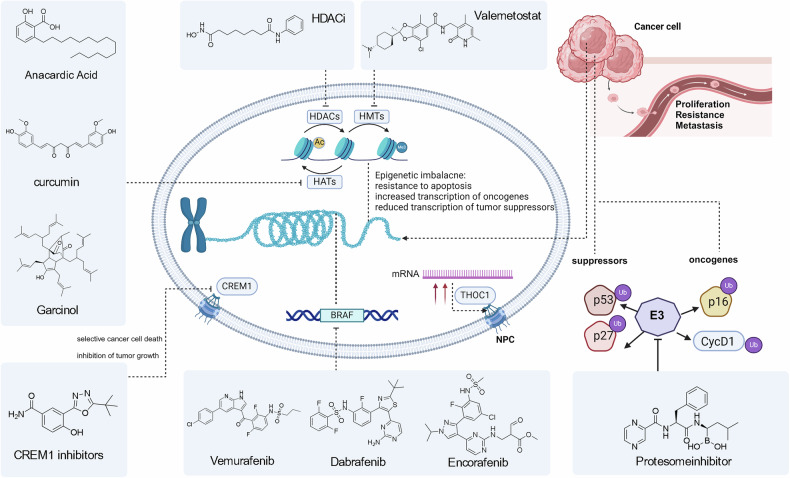
Therapeutic targeting of proteostasis, epigenetic regulation, and protein degradation pathways in cancer. This figure illustrates various drug delivery mechanisms targeting cancer cells by modulating epigenetic regulation, oncogenic signaling, and protein degradation pathways. At the center, epigenetic modifications play a critical role in cancer progression. Histone deacetylases (HDACs) and histone methyltransferases (HMTs) contribute to an epigenetic imbalance that leads to resistance to apoptosis, increased oncogene transcription, and decreased tumor suppressor transcription. Histone acetyltransferases (HATs) counteract these effects, and natural compounds such as anacardic acid, curcumin, and garcinol act as HAT activators to restore epigenetic balance. HDAC inhibitors (HDACis) and valemetostat (a dual EZH1/2 inhibitor) target HDACs and HMTs, respectively, to counteract oncogenic epigenetic modifications. In the genomic regulation pathway, CREM1 is involved in chromatin remodeling, and its inhibitors selectively induce cancer cell death and inhibit tumor growth. Additionally, oncogenic BRAF mutations drive tumor progression, and targeted inhibitors such as vemurafenib, dabrafenib, and encorafenib block its activity. THOC1, part of the nuclear pore complex (NPC), facilitates mRNA export, leading to increased expression of oncogenes. Proteasome degradation pathways also contribute to cancer progression. The E3 ubiquitin ligase complex tags tumor suppressors (p53, p27) for degradation while promoting oncogene stability (p16, Cyclin D1). Proteasome inhibitors counteract this degradation process, restoring tumor suppressor function and preventing oncogenic protein accumulation. The schematic in the top right highlights the cancer cell phenotype, characterized by uncontrolled proliferation, resistance to therapy, and metastasis. Targeting these pathways with specific drug classes offers promising therapeutic strategies to combat cancer progression. This figure was created at Biorender.com

Cancer cells are exposed to persistent proteotoxic stress driven by rapid proliferation, genomic instability, hypoxia, metabolic rewiring, and therapeutic pressure. To survive under these conditions, malignant cells extensively reprogram proteostasis networks. This dependency creates therapeutic vulnerabilities that can be exploited to selectively impair tumor survival, overcome drug resistance, and enhance sensitivity to conventional therapies (Fig. 4).

#### Targeting heat shock proteins in cancer

##### Targeting HSP27

Targeting HSP27 has shown promise through agents such as RP101 (bromovinyldeoxyuridine), a nucleoside antiviral compound that binds HSP27 and disrupts its interaction with client proteins. This interference sensitizes tumor cells to chemotherapy and improves treatment efficacy in preclinical cancer models, positioning RP101 as a candidate for combination therapy approaches.^273^

##### Targeting HSP60

The mitochondrial chaperone HSP60 has also emerged as a target in cancer therapy. Myrtucommulone directly interacts with HSP60, inducing apoptosis through mitochondrial dysfunction and highlighting a mechanism that links proteostasis disruption to intrinsic cell death pathways.^274^ Similarly, sinularin decreases HSP60 expression in melanoma cells, activating caspase-dependent apoptosis and suppressing tumor growth.^275^

##### Targeting HSP70

HSP70 targeting has yielded several mechanistically distinct anticancer strategies. The natural flavonoid fisetin induces apoptosis in HCT-116 colon cancer cells by inhibiting HSF1 binding to the HSP70 and BAG3 promoters, thereby suppressing the heat shock response and promoting cell death.^276^ Pifithrin-μ (PES), an HSP70 inhibitor, arrests non-small cell lung cancer cells in the G0/G1 phase and promotes expression of death receptors DR4 and DR5. However, concerns have emerged regarding PES-induced ROS accumulation, which may enhance metastatic potential in surviving cells.^277^ Apoptozole, another HSP70 inhibitor, promotes cancer apoptosis by inducing lysosomal membrane permeabilization, further validating HSP70 as a therapeutic target.^278^

##### Targeting HSP90

HSP90 inhibition remains one of the most clinically advanced chaperone-targeting strategies. Ganetespib acts as a radiosensitizer in pancreatic ductal adenocarcinoma by disrupting HIF-1α, STAT3, and AKT signaling and inducing G2-M cell cycle arrest, thereby impairing DNA damage repair during irradiation.^279,280^ PU-H71 suppresses cell survival and promotes DNA damage accumulation by inhibiting RAD51 and Ku70 expression and is currently under clinical evaluation, including in combination with carbon-ion radiotherapy.^281^ Combination strategies have proven particularly effective; co-administration of NVP-AUY922 with the BCL-2 family inhibitor ABT-737 overcomes resistance mediated by MCL-1 in small-cell lung cancer.^282^

#### Therapeutic modulation of the autophagy-mitophagy axis in cancer

##### Autophagy inhibition in cancer therapy

Autophagy inhibition has been extensively explored in oncology. CQ and HCQ, lysosomal deacidifying agents originally developed for malaria and autoimmune diseases, inhibit autophagy by blocking autophagosome-lysosome fusion. CQ induces apoptosis-resistant tumor cell death in Myc-driven lymphoma models^283^ and enhances the efficacy of multiple anticancer therapies. Combination regimens with CQ/HCQ improve outcomes when paired with radiation therapy in brain metastases,^284,285^ HDAC inhibitors such as vorinostat,^286^ mTOR inhibitors such as temsirolimus,^287^ temozolomide,^288^ proteasome inhibitors such as bortezomib,^289^ gemcitabine in pancreatic ductal adenocarcinoma,^290^ and erlotinib in non-small cell lung cancer (Fig. 4).^291^ However, CQ/HCQ efficacy varies with tumor microenvironment pH; acidic conditions limit drug uptake in breast cancer,^292^ whereas in bladder cancer, CQ disrupts cholesterol metabolism and enhances cytotoxicity.^293^

Targeting upstream autophagy regulators has further refined therapeutic approaches. ULK1 inhibition with SBI-0206965 sensitizes cancer cells to mTOR inhibitors and disrupts AMPK-mediated survival signaling.^294,295^ In non-small cell lung cancer, ULK1 inhibition enhances cisplatin sensitivity, while in glioblastoma, it overcomes temozolomide resistance when combined with high-dose treatment.^296^ ULK1 overexpression also confers resistance in clear-cell renal carcinoma, reinforcing its relevance as a therapeutic target.^297^ Inhibition of PIK3C3/VPS34 with compounds such as SB02024 and SAR405 enhances the efficacy of sunitinib in breast cancer models.^298^

##### Autophagy induction in cancer therapy

Conversely, autophagy induction can also promote tumor cell death in specific contexts. BH3 mimetics such as ABT-737 disrupt Bcl-2/Beclin-1 interactions, enhancing Beclin-1-dependent autophagy.^299^ The pan-BH3 mimetic (-)-gossypol induces autophagy-like cell death in glioblastoma through autophagosome formation, lysosomal damage, and cytochrome c release. Obatoclax mesylate (GX15-070), which targets multiple Bcl-2 family proteins, including Mcl-1, has demonstrated anticancer activity in clinical trials for hematologic malignancies and induces both autophagy and apoptosis.^300^ Natural compounds such as curcumin also induce autophagy in gastric cancer cells, marked by increased Beclin-1, Atg3, Atg5, and LC3-II expression.^299^

##### Mitophagy as a survival mechanism and therapeutic target

Mitophagy represents a critical adaptive mechanism in cancer cells exposed to mitochondrial stress. Chemotherapeutic agents such as cisplatin induce mitochondrial damage and ROS production, triggering mitophagy as a survival response.^301^ Suppression of mitophagy can therefore restore chemosensitivity. In cervical cancer, melatonin inhibits JNK- and Parkin-mediated mitophagy, restoring cisplatin-induced apoptosis.^302^ In hepatic carcinoma, mitophagy inhibition with Mdivi-1 or bafilomycin A enhances cisplatin efficacy.^303^

##### Targeting mitochondrial metabolism

Targeting mitochondrial metabolism further intersects with mitophagy modulation. IACS-010759, a selective mitochondrial complex I inhibitor, reduces tumorigenicity in acute myeloid leukemia models dependent on oxidative phosphorylation, with enhanced efficacy under glucose-restricted conditions.^304,305^ Metabolic interventions such as ketogenic diets limit glucose availability and suppress anabolic growth, particularly when combined with chemotherapy.^306^ Combination strategies using α-lipoic acid and hydroxycitrate enhance catabolic metabolism and suppress tumor growth in glioblastoma, brain metastases, and lung cancer.^307^

#### UPS-targeted therapies in cancer

##### Proteasome inhibitors

The ubiquitin‒proteasome system has become one of the most successfully exploited proteostasis pathways in oncology (Fig. 4). The FDA approval of bortezomib in 2003 for multiple myeloma marked a paradigm shift in targeted cancer therapy. Despite its clinical success, bortezomib is limited by acquired resistance and adverse effects, including peripheral neuropathy and hematologic toxicity.^308^ Second-generation proteasome inhibitors, including carfilzomib, ixazomib, oprozomib, delanzomib, and marizomib, have been developed to improve specificity, efficacy, and tolerability.^309^ In addition, targeting upstream UPS components has shown promise; the ubiquitin E1 inhibitor MLN7243 exhibits selective toxicity toward cutaneous squamous cell carcinoma cells while sparing normal keratinocytes, highlighting opportunities for tissue-specific UPS modulation.^264^

#### Therapeutic targeting of the UPR and ERAD in cancer

##### IRE1α inhibitors

UPR signaling and ERAD are critical adaptive pathways that allow cancer cells to survive chronic ER stress. Inhibition of the IRE1α pathway has demonstrated substantial therapeutic potential. STF-083010 selectively inhibits IRE1α RNase activity and induces toxicity in multiple myeloma cells.^272^ MKC-3946 enhances the cytotoxicity of bortezomib and 17-AAG, while Kira8 suppresses tumor growth and potentiates bortezomib and lenalidomide efficacy in vivo.^310,311^ B-I09 synergizes with the BTK inhibitor ibrutinib to induce apoptosis in B-cell malignancies.^312^ In breast cancer, paclitaxel-induced activation of IRE1α promotes pro-tumorigenic cytokine production; combining IRE1α inhibitors with paclitaxel may therefore improve therapeutic outcomes.^313^

##### PERK pathway inhibitors

Targeting the PERK arm of the UPR has also yielded promising results. The PERK inhibitor GSK2656157 induces dose-dependent tumor growth inhibition in pancreatic cancer and multiple myeloma xenografts.^314^ ISRIB restores eIF2B function and enhances gemcitabine-induced pancreatic cancer cell death.^315^ PERK inhibition is particularly effective in drug-resistant breast cancer models.^316^

##### ATF6 inhibitors

Inhibition of ATF6 signaling offers another strategy to overcome chemoresistance. Disruption of the PDIA5-ATF6 axis restores sensitivity in imatinib-resistant leukemia cells, highlighting ATF6 as a therapeutically actionable target.^317^

## Neurodegenerative disorders and protein homeostasis

**Fig. 5:**
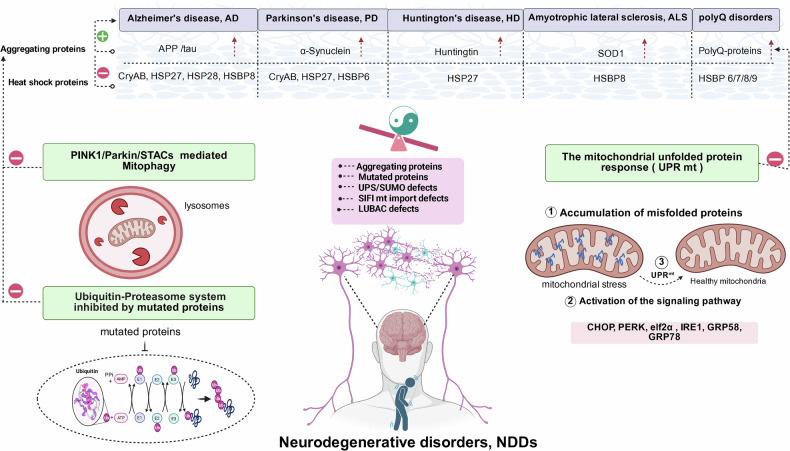
Proteostasis failure and protein aggregation pathways in neurodegenerative disorders (NDDs). Neurodegenerative disorders such as Alzheimer’s disease (AD), Parkinson’s disease (PD), Huntington’s disease (HD), amyotrophic lateral sclerosis (ALS), and polyQ disorders are characterized by the accumulation of aggregating proteins (e.g., APP/tau, α-synuclein, huntingtin, SOD1, polyQ proteins) and dysregulation of heat shock proteins (e.g., CryAB, HSP27, HSPB8). Impairments in protein degradation systems, including the ubiquitin‒proteasome system (UPS) and PINK1/Parkin-mediated mitophagy, contribute to the pathological buildup of misfolded proteins. Additionally, defects in the mitochondrial unfolded protein response (UPRmt) exacerbate mitochondrial stress, further disrupting neuronal proteostasis. Key molecular pathways, including UPS/SUMO modifications, selective mitochondrial import (SIFI), and LUBAC complex dysfunction, collectively contribute to disease pathology. The balance between proteostasis mechanisms and accumulating proteotoxic stress determines neuronal survival in NDDs. This figure was created at Biorender.com

Neurodegenerative disorders (NDDs) are characterized by a progressive loss of neuronal structure and function, leading to cognitive, motor, and behavioral impairments. Among the hallmarks of NDDs, two critical aspects of protein homeostasis stand out: pathological protein aggregation and dysfunctional proteostasis. These processes are central to the pathogenesis of a wide range of NDDs, including Alzheimer’s disease (AD), Parkinson’s disease (PD), primary tauopathies (e.g., progressive supranuclear palsy [PSP], corticobasal degeneration [CBD], tau-linked frontotemporal dementia [FTD-tau]), frontotemporal dementia (FTD), amyotrophic lateral sclerosis (ALS), synucleinopathies (e.g., Lewy body dementia [LBD] and multiple system atrophy [MSA]), Huntington’s disease (HD) and related polyglutamine (polyQ) diseases (e.g., spinocerebellar ataxias [SCA]), and prion diseases (PrD). In most of these disorders, protein aggregates composed of distinct proteins accumulate in specific brain regions, correlating with clinical outcomes and underscoring their pathogenic role (Fig. 5).

### The role of HSPs in NDDs

HSPs play a dual and indispensable role in NDDS: they either facilitate the correct folding and stabilization of misfolded proteins or target irreversibly damaged proteins for degradation via autophagy or UPS. Because neurons are long-lived, post-mitotic cells with limited regenerative capacity, even subtle impairments in protein quality control can lead to cumulative proteotoxic stress and irreversible cellular damage. Among the most extensively studied HSPs in NDDs are HSP70 and HSP90, which are ubiquitously expressed and form the core of the cellular chaperone machinery. In parallel, sHSPs, ranging from approximately 12 to 43 kDa, have emerged as critical modulators of protein aggregation and toxicity in the nervous system (Fig. 5). Together, these chaperone systems constitute a multilayered defense network that buffers neurons against proteotoxic insults and delays disease progression.

#### HSP70 and HSP90 in neurodegenerative proteinopathies

HSP70 and HSP90 interact with a wide spectrum of disease-associated proteins implicated in NDDs, including amyloid-β (Aβ), tau, α-synuclein, and TAR DNA-binding protein 43 (TDP-43). Their ability to dynamically regulate the folding, stabilization, and degradation of these proteins places them at the center of neuronal protein homeostasis. Depending on client protein conformation and cellular context, HSP70 and HSP90 can either promote refolding into nontoxic states or facilitate clearance through proteasomal or autophagic pathways. In AD, HSP70 and HSP90 directly interact with amyloid-β, preventing its aggregation and promoting its clearance. By stabilizing Aβ in a soluble, non-aggregated form, these chaperones limit the formation of toxic oligomers and extracellular plaques, which are hallmark pathological features of AD.^318^ This chaperone-mediated buffering of early aggregation events is particularly important, as soluble Aβ oligomers are now recognized as major drivers of synaptic dysfunction and neurotoxicity.

Tau pathology represents another central target of HSP70 and HSP90 activity. In AD and other tauopathies, these chaperones modulate tau homeostasis by preventing aggregation of hyperphosphorylated tau species and promoting their degradation. Through these mechanisms, HSP70 and HSP90 reduce the formation of neurofibrillary tangles, thereby mitigating one of the core pathological processes underlying tau-mediated neurodegeneration.^319^

In PD, HSP70 and HSP90 interact with α-synuclein, inhibiting its misfolding and aggregation into Lewy bodies. By promoting α-synuclein clearance and preventing the accumulation of toxic oligomeric species, these chaperones reduce α-synuclein-induced neuronal toxicity and cell death.^320^ Similar protective roles are observed in amyotrophic lateral sclerosis (ALS) and frontotemporal dementia (FTD), where HSP70 and HSP90 suppress the aggregation of TDP-43, a protein that forms pathological cytoplasmic inclusions in affected neurons. By stabilizing TDP-43 and facilitating its degradation, these chaperones attenuate its toxic gain-of-function effects.^319,321^

Collectively, the ability of HSP70 and HSP90 to engage multiple aggregation-prone client proteins across distinct NDDs underscores their central role in neuronal proteostasis and highlights their broad therapeutic potential.

#### Small HSPs as first responders to proteotoxic stress

In contrast to HSP70 and HSP90, small heat shock proteins do not actively refold misfolded proteins. Instead, they act as ATP-independent “holdases” that bind misfolded or partially unfolded proteins, preventing their aberrant aggregation and facilitating subsequent degradation. This function is particularly relevant in neurons, where sHSPs frequently colocalize with disease-associated protein aggregates and serve as an early line of defense against proteotoxic stress. αB-Crystallin (CryAB) is among the most extensively studied sHSPs in NDDs. In AD, CryAB is upregulated in the brain and colocalizes with amyloid-β within extracellular senile plaques. Functionally, CryAB counteracts the initial seeding of Aβ oligomers and reduces their neurotoxicity.^322,323^ CryAB also associates with tau fibrils and shows an inverse correlation with granular tau species, suggesting a protective role in limiting tau aggregation and toxicity in tauopathies.^324^

HSP27 is another prominent sHSP implicated in multiple NDDs. In PD, HSP27 protects against α-synuclein-induced toxicity by inhibiting its aggregation and reducing oxidative stress, a major contributor to dopaminergic neuronal loss.^325,326^ In HD, HSP27 exerts neuroprotective effects by suppressing polyglutamine toxicity and lowering ROS levels, thereby slowing neurodegenerative progression.^327^

Additional sHSP family members, including HSBP6, HSBP7, HSBP8, and HSBP9, play important roles in mitigating polyQ-mediated toxicity in HD. Notably, HSBP7 is particularly effective against long polyQ repeats (119Q), whereas HSBP6, HSBP8, and HSBP9 preferentially counteract shorter polyQ expansions (43Q), indicating substrate-length specificity within the sHSP family.^328^ In ALS, HSBP8 protects against aggregation of TDP-43 and its cleavage products (TDP-25 and TDP-35) by promoting their removal via autophagy, further illustrating the tight functional coupling between sHSPs and degradative pathways.^329^

The ability of sHSPs to engage a wide range of aggregation-prone proteins, coupled with their rapid upregulation in response to cellular stress, positions them as critical first responders in neurodegenerative proteostasis. Together with HSP70 and HSP90, sHSPs form an integrated chaperone network that buffers neurons against proteotoxic stress, delays aggregate formation, and promotes clearance of damaged proteins. Understanding how these chaperone systems are coordinated and how their protective capacity declines with age and disease will be essential for developing effective proteostasis-based therapies for NDDs.

### The role of autophagy and mitophagy in NDDs

Autophagy and mitophagy are indispensable for neuronal survival, given the post-mitotic nature of neurons, their extraordinary metabolic demands, and their reliance on precise proteostasis and mitochondrial quality control. Defects in these pathways are now recognized as central drivers of NDDs. Dysregulation of autophagy, endolysosomal trafficking, lysosomal function, and mitochondrial integrity not only promotes neuronal dysfunction and death but also contributes to protein aggregation, neuroinflammation, and disease propagation across neural networks.^330^

#### Autophagy dysfunction and protein aggregation in NDDs

Autophagy genes play critical roles in maintaining neuronal proteostasis by degrading ubiquitinated and aggregated proteins. Disruption of autophagic clearance leads to the accumulation of toxic protein aggregates and neuronal degeneration. The autophagy adaptor p62/SQSTM1, essential for targeting ubiquitinated substrates to autophagosomes, is mutated in ALS and FTD, where impaired p62 function results in aggregation-prone protein accumulation and neuronal toxicity.^331,332^ Multiple PD-associated genes, such as LRRK2, SYNJ1, DNAJC6, and DNAJC13, regulate synaptic endocytosis, autophagy, lysosomal function, and endolysosomal trafficking, underscoring the intertwined regulation of vesicular dynamics and proteostasis in dopaminergic neurons.

PINK1 and Parkin, best known for their role in mitophagy, are also central to maintaining neuronal quality control (Fig. 5). Mutations that impair their function promote mitochondrial damage, oxidative stress, and neuronal death, advancing PD pathology. Excessive α-synuclein aggregation in PD further exacerbates autophagy dysfunction while contributing to neuroinflammation. Misfolded α-synuclein can be released extracellularly, activating microglia and spreading pathogenic seeds or DAMPs to neighboring neurons, amplifying degeneration.^333^ Similarly, aggregation-prone proteins, including mutant huntingtin (Htt) and Ataxin-3, disrupt the autophagy machinery in HD and spinocerebellar ataxias, linking impaired autophagy directly to disease progression.

#### Lysosomal dysfunction and neurodegeneration

Lysosomal dysfunction is a prominent pathological hallmark of NDDs. Loss-of-function mutations in lysosomal proteins, as seen in Niemann-Pick disease type C1 and Gaucher disease, disrupt lipid and cholesterol metabolism and result in profound neurodegeneration. Importantly, several NDD-associated genes, including GBA, GRN, and ATP13A2, are shared with lysosomal storage disorders, reinforcing lysosomal integrity as a critical determinant of neuronal health.^334^ Beyond degradation failure, autophagy-lysosomal dysfunction contributes to the pathological propagation of aggregation-prone proteins. Tau seeds taken up through endolysosomal pathways can trigger lysosomal rupture, releasing aggregates into the cytosol and facilitating their spread.^335^ Similar mechanisms underlie α-synuclein propagation in PD, whereby lysosomal impairment enhances seeding, transmission, and progressive network degeneration.^336^ Thus, autophagy and lysosomal dysfunction not only initiate neuronal stress but also actively drive disease progression and neuroanatomical spread.

#### Mitochondrial dysfunction and mitophagy failure in NDDs

Mitochondrial dysfunction is a defining molecular hallmark across NDDs. The mitochondrial genome is especially susceptible to oxidative damage due to proximity to ROS generated during respiration. The accumulation of mtDNA mutations with aging and disease jeopardizes respiratory capacity, leading to bioenergetic failure, oxidative stress, and neuronal vulnerability.^337,338^ Structural mitochondrial abnormalities such as disrupted cristae are widely documented in FTLD-TDP, AD, mixed AD/PD, and HD, linking altered mitochondrial architecture to impaired ATP production, calcium dysregulation, synaptic dysfunction, and eventual neuronal death.^339–343^ Age-related mitochondrial decline further amplifies these defects, positioning mitochondrial deterioration as both a cause and consequence of neurodegeneration.

Mitophagy serves as the critical defensive mechanism preventing these mitochondrial insults from overwhelming neurons. Impaired mitophagy in AD contributes to the accumulation of dysfunctional mitochondria and exacerbates Aβ and hyperphosphorylated tau pathology. Activation of the PINK1/Parkin pathway restores mitophagy, reduces pathological protein burden, reverses cognitive decline, and improves synaptic function in experimental AD models.^344^ Key regulatory proteins such as VDAC1 and DRP1 influence this process; targeted reduction of these proteins enhances mitophagy and improves neuronal outcomes.^345^ In PD, defective PINK1/Parkin-dependent mitophagy leads to the accumulation of damaged mitochondria, neuronal stress, and dopaminergic neurodegeneration. Mutations in GBA and LRRK2 further impair mitophagy signaling, compounding mitochondrial dysfunction and accelerating disease progression.^346,347^ In HD, mutant huntingtin interferes with lysosomal degradation of damaged mitochondria, disrupting GAPDH-mediated mitophagy and resulting in mitochondrial accumulation and neuronal death; enhancing mitophagy restores mitochondrial function and attenuates neurodegeneration.^348^

### The role of the UPS in NDDs

The ubiquitin‒proteasome system (UPS) represents a highly coordinated protein quality-control network responsible for selectively tagging, recognizing, and degrading damaged, misfolded, or excess proteins through ubiquitination. Given the brain’s unique susceptibility to proteotoxic stress, owing to its post-mitotic neuronal population, high metabolic demand, and reliance on precise proteome regulation, UPS integrity is indispensable for neuronal survival. Disruption of UPS function is a central pathogenic feature across a broad spectrum of NDDs.

#### Pathogenic mutations in UPS components as direct drivers of neurodegeneration

The indispensable role of the UPS in neuronal health is most clearly illustrated by familial forms of NDDs caused directly by mutations in UPS constituents. These genetic disorders demonstrate that intact ubiquitin-dependent protein degradation is not merely supportive but fundamentally required for neuronal integrity.

Mutations in UBQLN2, which encodes a ubiquitin-like protein involved in shuttling ubiquitinated substrates to the proteasome, are linked to ALS and FTD. UBQLN2 dysfunction compromises substrate delivery to the proteasome, resulting in the accumulation of toxic protein aggregates and progressive neurodegeneration.^349^ Similarly, mutations in valosin-containing protein (VCP), a multifunctional AAA-ATPase essential for extracting ubiquitinated proteins from cellular structures and directing them toward degradation, cause multisystem proteinopathies that include ALS, FTD, and inclusion body myopathy. VCP dysfunction disrupts ubiquitin-dependent protein turnover, leading to widespread proteostasis failure and aggregate accumulation.^350,351^

Loss-of-function mutations in Parkin (PARK2), an E3 ubiquitin ligase, underlie autosomal recessive Parkinson’s disease. Parkin plays a dual role in ubiquitinating misfolded proteins and targeting damaged mitochondria for degradation. Its inactivation impairs MQC, promotes the accumulation of dysfunctional mitochondria, and accelerates dopaminergic neuronal loss.^352^

Mutations in UCHL1, which encodes a deubiquitinating enzyme critical for ubiquitin recycling and proteasome efficiency, are associated with rare progressive neurodegenerative syndromes. UCHL1 dysfunction reduces the availability of free ubiquitin and compromises proteasomal activity, further destabilizing neuronal proteostasis.^353^

Collectively, these genetic disorders underscore the non-redundant role of the UPS in preventing protein aggregation and preserving neuronal viability. Importantly, they also illustrate how defects at distinct levels of the UPS, substrate recognition, ubiquitin recycling or proteasome targeting, can converge on a common outcome of proteotoxic stress and neurodegeneration.

#### Protein aggregation-driven UPS impairment in sporadic NDDs

In addition to genetic mutations, UPS dysfunction is also a defining feature of sporadic NDDs, where aggregation-prone proteins directly impair proteasomal function. Proteins such as tau, TDP-43, α-synuclein, and polyglutamine-expanded proteins misfold and aggregate into toxic species that overwhelm the UPS and interfere with normal degradation processes.

In AD and related tauopathies, hyperphosphorylated tau assembles into neurofibrillary tangles that impair proteasome activity and disrupt cellular homeostasis.^354^ These aggregates not only evade degradation but also obstruct the processing of other UPS substrates. In ALS and FTD, cytoplasmic aggregates of TDP-43 sequester UPS components, thereby preventing the clearance of additional misfolded proteins.^355^ Moreover, TDP-43 aggregates can translocate into mitochondria, triggering mitochondrial DNA release into the cytoplasm and activating inflammatory signaling through the cGAS-STING pathway.^333,356^ This links UPS dysfunction to innate immune activation and neuroinflammation.

In PD and other synucleinopathies, aggregated α-synuclein impairs proteasomal activity and disrupts mitochondrial function, further exacerbating neuronal stress.^357^ Similarly, in HD and related polyQ disorders, expanded polyQ-containing proteins form insoluble aggregates that physically inhibit proteasome function, amplifying the proteotoxic burden.^358^

Together, these findings reveal a feedforward loop in which misfolded proteins evade degradation while simultaneously crippling the UPS, accelerating neuronal dysfunction and degeneration. This mechanism provides a unifying explanation for UPS impairment in both familial and sporadic NDDs.

#### Non-canonical ubiquitination and SUMOylation in NDDs

In addition to canonical K48-linked ubiquitination that targets proteins for proteasomal degradation, alternative ubiquitin chain architectures and ubiquitin-like modifications play critical roles in neuronal signaling and survival (Fig. 2c). Dysregulation of these non-canonical pathways contributes substantially to NDD pathogenesis.

K63-linked ubiquitination, which typically regulates signaling, DNA repair, and autophagy rather than degradation, is frequently altered in PD, AD, and ALS. Aberrant K63-linked ubiquitination disrupts intracellular signaling networks and autophagic flux, contributing to the accumulation of toxic protein species and impaired stress responses.^359^

Linear ubiquitination, mediated by the linear ubiquitin chain assembly complex (LUBAC), regulates NF-κB signaling and inflammatory pathways. Dysregulation of LUBAC activity has been linked to NDDs, where excessive or sustained inflammatory signaling exacerbates neuronal injury and cell death.^360^

SUMOylation, a ubiquitin-like modification, further expands the regulatory complexity of neuronal proteostasis. SUMOylation modulates protein stability, subcellular localization, transcriptional activity, synaptic plasticity, and neuronal excitability. Disruption of SUMOylation has been implicated in AD, HD and PD, where it contributes to protein aggregation, synaptic dysfunction, and neuronal vulnerability.^361^

These non-canonical ubiquitin-dependent modifications emphasize that UPS dysfunction in NDDs extends beyond proteasomal degradation alone. Instead, it reflects a broader collapse of ubiquitin-centered regulatory networks that integrate proteostasis, signaling, inflammation, and neuronal function.

### The role of ER stress and UPR in NDDs

The UPR is a critical cellular mechanism activated in response to the accumulation of misfolded or unfolded proteins in the ER. As neurons are highly active and require substantial protein synthesis and trafficking, they are particularly susceptible to ER stress. However, in NDDs, chronic or overwhelming ER stress shifts the UPR toward maladaptive responses, contributing to neuronal dysfunction and death, e.g., Prolonged PERK activation has been implicated in AD, PD, and ALS, where it contributes to chronic ER stress and neuronal death. Similarly, IRE1 dysregulation has been observed in tauopathies, PrD, and HD, highlighting its role in neurotoxicity, whereas ATF6 dysfunction exacerbates protein aggregation and has been linked to FTD and tauopathies.

UPR activation varies across different brain regions and disease contexts, reflecting the selective vulnerability of neuronal populations. For example, PERK and phosphorylated eIF2\u03b1 are prominently activated in the substantia nigra of PD patients, correlating with dopaminergic neuronal loss.^362^ In AD, elevated ATF6 and XBP1 levels are observed in the hippocampus, where tau tangles and amyloid-\u03b2 plaques accumulate.^363,364^ Similarly, GRP78 upregulation is detected in ALS spinal motor neurons, and region-specific UPR markers such as GRP58 and CHOP are found in prion diseases and HD, respectively.^365–369^ This region-specific activation suggests that UPR-mediated responses may vary depending on the type and extent of proteostasis disruption in different NDDs. However, in all cases, sustained UPR activation promotes neurodegeneration by exacerbating ER stress, initiating apoptotic pathways, and impairing cellular recovery mechanisms.

### Therapeutic targeting of proteostasis in NDDs

NDDs are unified by the progressive accumulation of misfolded and aggregation-prone proteins, chronic cellular stress, and selective neuronal vulnerability. Given the limited regenerative capacity of neurons, sustained disruption of proteostasis progressively overwhelms cellular quality control systems, leading to synaptic dysfunction, neurodegeneration and irreversible cognitive or motor decline. Consequently, therapeutic strategies aimed at restoring proteostasis have emerged as central disease-modifying approaches.

#### Targeting HSPs in neurodegenerative diseases

##### HSP70 modulators

HSP70 modulators have received considerable attention. Geranylgeranylacetone (GGA), a non-toxic HSP70 inducer, reduces α-synuclein aggregation in PD models and attenuates tau pathology in AD models.^370^ BGP-15 enhances HSP70 expression and reduces mutant huntingtin aggregation in HD models,^371^ while YM-1 increases HSP70 activity through modulation of its co-chaperone Hsp40, leading to reduced tau aggregation in AD models.

##### HSP90 inhibitors

HSP90 inhibition represents another extensively explored strategy. Ansamycin derivatives such as 17-AAG (tanespimycin) and 17-DMAG (alvespimycin) reduce tau and α-synuclein oligomerization in AD and PD models by promoting their degradation via the ubiquitin‒proteasome system.^372^ Although 17-AAG has demonstrated safety in oncology, its limited blood‒brain barrier permeability constrains its utility in NDDs, while 17-DMAG exhibits improved solubility but unacceptable toxicity.^373^ Next-generation HSP90 inhibitors aim to overcome these limitations. SNX-0723, identified through scaffold-based screening, selectively binds the ATP-binding pocket of HSP90 and demonstrates improved brain penetration, reducing mutant huntingtin aggregation in PD and HD models.^374^ PU-H71 similarly reduces tau pathology and improves cognitive function in AD models.^375^

##### HSP27 modulators

HSP27 modulation has also shown therapeutic promise. Celastrol, a natural compound, enhances HSP27 expression and reduces α-synuclein and tau aggregation in PD and AD models.^376^ Arimoclomol, a co-inducer of HSP27 and HSP70, has demonstrated efficacy in ALS and HD models by reducing protein aggregation and improving neuronal survival.^377^

##### Heat shock factor 1 (HSF1) activators

Upstream activation of HSF1 provides an additional strategy to coordinately induce multiple HSPs. HSF1A enhances HSP70 and HSP40 expression, reducing tau and α-synuclein aggregation in AD and PD models.^378^ Riluzole, an FDA-approved drug for ALS, indirectly activates HSF1 and shows potential in reducing protein aggregation in ALS and HD models.^379^

#### Therapeutic modulation of the autophagy-mitophagy axis in NDDs

##### Pharmacological agents

Multiple pharmacological agents enhance autophagy through distinct regulatory nodes. mTOR inhibition with rapamycin (sirolimus) robustly induces autophagy, reducing tau and amyloid-β aggregation in AD models as well as mutant huntingtin in HD models.^380^ Everolimus, a rapamycin analog with improved pharmacokinetics, similarly reduces α-synuclein aggregation in PD models.^381^ AMPK activation represents an alternative autophagy-inducing strategy. Metformin enhances autophagy and reduces tau and amyloid-β pathology in AD models,^382^ while AICAR reduces α-synuclein aggregation and improves motor function in PD models.^383^

##### TFEB and Beclin-1 activators

TFEB, a master regulator of lysosomal biogenesis and autophagy, has emerged as a particularly attractive target. Trehalose activates TFEB and enhances autophagic clearance of tau and α-synuclein in AD and PD models.^384^ A synthetic curcumin analog (C1) similarly activates TFEB and reduces amyloid-β and tau pathology in AD models.^385^ Direct enhancement of autophagosome formation via Beclin-1 has also shown promise; the Tat-Beclin-1 peptide reduces α-synuclein and mutant huntingtin aggregation in PD and HD models.^386,387^

##### Autophagy-inducing small molecules and natural compounds

Additional autophagy-inducing agents include lithium, which induces autophagy via inhibition of inositol monophosphatase and reduces tau and amyloid-β aggregation in AD models,^388^ and SMER28, a small-molecule enhancer of rapamycin that promotes autophagy and reduces tau and α-synuclein aggregation in AD and PD models.^389^ Natural compounds such as resveratrol, which induces autophagy through SIRT1 activation,^390^ and spermidine, a polyamine that enhances autophagic flux, further demonstrate the therapeutic potential of autophagy induction in NDDs.^157,391^

##### Mitophagy-targeted strategies

Mitophagy-targeted strategies are increasingly recognized as essential complements to bulk autophagy. Enhancing mitochondrial biogenesis with bezafibrate activates PGC-1α, improves mitochondrial function, and reduces motor deficits in HD models.^392^ Mitochondrial antioxidants such as MitoQ and coenzyme Q10 reduce oxidative damage and improve mitochondrial function in PD, AD, and HD models.^393^ Direct mitophagy enhancers include urolithin A, which induces mitophagy and improves mitochondrial function in AD and PD models,^394^ and kinetin and its analog MTK458, which stabilize PINK1 and enhance Parkin-mediated mitophagy in PD models.^395,396^

##### Mitochondrial dynamics modulators

Modulation of mitochondrial dynamics further intersects with mitophagy regulation. Mdivi-1 inhibits excessive mitochondrial fission by targeting Drp1, improving mitochondrial function in AD and HD models.^397^ BGP-15 stabilizes OPA1, promoting mitochondrial fusion and reducing oxidative stress in HD models.^398,399^ Mild mitochondrial uncoupling with 2,4-dinitrophenol (DNP) reduces oxidative stress and improves mitochondrial function in PD and HD models.^400,401^

#### UPS-targeted therapies in NDDs

##### Proteasome activation

The ubiquitin‒proteasome system plays a central role in clearing misfolded and damaged proteins, and its dysfunction contributes directly to protein aggregation in NDDs. Proteasome activation has therefore emerged as a therapeutic strategy. Rolipram, a phosphodiesterase-4 inhibitor, indirectly enhances proteasome activity by increasing cAMP levels and reduces tau aggregation in AD models.^402^

##### Targeting UPS regulators

Targeting UPS regulators offers additional specificity. N-aryl benzimidazole (NAB) compounds enhance Parkin activity, promote clearance of damaged mitochondria and reduce oxidative stress in PD.^403^ Deubiquitinating enzyme inhibitors such as IU1 and its derivative IU1-248 inhibit USP14, enhancing proteasome activity and reducing tau aggregation in AD and α-synuclein aggregates in PD.^404^ FT385 inhibits USP30, promoting mitophagy and reducing mitochondrial dysfunction in PD models.^404^

##### Aggresome pathway modulators

Modulation of aggresome-autophagy coupling has also shown promise. HDAC6 inhibitors such as tubastatin A and the clinically approved ACY-1215 (rocilinostat) enhance aggresome formation and autophagic clearance, reducing tau and α-synuclein aggregates in AD and PD models.^405^

##### Proteasome inhibitors

Although proteasome inhibition is generally associated with toxicity, low-dose inhibition has been explored as a strategy to induce compensatory autophagy. For example, low-dose bortezomib induces autophagy and reduces mutant huntingtin aggregation in HD models.^406^ UPS-targeting peptides and engineered ubiquitin variants further expand therapeutic possibilities by selectively modulating E3 ligases or DUBs.^407^ Notably, TRIM11 promotes proteasomal degradation of tau while simultaneously acting as a molecular chaperone that suppresses tau misfolding.^408,409^

##### PROTACs

Targeted protein degradation technologies are also emerging in NDDs. PROTAC-based approaches have been developed to selectively degrade aggregation-prone proteins, including QC-01-C175 for tau in AD, XL01126 for LRRK2 in PD, and GW5074 for mutant huntingtin in HD.^172,410,411^ These strategies, although offering unprecedented specificity, remain limited by blood‒brain barrier delivery challenges.

Despite substantial progress, UPS-based therapies face key obstacles, including achieving substrate specificity and efficient CNS delivery. Future advances will depend on innovative drug delivery systems and integration with complementary proteostasis-modulating strategies.

#### Therapeutic targeting of ER stress and ERAD in NDDs

##### ERAD enhancers

Enhancing ER-associated degradation (ERAD) represents one therapeutic avenue. Eeyarestatin I stabilizes the p97/VCP complex, enhancing ERAD activity and reducing misfolded protein accumulation in HD and ALS models.^412^ AA147, an activator of ATF6, enhances ERAD capacity and reduces tau and amyloid-β aggregation in AD models.^413^

##### IRE1α and PERK modulators

Targeting specific UPR signaling branches has also shown promise. IRE1α inhibition with KIRA6 or 4μ8C reduces ER stress, neuronal death, and tau aggregation in PD, ALS, and AD models.^414,415^ Modulation of the PERK pathway offers additional benefits. The PERK inhibitor GSK2606414 reduces neurodegeneration in prion disease and tauopathy models,^416^ while ISRIB restores protein synthesis and attenuates neurodegeneration in AD models.^417^

##### Chemical chaperones

Chemical chaperones provide a complementary strategy to alleviate ER stress. TUDCA reduces ER stress and neurodegeneration in HD, PD, and ALS models,^418^ while 4-PBA improves motor function and reduces ER stress in AD, PD, and HD models.^419,420^

Although UPR modulation holds substantial promise, successful translation will require a nuanced understanding of context-specific UPR signaling across disease stages. Biomarkers reflecting UPR activation status may aid in patient stratification and therapeutic monitoring. Integrating UPR-targeted therapies with autophagy-mitophagy or chaperone-based strategies may yield synergistic neuroprotective effects.

## Autoimmune diseases and protein homeostasis

Autoimmune diseases emerge from a catastrophic failure of immune tolerance, where the body’s defense system misidentifies its own cells as threats, leading to relentless inflammation and self-destruction. At the heart of this dysregulation lies proteostasis: cellular stress-response pathways, including HSPs, autophagy, mitophagy, the UPS, and the UPR, work in harmony to maintain immune equilibrium (Fig. 1a). However, when these systems falter, the consequences are profound: misfolded proteins accumulate, aberrant antigen presentation fuels immune hyperactivation, and chronic inflammation spirals out of control. From systemic lupus erythematosus (SLE) and rheumatoid arthritis (RA) to MS and autoimmune skin disorders, the disruption of proteostasis not only amplifies disease pathology but also renders conventional therapies less effective.

### Heat shock proteins in autoimmune diseases

The role of HSPs in autoimmune diseases is particularly evident in SLE, where altered HSP expression and the production of anti-HSP autoantibodies contribute significantly to disease pathology.

#### Disease-specific immunopathological roles of HSPs in autoimmune disorders

**Fig. 6:**
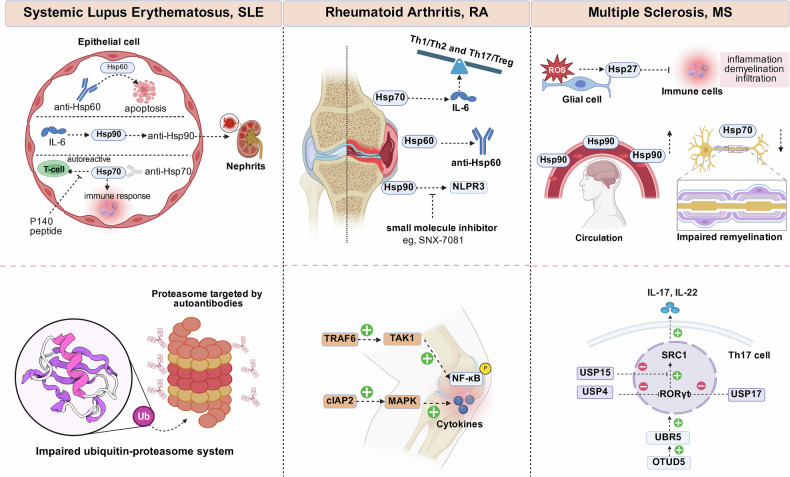
Proteostasis dysregulation as a driver of autoimmune pathology. This figure highlights the crucial role of protein homeostasis disruptions in autoimmune diseases, emphasizing heat shock proteins, proteasome impairment, and ubiquitination pathways as potential therapeutic targets. (Left) Systemic lupus erythematosus (SLE): The interplay between heat shock proteins (Hsp60, Hsp70, and Hsp90) and autoantibodies promotes worsening autoimmunity. Autoantibodies against Hsp60 and Hsp90 contribute to immune activation, IL-6 production, and loss of immune tolerance. The P140 peptide plays a role in modulating T-cell autoreactivity. Additionally, impaired ubiquitin‒proteasome function due to autoantibody targeting further exacerbates disease pathology. (Center) Rheumatoid Arthritis (RA): Hsp70 influences T-cell polarization by affecting the Th1/Th2 and Th17/Treg balance, while Hsp60 induces autoantibodies that contribute to disease progression. Hsp90 facilitates NLPR3 inflammasome activation, which can be inhibited by small molecule inhibitors such as SNX-7081. Inflammatory signaling cascades involving TRAF6, TAK1, and MAPK lead to NF-κB activation and cytokine release, driving chronic joint inflammation. (Right) Multiple sclerosis (MS): Oxidative stress (ROS) induces Hsp27 expression in glial cells, contributing to immune activation, inflammation, demyelination, and infiltration of immune cells. Circulating Hsp90 and neuronal Hsp70 are linked to impaired remyelination. The Th17 pathway, central to MS pathogenesis, is regulated by the deubiquitinases USP15, USP4, and USP17, which modulate the transcription factor RORγt, driving IL-17 and IL-22 production. This figure was created at Biorender.com

Patients with SLE exhibit elevated levels of IgM anti-65 kDa HSP antibodies compared to healthy controls,^421^ and a significant subset overexpress HSP90.^422^ HSP60, which is present on the surface of endothelial cells (ECs), serves as a target for anti-HSP60 antibodies in SLE sera. These antibodies bind to ECs and induce apoptosis, contributing to tissue damage (Fig. 6).^423^ In RA, HSPs are implicated in driving inflammation and shaping the autoimmune environment through their immunogenic properties. Patients with RA exhibit increased levels of IgM anti-65 kDa and IgG anti-70 kDa HSP antibodies compared to healthy controls.^421^ Synovial tissues from RA patients show significant upregulation of HSP27 mRNA,^424^ and elevated levels of pro-inflammatory IL-6 are associated with increased IgG autoantibodies against HSP40, making HSP40 a potential marker of disease activity and progression.^425,426^ Additionally, IgG, IgM, and IgA autoantibodies against HSP60, HSP70, and HSP90 are significantly elevated in RA patients.^427^

In MS, HSPs contribute to the regulation of neuroinflammation and are linked to autoantigenicity and immune dysregulation in the central nervous system (CNS).^428,429^ HSPs are induced in response to various CNS pathologies, including neurodegenerative diseases, ischemia, and epilepsy, where they play a neuroprotective role by preventing protein aggregation and misfolding.^430,431^ However, in MS, HSPs can exacerbate the immune response by acting as pro-inflammatory cytokines and adjuvants for myelin peptides.^432^ Patients with MS exhibit elevated levels of IgG antibodies targeting HSP70 family members, although autoantibodies against HSP27, HSP60, and HSP90 are not typically detected.^429,433^ HSP27 is strongly implicated in MS pathogenesis as the most immunodominant myelin T-cell antigen, with up to 20-fold increased expression in MS lesions (Fig. 6).^434^ While HSP27 is induced in glial cells by oxidative stress and acts as an intracellular signaling factor, its upregulation is generally protective. HSP27-deficient mice develop more severe symptoms, including increased inflammation, demyelination, and immune cell infiltration, which are ameliorated by exogenous HSP27 treatment.^435^

#### HSPs and inflammasome regulation in autoimmune diseases

Inflammasomes are multiprotein complexes that play a central role in innate immunity by regulating the production of pro-inflammatory cytokines, such as interleukin-1β (IL-1β) and IL-18. HSPs are essential for the proper folding, stabilization, and assembly of key inflammasome components, including NLRP3, ASC, and caspase-1. By maintaining protein homeostasis under stress conditions, HSPs directly influence the activation and function of inflammasomes. Dysregulation of HSP activity can lead to aberrant inflammasome activation, resulting in the excessive production of pro-inflammatory cytokines and contributing to the pathogenesis of autoimmune diseases such as SLE, RA, and MS.^436–438^

#### Anti-HSP autoantibodies in autoimmune diseases

A key feature of autoimmune diseases is the presence of autoantibodies that target self-proteins, including HSPs. Anti-HSP autoantibodies are elevated in patients with a wide range of inflammatory and autoimmune conditions, including RA,^427^ juvenile idiopathic arthritis,^439^ psoriasis,^440^ SLE,^441^ atopic dermatitis,^442^ and celiac disease.^443^ These autoantibodies can amplify inflammatory processes, disrupt normal protein homeostasis, and exacerbate tissue damage, playing a critical role in disease pathogenesis. Interestingly, anti-HSP autoantibodies are also found in healthy individuals, likely as a result of prior bacterial infections or tissue degradation.^444^ Mutations in HSP genes can further contribute to immune dysregulation. For example, a variant of the HSPA1L gene has been linked to susceptibility to vitiligo, an autoimmune condition characterized by the destruction of melanocytes.^445^ Additionally, intracellular HSP90, which is involved in the activation of both innate and adaptive immune responses, has been implicated in autoimmune processes. HSP90 inhibitors have shown promise in preclinical models of autoimmune diseases such as arthritis and SLE by suppressing inflammatory signaling pathways.^446^ These inhibitors, such as geldanamycin, bind to the N-terminal nucleotide-binding pocket of HSP90 with higher affinity than ATP, leading to the proteasomal degradation of HSP90-dependent client proteins. This results in the inactivation of NF-κB-dependent inflammatory factors such as TNF-α and IL-6, as well as the blockade of cell signaling molecules such as MAP kinases.^446^ In contrast, upregulation of HSP70 inhibits NF-κB activity, reducing inflammation and highlighting the complex roles of HSPs in immune regulation.

### Autophagy and mitophagy in autoimmune diseases

Autophagy and mitophagy together form a critical quality-control network that regulates immune cell survival, antigen presentation, inflammasome activity, and tissue homeostasis. Dysregulation of these pathways contributes profoundly to autoimmune pathogenesis by impairing the clearance of damaged organelles and intracellular debris, promoting chronic inflammation, and sustaining autoreactive immune responses. Failure to eliminate dysfunctional mitochondria leads to the accumulation of mitochondrial ROS and the release of immunogenic mtDNA, both of which act as DAMPs that activate innate immune pathways, including the NLRP3 inflammasome and type I interferon (IFN-I) signaling. Thus, disrupted autophagy and mitophagy drive a persistent inflammatory milieu that underlies autoimmune diseases.

#### Autophagy as a regulator of immune tolerance and inflammation

Autophagy plays a dual role in autoimmunity by regulating immune cell function, antigen processing, and inflammatory responses. Defective autophagic degradation promotes accumulation of intracellular autoantigens and enhances presentation of self-derived peptides, contributing to loss of self-tolerance. Autophagy also normally constrains inflammasome activation; failure of this regulation allows excessive IL-1β and IL-18 production, amplifying systemic inflammation, which is particularly relevant in SLE and RA.

In SLE, dysregulated autophagy contributes to immune hyperactivation, defective apoptotic cell clearance, and increased autoantigen availability. Genetic studies strongly support the involvement of autophagy machinery in SLE susceptibility, with GWAS identifying risk polymorphisms in ATG5, ATG7, and MTMR3.^447–450^ Upregulated autophagy in macrophages enhances the production of pro-inflammatory cytokines such as IL-6 and TNF-α, while autophagy deficiency promotes exaggerated inflammasome activation, as demonstrated by ATG16L1 ablation increasing IL-1β and IL-18 secretion.^451,452^ Together, these abnormalities destabilize immune tolerance and promote lupus nephritis, immune complex deposition, and tissue injury.

In RA, autophagy supports many of the pathological hallmarks of disease progression. Upregulation of autophagy-related proteins (including BCL-1, ATG7, ATG5, and LC3-II) in synovial tissues contributes to synovial fibroblast proliferation, resistance to apoptosis, and chronic inflammatory activation.^453–458^ Autophagy also enhances osteoclastogenesis through an overactive autophagy-lysosomal system, facilitating cartilage erosion and bone destruction.^455^ Experimental inhibition of autophagy alleviates synovial inflammation and promotes apoptosis through PI3K/AKT modulation, underscoring autophagy’s pathogenic role in RA.

In MS, autophagy plays a uniquely context-dependent role. It can promote neuroprotection by limiting inflammation and oxidative stress, yet excessive or dysregulated autophagy also enhances autoreactive immune responses and demyelination. Differential expression of autophagy genes in MS patients, downregulation of ATG9A and BECN1 and upregulation of ULK1, ULK2, and ATG5 reflect complex autophagy modulation.^459,460^ Reduced LC3 and BCL-1 in spinal cord tissues, along with evidence that mTORC1 inhibition ameliorates disease, supports a pathogenic dimension of autophagy in MS.^461^ Conversely, interventions such as caloric restriction enhance autophagy, improve remyelination, and reduce disease severity in relapsing-remitting MS, emphasizing its therapeutic potential when properly modulated.^462^

#### Mitochondrial dysfunction, mtDNA instability, and impaired mitophagy

Mitochondria are essential regulators of immune signaling, apoptosis, metabolism, and stress responses. In autoimmune diseases, mitochondrial dysfunction and impaired mitophagy fuel chronic inflammation by releasing ROS and mtDNA, which activate innate immune pathways and promote autoreactive immune responses. Damaged mitochondria that escape degradation act as persistent inflammatory stimuli, linking mitochondrial instability directly to autoimmune disease mechanisms. In SLE, mitochondrial dysfunction contributes to oxidative stress, altered apoptosis, mtDNA mutations, and impaired immune complex clearance, aggravating the loss of self-tolerance.^30^ SLE T cells display increased mitochondrial mass, reflecting enhanced biogenesis but insufficient mitophagy, alongside elevated IFN-α signaling that further suppresses mitophagic clearance and primes dendritic cell activation.^463,464^ mtDNA damage and immunogenic modifications promote autoantibody production and lupus nephritis, while genetic associations such as mtDNA haplotype N and D-loop heterogeneity support a mitochondrial genetic risk component.^465–468^

In RA, mitochondrial dysfunction drives chronic synovial inflammation despite preserved mitochondrial mass. RA T cells exhibit reduced respiratory capacity and abnormal mitochondrial dynamics influenced by microRNAs such as miR-125b, which regulates the mitochondrial fission protein MTP18.^469–471^ Hypoxic synovial environments enhance mitochondrial activity in osteoclasts, exacerbating bone erosion.^472^ Elevated extracellular mtDNA in synovial fluid and plasma correlates with autoantibody presence and inflammation, while mtDNA mutations in synovial tissues are associated with increased TNF-α and IFNγ signaling, establishing a self-reinforcing inflammatory cycle.^473–475^

In MS, mitochondrial injury contributes to both neuroinflammation and neurodegeneration. The CNS’s high metabolic demand renders it particularly vulnerable to oxidative injury.^476^ Oligodendrocyte differentiation and myelination are impaired by mtDNA damage and oxidative stress, leading to altered transcription, double-strand breaks, and mitochondrial deletion events, especially involving complex IV.^477,478^ Reduced N-acetyl aspartate levels reflect compromised mitochondrial integrity and correlate with poor myelin maintenance.^479^ Mitochondrial dysfunction disturbs T-cell homeostasis by activating cell death pathways involving Bcl2, OPA1, PHB2, ATG5, OMA1, and SIRT3, while reduced OXPHOS, membrane potential instability, and altered glycolysis further destabilize immune function.^480–483^

### The role of the UPS in autoimmune diseases

The UPS regulates processes relevant to autoimmune diseases, such as antigen presentation, cytokine production, T-cell receptor signaling, and inflammasome activation, making it a central player in immune homeostasis and inflammation.^484,485^

#### UPS dysregulation in SLE: autoantibodies, antigen processing, and immune signaling

In SLE, aberrant UPS activity contributes to inflammatory pathways, defective antigen presentation, and impaired apoptotic processes. SLE patients exhibit autoantibodies against the α and β subunits of the 20S proteasome and other UPS components, which can block proteasome activation by PA28 in vitro, suggesting a regulatory role for these autoantibodies in proteasome function (Fig. 6).^486^ Elevated levels of circulating proteasomes correlate with SLE disease activity, making them potential biomarkers for lupus.^487^ Neutrophil extracellular traps (NETs), which are implicated in SLE pathogenesis, contain lower levels of polyubiquitinated proteins in SLE patients. Anti-ubiquitinated myeloperoxidase antibodies in SLE sera enhance cytokine production by macrophages upon NET stimulation, suggesting a role for extracellular ubiquitin in lupus-related inflammation.^488^

Polymorphisms in TNFAIP3 (A20), a DUB that removes K63-linked ubiquitin chains and adds K48-linked chains to signaling intermediates, are associated with SLE. Targeting TNFAIP3 and UBE2L3 represents a potential therapeutic strategy for SLE.^489^ E3 ubiquitin ligases, such as STUB1 and FBXW7, are also implicated in SLE. STUB1 is upregulated in CD4+ T cells of SLE patients and promotes the expression of CD70 and CD11a, making it a potential therapeutic target.^490^ FBXW7, which catalyzes the degradation of the anti-apoptotic protein MCL1, plays a key role in myeloid cells and SLE progression. FBXW7 deficiency in mice reduces immune complex deposition and glomerulonephritis, highlighting its importance in SLE pathogenesis.^491^

#### UPS-controlled inflammatory signaling and synovial pathology in RA

In RA, the UPS is involved in multiple interconnected pathways that drive chronic inflammation and joint damage. Polyubiquitination of signaling molecules such as TRAF2, TRAF6, RIP-1, and NEMO activates the TAK1 and IκB kinase (IKK) complexes, leading to NF-κB activation and pro-inflammatory cytokine production.^492^ The E3 ligases TRAF2 and TRAF6 play a central role in this process, linking the UPS to RA pathogenesis. Oxidative stress in RA overloads the UPS, leading to the accumulation of ubiquitin conjugates and exacerbating cellular damage and inflammation. Proteasome inhibitors have shown promise in RA by reducing NF-κB activation, IL-6 expression, and angiogenesis, thereby alleviating arthritis symptoms.^493^

The E3 ligase cIAP2 promotes FLS survival and inflammatory responses in RA (Fig. 6), whose inhibition reduces cytokine secretion and FLS proliferation, making it a potential therapeutic target.^494^ Similarly, inhibition of the E3 ligase synoviolin suppresses synovial cell proliferation and reduces disease severity in RA models. Polymorphisms in TNFAIP3 and elevated levels of circulating proteasomes are also associated with RA, further implicating the UPS in disease pathogenesis.^487,495^ Inhibitors targeting TRAF6-Ubc13 interactions, such as C25-140, ameliorate inflammation and improve symptoms in RA models by blocking NF-κB activation (Fig. 6).^496^ Meta-analyses have identified TRAF6 and RAG1 as true RA risk alleles, underscoring the importance of UPS components in RA.^497^

#### UPS regulation of T-cell differentiation and neuroinflammation in MS

In MS, the UPS regulates T-cell responses and CNS inflammation. T-cell receptor (TCR) signaling and Th17 cell differentiation, which are critical in MS, are influenced by ubiquitination and deubiquitination processes.^498^ DUBs such as USP9X, USP15, and CYLD regulate TCR signaling, while USP4 and USP17 stabilize the transcription factor RORγt, a key driver of Th17 differentiation (Fig. 6).^499–505^ The DUB OTUD5 (DUBA) promotes RORγt degradation by stabilizing the E3 ligase UBR5, highlighting the intricate regulation of Th17 cells by the UPS.^506^ Polymorphisms in CBL and CBL-B are associated with MS, with reduced CBL-B expression enhancing CD4+ T-cell proliferation in response to type I interferons.^507^ Loss-of-function mutations in CBL are also linked to autoreactive inflammatory disorders, further implicating the UPS in MS pathogenesis.^508^ The UPS regulates myeloid leukemia 1 (MCL-1), an anti-apoptotic protein upregulated in MS. Dysregulation of the UPS and interferon-beta (IFN-β) signaling contributes to MS development, making UPS components potential therapeutic targets.

### ER stress in autoimmune diseases

ER stress influences immune cell survival, activation, differentiation, and effector functions, and its dysregulation disrupts immune homeostasis, contributing to the development and progression of autoimmune diseases.

#### ER stress-driven B-cell differentiation and autoantibody production in SLE

Autoantibodies associated with SLE can exacerbate ER stress and activate the ER stress response. For instance, antibodies targeting the ER stress-related protein GRP78 have been detected in the blood of SLE patients, with increased GRP78 and XBP1 expression observed in antibody-secreting plasma cells. This suggests that ER stress drives B-cell differentiation into plasma cells, enhancing antibody production. Activation of the IRE1-XBP1 pathway is essential for ER expansion and antibody secretion in plasma cells, further implicating ER stress in SLE pathogenesis.^509^ Clinical evidence indicates that the expression of ER stress-related proteins such as IRE1, PERK, and CHOP is reduced in SLE patients, while the levels of XBP1 and midbrain astrocyte-derived neurotrophic factor (MANF) are elevated, suggesting their potential role in disease development.^510^

#### Hyperactive ERAD and IRE1 signaling in synovial inflammation and cell survival in RA

In RA, hyperactivation of the ERAD system, known as hyper-ERAD, contributes to synoviocyte proliferation and mitigates severe ER stress by promoting an anti-apoptotic response. Synoviolin, an ER-resident E3 ubiquitin ligase involved in the ERAD pathway, is highly expressed in the synovium of RA patients and plays a key role in disease progression.^511^ It facilitates the ubiquitination and degradation of IRE1α in FLS from mice with collagen-induced arthritis (CIA), protecting synovial cells from ER stress-induced apoptosis. Elevated levels of ER stress markers, including GRP78, IRE1, XBP1s, ATF6, and eIF2α-P, have been observed in macrophages and synovial tissues of RA patients.^458^

Inflammatory cytokines and autoantibodies implicated in RA pathogenesis can activate ER stress pathways, which in turn drive inflammatory responses that contribute to disease progression.^512^ Studies on FLS and macrophages from RA synovial fluid have revealed heightened activation of the IRE1/XBP1 axis.^458^ In mice, deletion of IRE1α specifically in myeloid cells reduces cytokine production and protects against inflammatory arthritis, highlighting its role in disease progression.^513^ IRE1α activation enhances TLR-mediated pro-inflammatory cytokine production in macrophages and neutrophils, with its regulated IRE1-dependent decay (RIDD) activity stabilizing cytokine mRNA and sustaining a robust inflammatory response in the RA synovium.^514^

#### UPR signaling in neuroinflammation, oligodendrocyte survival, and demyelination in MS

ER stress is widely observed in demyelinating lesions in MS, with elevated levels of markers such as CHOP, BiP, phosphorylated PERK, phosphorylated eIF2α, and XBP-1 in microglia, oligodendrocytes (OLs), T cells, and astrocytes.^515^ The pro-apoptotic molecule CHOP underscores the connection between ER stress and cell fate in MS, with its expression in various cell types suggesting immune-mediated processes contributing to ER stress-associated damage. UPR activation is particularly prominent in active MS lesions, driven by increased levels of inflammatory cytokines, reactive nitrogen species (RNS), and ROS.

Immunohistochemical analysis of biopsy and postmortem samples revealed increased expression of UPR markers, including CHOP and BiP, in oligodendrocytes, astrocytes, T cells, and macrophages/microglia within MS lesions.^516^ In experimental autoimmune encephalomyelitis (EAE) mice, CNS expression of IFNγ before disease onset prevents oligodendrocyte death, demyelination and axonal degeneration. Modest activation of the PERK-eIF2α pathway in oligodendrocytes of control EAE mice is significantly enhanced by IFNγ, highlighting its protective role in the disease process.^517^ However, PERK heterozygous deficiency exacerbates IFNγ-induced apoptosis of remyelinating oligodendrocytes and impairs remyelination in cuprizone-induced demyelinated lesions.^518^ These findings suggest that activation of the PERK-eIF2α pathway protects (re)myelinating oligodendrocytes from the harmful effects of IFNγ in immune-mediated demyelinating diseases.^519^

### Therapeutic targeting of proteostasis in autoimmune diseases

Autoimmune diseases are characterized by chronic immune activation, inflammatory tissue injury, and failure of immune tolerance. Increasing evidence implicates proteostasis networks as active determinants of immune cell fate, cytokine production, and autoantibody generation. This has motivated therapeutic strategies that modulate proteostasis to recalibrate immune responses rather than solely suppress downstream inflammation.

#### Targeting HSPs in autoimmune diseases

##### HSP90 inhibition and inflammatory control

HSP90 inhibitors have shown notable immunomodulatory activity in preclinical autoimmune models, consistent with the role of HSP90 in stabilizing signaling proteins that amplify immune activation. 17-AAG (tanespimycin) alleviates disease manifestations in SLE mouse models by reducing inflammation and immune cell activation.^520^ Ganetespib similarly reduces inflammation and joint damage in RA models, highlighting its translational potential in chronic synovitis.^521^ 17-DMAG (alvespimycin) has also demonstrated efficacy in autoimmune settings by modulating immune responses and reducing tissue damage.^522^ A mechanistically distinct approach is exemplified by acacetin, an HSP90 ATPase inhibitor that suppresses the folding of cyclooxygenase-2 (COX-2), leading to ubiquitin-mediated COX-2 degradation and a downstream reduction in inflammatory signaling.^523^ Together, these studies support HSP90 inhibition as a viable upstream strategy to dampen pathogenic immune activation.

##### HSPs as biomarkers in autoimmune disease monitoring

Beyond therapeutic targeting, HSP-related immune signatures offer diagnostic and monitoring opportunities. Anti-HSP70 autoantibodies can be detected in saliva and urine using indirect ELISA, including in healthy individuals, providing a non-invasive biomarker modality with potential utility for disease prediction, early detection, and longitudinal monitoring.^524^ The accessibility of such biomarkers is particularly attractive for chronic autoimmune conditions requiring repeated assessment.

##### HSP-based DNA vaccines and tolerance induction

HSP-directed DNA vaccines provide an immunologically sophisticated strategy aimed at restoring tolerance rather than broadly suppressing immunity. DNA vaccines encoding HSP90 reduce lupus manifestations and prolong survival in lupus-prone mice by inducing tolerogenic dendritic cells and expanding regulatory T cells (Tregs), thereby suppressing autoimmune responses.^525^ Similarly, HSP70 DNA vaccination suppresses anti-dsDNA antibody production and reduces renal disease in lupus models.^526^ These approaches highlight the potential of chaperone-centered immune reprogramming to mitigate autoimmunity while preserving protective immune function.

#### Therapeutic modulation of the autophagy-mitophagy axis in autoimmune diseases

##### Autophagy inhibition to restrain inflammatory signaling

CQ and HCQ represent clinically established autophagy inhibitors with broad immunomodulatory actions. Beyond lysosomal deacidification and inhibition of autophagosome-lysosome fusion, CQ/HCQ disrupts endolysosomal function, Toll-like receptor activation, and calcium signaling, thereby reducing pro-inflammatory cytokine production.^527^ In lupus-prone mice, CQ promotes the expansion of Treg populations and suppresses CNS inflammation under both prophylactic and therapeutic dosing regimens.^528^ CQ also modulates the expression and activity of the transcription factor Nurr1, which influences Treg differentiation, providing an additional mechanistic link between autophagy-related pathways and immune tolerance.^528^

Additional experimental inhibitors further support the therapeutic relevance of autophagy blockade in autoimmune settings. Spautin-1 promotes the degradation of Vps34 complexes, key regulators of autophagy initiation, and has shown promise in preclinical autoimmune models.^529^ 3-Methyladenine (3-MA), a widely used inhibitor that blocks autophagosome formation, continues to serve as a mechanistic tool compound for dissecting autophagy’s role in autoimmune pathology.^530^

##### Autophagy induction and immune rebalancing

In contrast, autophagy induction can be therapeutically beneficial when autoimmune pathology is driven by defective clearance programs, immune-complex burden, or mitochondrial damage. Rapamycin has been extensively studied for immunomodulatory effects across autoimmune diseases. In RA, rapamycin inhibits proliferation and induces apoptosis of fibroblast-like synoviocytes via the AKT/mTORC1 pathway, reducing joint inflammation and tissue damage.^531^ In SLE models, rapamycin improves endothelial function and reduces inflammation with long-term efficacy and tolerability.^532^ In MS models, rapamycin rescues Treg function at the blood‒brain barrier interface, highlighting its potential relevance for neuroinflammatory disease.^533^

Metformin also modulates immune responses in lupus and RA.^534^ Mechanistically, metformin enhances autophagy, promoting clearance of immune complexes and dysfunctional organelles, thereby reducing inflammatory burden. Vitamin D has additionally been linked to the restoration of autophagic balance and immunoregulation in SLE, influencing T-cell activity, reducing inflammation, and promoting immune tolerance, supporting its potential as an adjunct therapy.^535^

##### Targeting mitophagy to suppress mitochondrial inflammation

Mitophagy is increasingly recognized as a decisive regulator of autoimmune inflammation, particularly through the control of mtDNA release and mitochondrial ROS. Metformin, as an AMPK activator, inhibits the AMPK-mTOR-STAT3 axis, reduces mtDNA mutations and ROS generation in lupus models, and enhances mitophagy. By limiting mtDNA release, metformin mitigates inflammatory amplification and provides therapeutic benefits in SLE.^536^ NESTIN, which regulates mitochondrial autophagy and oxidative stress, promotes mitophagy and reduces oxidative damage and inflammation, offering a mechanistically distinct approach to maintaining mitochondrial function under autoimmune conditions.^537^ UMI-77, a mitophagy inducer, reduces renal inflammation and damage in lupus-prone mice while restoring mitochondrial function. It also attenuates T-cell proliferation and pro-inflammatory phenotypes in dendritic cell/T-cell co-culture assays, strengthening its rationale as a candidate for autoimmune disease intervention.^538^

#### UPS-targeted therapies in autoimmune diseases

##### MDM2 inhibition

MDM2, an E3 ligase best known for regulating p53 stability, also contributes to immune dysregulation. The MDM2 inhibitor nutlin-3a suppresses abnormal T-cell expansion and reduces systemic inflammation in SLE models, highlighting an immunoregulatory role for MDM2 inhibition beyond oncology.^539^

##### Proteasome inhibitors

Proteasome inhibitors provide another established strategy. Bortezomib and carfilzomib, FDA-approved for cancer, reduce autoantibody levels and suppress IFN-α production by plasmacytoid dendritic cells, slowing SLE progression. Importantly, bortezomib has shown promise in refractory SLE patients,^540,541^ emphasizing the translational relevance of proteasome modulation in severe autoimmune disease.

#### Therapeutic targeting of ER stress pathways in autoimmune diseases

##### IRE1α inhibition

IRE1α inhibitors have demonstrated benefit in preclinical autoimmune models. BI09 prevents XBP1 mRNA splicing, reducing XBP1s production and mitigating ER stress.^542^ 4µ8C reduces mitochondrial ROS production in peripheral neutrophils, decreasing plasma cell expansion and autoantibody production in lupus-prone mice.^543^

##### PERK pathway modulation

Modulation of PERK signaling provides an additional approach. Guanabenz enhances eIF2α phosphorylation, strengthens PERK pathway signaling, protects mice from cytokine shock, and reduces autoimmune symptoms in lupus models.^544^ Salubrinal, which indirectly inhibits eIF2α dephosphorylation, protects cells from ER stress-induced apoptosis and suppresses inflammatory cytokines in RA models. It also inhibits Dusp2, reducing MAPK signaling and inflammation in collagen-induced arthritis.^545^

##### ATF6 and chemical chaperones

ATF6 promotes transcription of ER chaperones and the ERAD machinery. Chemical chaperones such as 4-PBA alleviate ER stress by enhancing folding capacity. In lupus-prone mice, 4-PBA reduces splenomegaly, anti-dsDNA antibodies, and inflammatory cytokines and alleviates lupus nephritis by decreasing albuminuria, renal inflammation, and immune complex deposition.^546,547^

ATF6 is a transcription factor that upregulates chaperones and components of the ERAD machinery. Chemical chaperones such as 4-PBA can alleviate ER stress by enhancing protein folding. 4-PBA is a small-molecule chemical chaperone that improves protein folding capacity, reducing ER stress. In lupus-prone mice, 4-PBA reduces splenomegaly, anti-dsDNA antibodies, and inflammatory cytokines. It also alleviates lupus nephritis by decreasing albuminuria, renal inflammation, and immune complex deposition.^546,547^

## Crosstalk between protein homeostasis machineries: a novel perspective on mechanistic intersections and therapeutic implications

Maintaining protein homeostasis is an intricate and dynamic process regulated by multiple interconnected cellular pathways, including the heat shock response (HSR), autophagy, mitophagy, the UPS, ERAD, and non-canonical mechanisms such as chaperone-mediated autophagy (CMA) and ribosome-associated quality control (RQC).^1^ While traditionally studied in isolation, recent advances reveal extensive crosstalk between these pathways, which coordinate protein turnover, stress responses, and quality control under physiological and pathological conditions. Here, we explore the mechanistic interactions, therapeutic implications, and emerging intersections among these machineries, emphasizing novel and thought-provoking insights that expand the current understanding of proteostasis regulation.

### HSPs and autophagy: a synergistic relationship

HSPs, particularly HSP70 and HSP90, play a pivotal role in protein folding and refolding, preventing the aggregation of misfolded proteins. However, when the protein misfolding burden exceeds the capacity of HSPs, autophagy is activated as a secondary defense mechanism. This interplay is particularly evident in neurodegenerative diseases, where the accumulation of misfolded proteins such as tau, α-synuclein, and huntingtin overwhelms the HSP system, leading to the activation of autophagy as a compensatory mechanism.^1,23^ For example, HSP70 and HSP90 are known to interact with key autophagy regulators such as Beclin-1 and LC3. For instance, HSP70 can bind to misfolded proteins and deliver them to autophagosomes via its interaction with LC3, facilitating their degradation.^548^ Similarly, HSP90 stabilizes the autophagy-initiating kinase ULK1, thereby promoting autophagosome formation.^549^ This crosstalk ensures that when the chaperone system is overwhelmed, autophagy can step in to clear the accumulating toxic proteins. Targeting this intersection could offer dual benefits. For example, HSP90 inhibitors such as ganetespib not only disrupt chaperone function but also enhance autophagy, leading to the degradation of oncogenic proteins in cancer.^54,55^ Similarly, in neurodegenerative diseases, enhancing both HSP70 and autophagy could provide a synergistic approach to clearing protein aggregates.

### Autophagy and mitophagy: coordinated clearance of cellular debris

Autophagy and mitophagy are closely linked processes that ensure the removal of damaged organelles and protein aggregates. Mitophagy, a selective form of autophagy, specifically targets damaged mitochondria for degradation, thereby preventing the release of ROS and subsequent cellular damage.^17,550^ The PINK1-Parkin pathway is a well-characterized mechanism of mitophagy. Under conditions of mitochondrial stress, PINK1 accumulates on the outer mitochondrial membrane, recruiting Parkin, which ubiquitinates mitochondrial proteins (Fig. 1a). These ubiquitinated proteins are then recognized by autophagy receptors such as p62/SQSTM1, linking mitophagy to the broader autophagy machinery.^33^ Additionally, FUNDC1, a mitophagy receptor, interacts with LC3 under hypoxic conditions, further integrating mitophagy with autophagy.^551^ Dysregulation of mitophagy is implicated in diseases such as Parkinson’s disease and cancer. Enhancing mitophagy through pharmacological activation of PINK1-Parkin or FUNDC1 could mitigate mitochondrial dysfunction and reduce oxidative stress. Conversely, inhibiting mitophagy in cancer cells could exacerbate mitochondrial damage and induce cell death.^34,552^

### UPS and autophagy: complementary degradation pathways

The UPS and autophagy are the two primary protein degradation pathways in cells. While the UPS is responsible for the rapid degradation of short-lived and misfolded proteins, autophagy handles long-lived proteins, protein aggregates, and damaged organelles. These pathways often work in concert to maintain proteostasis, especially under stressful conditions. The UPS and autophagy share common regulatory nodes, such as the ubiquitin-binding protein p62/SQSTM1. p62 acts as a bridge between ubiquitinated proteins and the autophagic machinery, facilitating the degradation of ubiquitinated substrates via autophagy.^553^ Additionally, the deubiquitinating enzyme USP14, which regulates proteasome activity, modulates autophagy by controlling the levels of ubiquitinated proteins.^554^ In diseases where the UPS is overwhelmed, such as neurodegenerative disorders, enhancing autophagy could provide an alternative route for protein degradation. Conversely, in cancer, where autophagy is often upregulated to promote survival, inhibiting autophagy while simultaneously targeting the UPS could lead to the accumulation of toxic proteins and induce cell death.^64,555^

### ERAD/UPR and autophagy: managing endoplasmic reticulum stress

The ER is a critical site for protein folding and quality control. When misfolded proteins accumulate in the ER, the UPR is activated to restore homeostasis. If the UPR fails to resolve stress, ERAD targets misfolded proteins for degradation by the proteasome. However, when ERAD is insufficient, autophagy is activated to clear the accumulating proteins. The UPR and autophagy are closely linked through the IRE1 and PERK pathways. IRE1 activation leads to the splicing of XBP1, which upregulates genes involved in protein folding and ERAD. On the other hand, PERK activation phosphorylates eIF2α, reducing protein synthesis and promoting autophagy.^556^ Additionally, ATF6, another UPR sensor, can upregulate autophagy-related genes, further linking the UPR to autophagy. In diseases characterized by chronic ER stress, such as diabetes and neurodegenerative disorders, modulating the UPR and autophagy could provide therapeutic benefits. For example, chemical chaperones such as 4-PBA and TUDCA, which alleviate ER stress, could be combined with autophagy enhancers to improve protein clearance and reduce cellular damage.^176^

### Non-canonical pathways: expanding the proteostasis network

Beyond the canonical pathways, several non-canonical mechanisms contribute to proteostasis. These include CMA, aggrephagy (selective autophagy of protein aggregates), and lysosomal degradation pathways. These pathways often intersect with the major proteostasis machineries, providing additional layers of regulation. CMA, for instance, selectively degrades proteins containing a KFERQ-like motif, with HSP70 playing a crucial role in substrate recognition and delivery to lysosomes.^557^ Aggrephagy, on the other hand, involves the selective degradation of protein aggregates, often mediated by autophagy receptors such as p62 and NBR1.^558^ Targeting non-canonical pathways could offer novel therapeutic strategies. For example, enhancing CMA could provide a specific route for the degradation of toxic proteins in neurodegenerative diseases, while inhibiting aggrephagy in cancer could lead to the accumulation of protein aggregates and induce cell death.^165^

### Crosstalk in disease contexts: a unified perspective

The crosstalk between proteostasis machineries is particularly relevant in the context of disease. For example, in neurodegenerative diseases, the accumulation of misfolded proteins overwhelms the HSP system, leading to the activation of autophagy and mitophagy. However, when these pathways are also impaired, the UPS and ERAD/UPR are activated as compensatory mechanisms. In cancer, the upregulation of HSPs and autophagy promotes tumor survival, while the inhibition of these pathways can induce cell death. In metabolic diseases, the dysregulation of mitophagy and the ERAD/UPR contributes to insulin resistance and mitochondrial dysfunction. Thus, a unified approach that targets multiple proteostasis pathways could provide synergistic benefits. For example, in cancer, combining HSP90 inhibitors with autophagy inhibitors could lead to the accumulation of misfolded proteins and induce cell death. In neurodegenerative diseases, enhancing both HSP70 and autophagy could provide a dual approach to clearing protein aggregates.

### Drug repurposing and novel therapeutic strategies

The crosstalk between proteostasis machineries offers numerous opportunities for drug repurposing. For example, metformin has been shown to enhance autophagy and mitophagy, making it a potential candidate for neurodegenerative diseases.^60^ Similarly, rapamycin, an mTOR inhibitor, promotes autophagy and has shown promise in cancer and neurodegenerative diseases.^60^ Emerging technologies such as PROTACs and AUTACs offer novel ways to target specific proteins for degradation via the UPS or autophagy, respectively. These approaches could be combined with existing therapies to enhance their efficacy.^66,168^

In conclusion, the crosstalk between different protein homeostasis machineries is a complex yet highly coordinated process that ensures cellular survival under stress conditions. Understanding the mechanistic intersections between HSPs, autophagy, mitophagy, the UPS, and the ERAD/UPR offers novel insights into disease mechanisms and provides a rich landscape for therapeutic innovation. By targeting these interconnected pathways, we can develop more effective and synergistic treatments for a wide range of diseases, ultimately enhancing proteostasis resilience and improving patient outcomes.

## Conclusion and perspectives: towards a systems-level understanding of proteostasis

Over the past decade, proteostasis research has evolved from studying isolated degradation pathways to appreciating a deeply interconnected, adaptive, and dynamic cellular defense network. Rather than functioning as independent mechanisms, HSPs, autophagy, mitophagy, the UPS, ERAD, and UPR collectively form a multilayered, compensatory system that preserves protein quality and cellular viability. As highlighted throughout this Review, failures in this network do not simply reflect isolated molecular defects; instead, they represent a collapse in proteostasis resilience, the intrinsic ability of cells to sense stress, mobilize coordinated responses and restore equilibrium across multiple proteostatic axes. Moving forward, understanding proteostasis at a systems level, as an interactome rather than a pathway, will be essential for decoding disease mechanisms and designing truly effective therapeutic interventions.

### Mapping the proteostasis network: from pathways to interactomes

A fundamental challenge ahead is to move from pathway-level knowledge toward a comprehensive proteostasis interactome, capturing how proteostasis systems communicate, compensate and fail under disease stress. Modern multiomics platforms are now uniquely positioned to achieve this transition.

#### Proteomics and interactomics

High-resolution proteomic technologies, including mass spectrometry and affinity purification coupled with mass spectrometry (AP-MS), enable systematic identification of protein‒protein interaction networks and post-translational regulatory nodes. These studies have already revealed expanded roles for classical chaperone systems. For example, mapping the interactomes of HSP70 and HSP90 has clarified that these proteins not only ensure correct folding but also influence autophagic decision-making and UPS performance,^548,549^ highlighting their integrative role in proteostasis governance. Likewise, p62/SQSTM1 interactome analyses have elucidated how it couples ubiquitinated substrates to the autophagic machinery, placing it at a crucial signaling intersection.^553^

#### Spatial proteomics

Beyond interaction networks, spatial proteomics adds an essential layer of biological meaning. Cellular proteostasis is governed not only by molecular availability but also by spatial proximity and compartmentalization. Technologies such as BioID and APEX labeling are uncovering how subcellular localization influences proteostasis pathway coordination. The localization of FUNDC1 at ER-mitochondria contact sites (MAMs) and its role in integrating mitophagy, mitochondrial adaptation, and ER stress responses exemplify how spatial organization determines functional proteostasis outcomes.^551^ Understanding these spatial dynamics will be indispensable for deciphering how cells coordinate proteostasis surveillance across organelles and how diseases disrupt these positional networks.

### CRISPR-based screening: identifying key regulatory nodes

CRISPR-based genetic screening has transformed the proteostasis field by enabling unbiased identification of essential regulatory components and adaptive signaling hubs. Genome-wide knockout, knockdown, and activation screens are now systematically revealing both canonical and unexpected regulators of autophagy, mitophagy, UPS function, and ER stress adaptation.

#### Functional genomics

For instance, CRISPR screens in the PINK1-Parkin mitophagy pathway have identified novel cofactors and signaling partners, expanding our understanding of mitochondrial quality control and offering promising therapeutic entry points in disorders such as Parkinson’s disease.^33^ Similarly, systematic screening under ER stress conditions continues to uncover new UPR modulators that could be pharmacologically leveraged in metabolic and inflammatory pathologies.^556^ These efforts illustrate how functional genomics is redefining the proteostasis landscape.

#### High-throughput phenotyping

Importantly, the integration of CRISPR screening with high-content phenotyping modalities, including live-cell imaging, multiplex transcriptomics, and single-cell RNA sequencing, enables real-time visualization of how cells dynamically reorganize proteostasis resources. Such platforms have demonstrated how proteasome inhibition induces a functional handover toward autophagy-dependent degradation,^9^ reflecting intrinsic compensatory resilience. These tools therefore not only identify genetic regulators but also illuminate systems-level adaptation principles that can be therapeutically harnessed.

### Machine learning and artificial intelligence: predictive modeling of proteostasis

The complexity of proteostasis networks necessitates analytical approaches capable of capturing nonlinear behavior, feedback regulation, and emergent properties. AI and machine learning (ML) offer precisely this capacity. By integrating multilayered datasets from transcriptomics, proteomics, metabolomics, and CRISPR screens, ML approaches can construct predictive models of proteostasis dynamics, allowing researchers to visualize how stress propagates through cellular networks.

#### Network modeling

Graph neural networks and other advanced computational tools now enable simulations wherein perturbation of a single node, for example, HSP90 inhibition, can be computationally modeled to predict downstream shifts in UPS engagement or autophagy induction.^559^ These predictions inform rational design of combination therapies that strategically exploit compensatory relationships rather than acting blindly upon isolated mechanisms.

#### Drug discovery

AI-based drug discovery is also reshaping therapeutic development. Machine learning-driven compound prediction platforms have successfully identified novel HSP90 inhibitors with clinical potential.^12^ Meanwhile, computational design approaches are accelerating the development of PROTACs and AUTACs, enabling targeted degradation of disease-associated proteins through UPS or autophagy, respectively.^66,168^ Such tools represent a paradigm shift toward precision proteostasis engineering.

### Proteostasis resilience: a new framework for disease intervention

Across disease systems, one recurring theme emerges: the difference between cellular survival and failure is determined not only by the existence of proteostasis pathways but also by their resilience capacity and their ability to respond robustly, flexibly, and coordinately. Healthy cells dynamically recruit chaperones, activate stress responses, mobilize UPS function, and initiate autophagy and mitophagy as integrated responses to stress. Disease progression often reflects either an inability to mount such a coordinated response or a maladaptive over-activation that disrupts homeostatic balance.

Strengthening resilience therefore provides a powerful translational paradigm. Induction of protective stress responses, such as the HSR and UPR, enhances chaperone buffering capacity and promotes adaptive autophagy induction.^13^ Metabolic sensing circuits such as the AMPK-mTORC1 axis further ensure that cells strategically distribute proteostasis demand between proteasome activity and autophagic sequestration according to nutrient context.^24^ In neurodegeneration, where proteostasis collapse is strongly associated with disease progression, enhancing resilience through multi-pathway modulation may slow pathological decline.^1^ In contrast, in cancer, where malignant cells exploit heightened proteostasis resilience to tolerate genomic instability and therapeutic stress, strategically destabilizing resilience may enhance therapeutic vulnerability.

Thus, resilience-centered thinking shifts proteostasis therapeutics from single-target correction toward network stabilization or context-specific destabilization, precisely aligned with disease biology.

### Personalized medicine: tailoring proteostasis therapies to individual patients

Finally, the convergence of proteostasis biology, computational analytics and clinical science is pushing the field toward precision proteostasis medicine. Rather than assuming uniform proteostasis failure mechanisms across individuals, single-cell omics and AI-guided analytics now allow the development of individualized proteostasis signatures, capturing differences in pathway dominance, compensatory potential and disease vulnerability.

Proteostasis biomarkers, including HSP expression profiles, autophagy flux indicators, ubiquitinated protein accumulation, and p62/SQSTM1 levels, are increasingly informative regarding prognosis and therapeutic responsiveness. For example, elevated p62/SQSTM1 can identify tumors resistant to proteasome inhibition, guiding clinicians toward autophagy-targeted strategies.^555^ Integrating these biomarkers with computational network modeling will support the development of precision intervention strategies, such as mitophagy enhancers in conditions with PINK1-Parkin inefficiency or ER stress-modulating agents in ERAD-deficient diseases.^176^

Collectively, these advances underscore a fundamental conceptual shift: proteostasis must now be viewed not as a collection of isolated degradation mechanisms but as a coherent, adaptive, and deeply integrated cellular defense network whose resilience determines disease trajectory. Moving toward a systems-level understanding, empowered by interactomics, CRISPR technologies, AI-driven modeling and precision biomarker science, will profoundly transform how we interpret disease biology and develop therapeutics. Embracing proteostasis resilience as a central organizing principle provides both a conceptual lens and a translational strategy, guiding the development of next-generation interventions capable of restoring or strategically modulating equilibrium across diverse and complex disease states.
